# Complex regulation of Hsf1-Skn7 activities by the catalytic subunits of PKA in *Saccharomyces cerevisiae*: experimental and computational evidences

**DOI:** 10.1186/s12918-015-0185-8

**Published:** 2015-07-27

**Authors:** Sergio Pérez-Landero, Santiago Sandoval-Motta, Claudia Martínez-Anaya, Runying Yang, Jorge Luis Folch-Mallol, Luz María Martínez, Larissa Ventura, Karina Guillén-Navarro, Maximino Aldana-González, Jorge Nieto-Sotelo

**Affiliations:** Instituto de Biología, Universidad Nacional Autónoma de México, 04510 México, D.F. Mexico; Instituto de Ciencias Físicas, Universidad Nacional Autónoma de México, 62210 Cuernavaca, Morelos Mexico; Instituto de Biotecnología, Universidad Nacional Autónoma de México, 62210 Cuernavaca, Morelos Mexico; Present Address: Department of Anesthesiology, Pharmacology & Therapeutics, The University of British Columbia, Vancouver, V6T 1Z4 BC Canada; Present Address: Centro de Investigación en Biotecnología, Universidad Autónoma del Estado de Morelos, 62209 Cuernavaca, Mor. Mexico; Present Address: Grupo La Florida México, Tlalnepantla, 54170 Edo. de Méx. Mexico; Present Address: El Colegio de la Frontera Sur, 30700 Tapachula, Chis. Mexico

**Keywords:** Yeast, Signal transduction, Hsf1 function, Skn7 function, Windowed discrete model

## Abstract

**Background:**

The cAMP-dependent protein kinase regulatory network (PKA-RN) regulates metabolism, memory, learning, development, and response to stress. Previous models of this network considered the catalytic subunits (CS) as a single entity, overlooking their functional individualities. Furthermore, PKA-RN dynamics are often measured through cAMP levels in nutrient-depleted cells shortly after being fed with glucose, dismissing downstream physiological processes.

**Results:**

Here we show that temperature stress, along with deletion of PKA-RN genes, significantly affected HSE-dependent gene expression and the dynamics of the PKA-RN in cells growing in exponential phase. Our genetic analysis revealed complex regulatory interactions between the CS that influenced the inhibition of Hsf1/Skn7 transcription factors. Accordingly, we found new roles in growth control and stress response for Hsf1/Skn7 when PKA activity was low (*cdc25Δ* cells). Experimental results were used to propose an interaction scheme for the PKA-RN and to build an extension of a classic synchronous discrete modeling framework. Our computational model reproduced the experimental data and predicted complex interactions between the CS and the existence of a repressor of Hsf1/Skn7 that is activated by the CS. Additional genetic analysis identified Ssa1 and Ssa2 chaperones as such repressors. Further modeling of the new data foresaw a third repressor of Hsf1/Skn7, active only in theabsence of Tpk2. By averaging the network state over all its attractors, a good quantitative agreement between computational and experimental results was obtained, as the averages reflected more accurately the population measurements.

**Conclusions:**

The assumption of PKA being one molecular entity has hindered the study of a wide range of behaviors. Additionally, the dynamics of HSE-dependent gene expression cannot be simulated accurately by considering the activity of single PKA-RN components (i.e., cAMP, individual CS, Bcy1, etc.). We show that the differential roles of the CS are essential to understand the dynamics of the PKA-RN and its targets. Our systems level approach, which combined experimental results with theoretical modeling, unveils the relevance of the interaction scheme for the CS and offers quantitative predictions for several scenarios (WT vs. mutants in PKA-RN genes and growth at optimal temperature vs. heat shock).

**Electronic supplementary material:**

The online version of this article (doi:10.1186/s12918-015-0185-8) contains supplementary material, which is available to authorized users.

## Background

The cyclic AMP (cAMP)-dependent protein kinase (PKA) regulatory network (PKA-RN) is found in protozoa, animals, algae, and fungi. It plays a central role in the control of different inter- and intra- cellular processes such as metabolism, cell proliferation, stress response, and development [57, 84]. In yeast, the PKA-RN is also involved in the control of growth in response to nutrient conditions, which in turn are known to influence the stress response [84].

In *S. cerevisiae*, the PKA holoenzyme forms an inactive tetramer composed of two regulatory subunits (2 x Bcy1) [85] and two out of three CS (Tpk1, Tpk2, or Tpk3) [86]. When intracellular concentrations of cAMP increase, Bcy1 binds cAMP, promoting the activation by release of the CS. Two proteins, Gpa2 and Ras2, regulate adenylate cyclase, which catalyzes the synthesis of cAMP [14, 43, 84]. Formation of a Ras2⋅GTP complex [22], the active state of Ras2, requires the GDP-GTP exchange activity of Cdc25 [6]. Moreover, the intracellular concentrations of cAMP are also controlled by the phosphodiesterases Pde1 and Pde2 [84]. The low affinity phosphodiesterase Pde1 reduces cAMP levels in nutrient depleted cells soon after glucose addition [55], whereas the high affinity phosphodiesterase Pde2 lowers cAMP levels during the exponential and stationary phases of growth [62]. It is thought that the activity of the pathway increases at high levels of glucose (or other rapidly fermentable sugars) and declines when the cells deplete the sugars, or when entering stationary phase [84]. Therefore, the PKA activity is influenced by the amount of fermentable sugars and by the growth phase of the culture.

The growth phase of yeast liquid cultures impacts their level of thermotolerance. For instance, during the exponential phase cells are stress sensitive, whereas during the stationary phase they develop stress resistance [67, 91]. This behavior has been studied using genetic analysis. Stress resistance is explained through a reduced activity of the Ras-cAMP branch of the pathway (such as in *cdc25Δ, cyr1* or *ras1 ras2*^*ts*^ strains). These mutants grow slowly and show elevated basal thermotolerance during exponential phase [23, 25, 42, 64, 83]. In contrast, mutants with high PKA activity, such as *ira2, pde2, bcy1* or *RAS2*^*val19*^, are very sensitive to temperature stress [19, 55, 62, 84, 87]. In exponential phase, basal thermotolerance is negatively regulated by the Rim15 protein kinase [67]. However, acclimation to high temperatures during the exponential phase requires the concerted action of Hsf1 and Msn2/Msn4 transcription factors, and chromatin remodeling complexes such as SWI/SNF [3, 18]. These factors allow the rapid transcription of genes encoding stress proteins involved in prevention and repair of damages caused by stress [3, 19, 33].

Hsf1 transcription factor is encoded by a single gene [76] and shows high affinity for the heat shock elements (HSE), found in the promoters of the heat shock genes [21]. The essentiality of Hsf1 indicates that — besides being important for the response to carbon starvation as well as heat, osmotic, and oxidative stress — it also plays important functions in normal growth [3, 4, 76, 81]. The widespread functions regulated by Hsf1 explain its binding to a large number of promoters (about 3 % of the genes in the yeast genome). Among the functions of its targets are: protein folding, degradation, trafficking, cell integrity maintenance, transport, signaling, and transcription [33]. Hsf1 contains DNA binding and trimerization domains and is hyper-phosphorylated in serine and threonine residues in response to heat and oxidative stress [39, 76], modifications that activate its transcriptional activity [39, 76]. The PKA constitutively represses the activity of Hsf1, thereby inhibiting the expression of small heat shock protein genes [20]. It is documented that, in this regulation, the CS of PKA do not interact directly with Hsf1 [20]. Moreover, when the activity of the PKA is low, such as during glucose starvation, Hsf1 is phosphorylated and activated by Yak1 and Rim15 kinases [50, 51]. However, the factors that mediate the regulation of Hsf1 by the PKA in glucose-rich media, and in response to heat shock, are still unknown. In addition to Hsf1, Skn7 also recognizes HSE elements [66] and is part of a two-component system required for the signaling of the hypo-osmotic stress and the oxidative stress pathways [49, 82]. Previous reports have shown that the activity of Skn7 during the oxidative stress response is negatively-regulated by the PKA-RN [10].

Recently, it has become evident that results based only in experimental approaches, and the static models derived from them, are not sufficient to fully understand the complex dynamics of a cellular system. Rather, the integration of experimental data with dynamical modeling has expanded our current knowledge of the cell by enabling the prediction of hidden cellular behaviors. Thus, computational modeling is becoming an indispensable tool to comprehend the organization of biological systems [48, 80] and the analysis of the dynamics of the PKA-RN is no exception. Some studies of the PKA-RN considered only its core components and focused on the feed-back regulation of cAMP levels that nutrient-depleted cells display during the short-term response (i.e., seconds) to a pulse of glucose [8, 63, 93]. More recently, PKA-RN models simulate long-term growth (i.e., hours) in glucose [28] and evaluate targets downstream of the PKA [24, 29]. However, in all these models the activity of the three CS (Tpk1, Tpk2, and Tpk3) is considered as a single entity, assumption that might be correct in certain scenarios. Nonetheless, in most situations, this assumption could be misleading, as it is known that each CS has unique target specificities [65]. Furthermore, the CS regulate certain physiological processes in an antagonistic fashion [61, 68, 69], complicating even more the prediction of the dynamics of the PKA-RN.

**Fig. 1:**
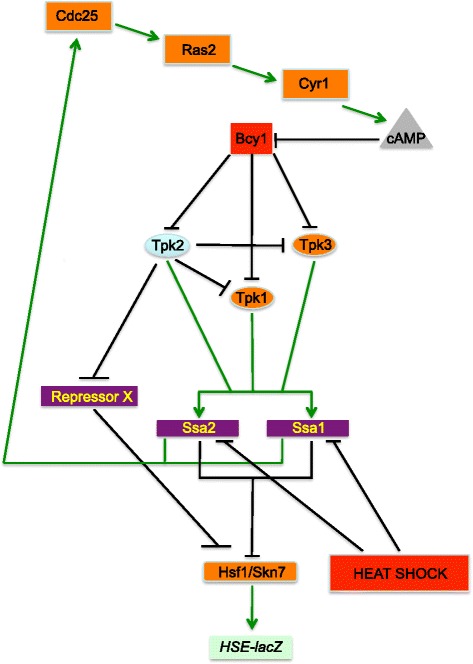
Scheme for the development of a dynamic computational model for the simulation of the regulation of HSE-dependent gene expression by the PKA-RN. A PKA-RN composed of 15 elements was simulated in a dynamic computational model (see Additional file 3: Supplementary Information). In this scheme Cdc25, Ras2, Cyr1, Tpk1, Tpk3, Hsf1, and Skn7 act as positive regulators. Bcy1, an unidentified repressor of Hsf1/Skn7 (Repressor X), cAMP, and heat shock act as repressors. Interestingly, in this scheme Ssa1, Ssa2, and Tpk2 interactions are complex acting both as activators and as repressors. The experimental evidence that accounts for the activities and interactions of the components is described in the Background and Results and discussion sections

In this work, we performed a genetic analysis of the Cdc25-Ras2 branch and some downstream components of the PKA-RN (Fig. 1). We then incorporated these results into a dynamic computational model, to further understand the mechanistic nature of the network. HSE-dependent gene expression was chosen as the end product of the PKA-RN and the performance of cells growing exponentially in glucose-rich media, both at optimal temperature and in response to heat shock, was evaluated. We tested how the different PKA subunits (regulatory and each CS) interacted with each other. Novel interactions, in addition to those already known, are described. Computational modeling of the PKA-RN was performed by extending the well-established “discrete dynamics modeling framework” [1, 38, 71, 90] in order to take into account the fact that gene expression measurements of batch cultures average out individual expression patterns. We named this extension, the Windowed Discrete Model (WDM) because it averages over a given time window the discrete values of the network elements in a given attractor, and weighs this average by the size of the corresponding basin of attraction. This process incorporates the whole set of steady states of the network and captures the inherent averages in population measurements. Although discrete dynamic models are intended to describe expression patterns at the single-cell level, our approach allowed us to make quantitative predictions of gene expression patterns taken at the population level for both WT and mutant strains. Furthermore, we showed that when the population average inherent to batch cultures was implemented in the WDM, the results were similar regardless of the use of synchronous or asynchronous updating of the network elements.

Our genetic analysis showed that the PKA-RN controls HSE-dependent gene expression via Hsf1/Skn7 transcription factors. Modeling the control of Hsf1-Skn7 by the PKA-RN predicted the existence of a repressor connecting the CS with Hsf1-Skn7 and encouraged new genetic analyses that proved that Ssa1 and Ssa2 chaperones repress Hsf1/Skn7 when activated by the CS of PKA. Additionally, novel functions of Skn7 and of the C-terminal domain of Hsf1, such as growth control, thermotolerance, and resistance to H_2_O_2_, were revealed whenever the activity of PKA was low. Our model also predicted the existence of a still unidentified third repressor of Hsf1/Skn7, active only in the absence of Tpk2. The WDM explained and predicted HSE-dependent gene expression in WT and mutant strains with and without high temperature stress. We believe that our WDM of the PKA-RN can be useful to simulate other biological processes where the CS of PKA show similar antagonistic interactions, such as in the control of pseudohyphal growth or iron uptake [61, 68, 69].

Without further adaptations, the WDM is, to our knowledge, the first suitable tool based on discrete dynamics that can be used to simulate data obtained from population level measurements (batch cultures), despite of their known heterogeneity at the physiological and gene expression levels [23, 40, 53].

## Results and discussion

Analysis of gene expression and dynamical modeling of the PKA-RN was performed during exponential growth. Measurements were taken under optimal temperature and after a heat shock at 39 °C (see Methods). The regulation of stress gene expression depends on complex transcriptional mechanisms. For example, in *S. cerevisiae* Msn2, Msn4, Hsf1, Yap1, and eight additional transcription factors contribute to the transcription of heat shock genes [95]. The PKA-RN also controls stress gene expression by inhibiting the activity of Msn2, Msn4, Hsf1, Yap1, and Skn7 [10, 20, 37, 75]. Because of this complexity, we decided to focus on the transcription factors Hsf1 and Skn7 in WT and PKA-RN deletion mutants by measuring the activity of an *HSE-CYC1-lacZ* reporter gene construct to test their *in vivo* activity (see Methods), as reported before [4, 47, 50, 58]. In our hands, this reporter showed no activity in the absence of the HSE and its activity did not correlate with the plasmid copy number in the different strains analyzed (see Methods). Because the effect on HSE-dependent expression by deletions in PKA-RN genes is dependent on the genetic background ([17, 56], and our unpublished data), all mutants used in this work were derivatives of the same laboratory strain (W303). Previous studies have shown that in W303, the expression of several stress genes such as *HSP104*, *TPS1*, *CTT1*, *GPD1*, *HSP12*, and *HSP26* are inhibited by PKA [20, 23] and, in the case of *HSP12* and *HSP26*, their inhibition by PKA is mediated through Hsf1 [20].

### Cdc25 positively regulates HSE-dependent gene expression

**Fig. 2:**
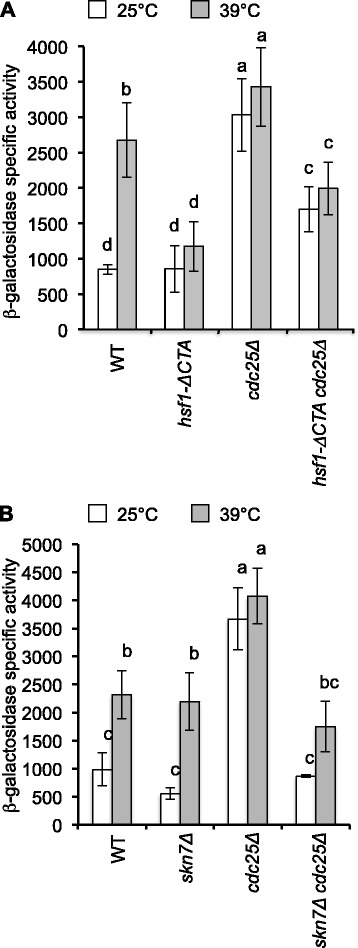
De-repression of HSE-dependent gene expression in *cdc25Δ* cells is dependent on both Hsf1 and Skn7 activities. Strains transformed with reporter plasmid pRY016 (2 μ) were grown in SD medium at 25 °C until mid-exponential phase and treated at different temperatures as described in Methods section. Values are reported as β-galactosidase specific activity (nmol of hydrolyzed ONPG min^−1^ mg^−1^ protein) and are the average and standard deviation of at least three independent experiments. Bars that do not share at least a common letter differ significantly (*P* < 0.05). Strains assayed were: **a** WT (W303-6B), *hsf1-ΔCTA* (LM020), *cdc25Δ* (SL5001), and *hsf1-ΔCTA cdc25Δ* (SL6001). **b** WT (W303-6B), *skn7Δ* (SE1000), *cdc25Δ* (SL5001), and *cdc25Δ skn7Δ* (SL4001)

*CDC25* deletion caused strong alterations in two well-known PKA-regulated processes: growth rate (decreased) and basal thermotolerance (increased) (Additional file 1: Table S1). HSE-driven β-galactosidase activity at 25 °C was 3.7-fold higher in *cdc25Δ* cells than in the WT strain (Fig. 2b). After heat shock, the WT strain increased the reporter activity 2.3-fold relative to the 25 °C condition. In *cdc25Δ* cells, β-galactosidase activity remained unchanged at both temperatures; noteworthy these levels were significantly higher than in the WT at 39 °C. These results indicate that *CDC25* down-regulates HSE-dependent gene expression in WT cells and they are consistent with previous findings showing that PKA inhibits Hsf1 activity [20].

### Hsf1 and Skn7 mediate the high basal thermotolerance and constitutive HSE-dependent gene expression in *cdc25Δ* cells

Both Hsf1 and Skn7 transcription factors recognize HSEs [66, 76]. Therefore, we separately evaluated their contributions to the constitutively-elevated HSE-dependent expression in *cdc25Δ* cells. An Hsf1 lacking 250 residues at the C-terminal domain (*hsf1-ΔCTA*) was used instead of a full deletion of the ORF, because the function of *HSF1* is essential [60, 76]. At 25 °C the β-galactosidase activity in the *hsf1-ΔCTA* strain equated that of the WT but, unlike the WT, after a heat shock at 39 °C its β-galactosidase activity did not increase (Fig. 2a). This confirms that the C-terminal activation domain is required to elevate Hsf1 transcriptional activity in response to heat shock [60]. Furthermore, β-galactosidase levels in the double mutant *hsf1-ΔCTA cdc25Δ* decreased significantly compared to the single *cdc25Δ* mutant, both at 25 °C and after heat shock, supporting the idea that Cdc25 regulates Hsf1 (Fig. 2a). Accordingly, the basal thermotolerance (15 %) of *hsf1-ΔCTA cdc25Δ* cells decreased relative to the *cdc25Δ* single mutant (70 %) (Additional file 1: Table S2). Although basal thermotolerance of *hsf1-ΔCTA* mutant was similar to the WT strain, its duplication time at 25 °C increased slightly (Additional file 1: Table S2). We also found that deletion of the C-terminal domain of Hsf1 suppressed the lack of growth of *cdc25Δ* cells in acetate or galactose at 25 °C. We suggest that the C-terminal domain of Hsf1 plays a negative role in the control of growth in non-fermentable media under conditions of low PKA activity. In yeast, humans and in Arabidopsis, Hsp70 interacts with the C-terminal activation domain of Hsf1 inhibiting its transcriptional activity [4, 45, 73]. We predict that the transcriptional activity and the growth-promoting potential of the full-length Hsf1, when the cell is under under low PKA conditions, could be re-established by deletion of genes encoding Hsp70.

**Fig. 3:**
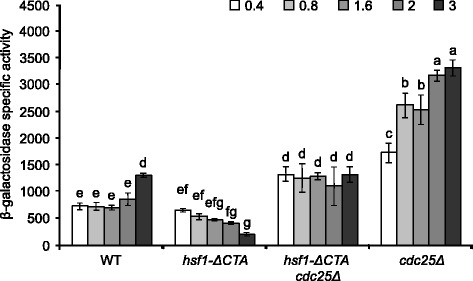
Increase of HSE-dependent gene expression, during the post-diauxic phase of liquid cultures at 25 °C, requires Hsf1 activity. Strains containing plasmid pRY016 were grown in SD medium at 25 °C and aliquots were taken at the indicated culture densities (OD_600_). Data shown represent the average and standard deviation of at least three independent experiments. β-galactosidase specific activities are reported as in Fig. 2. Bars that do not share at least a common letter differ significantly (*P* < 0.05). Strains assayed were: WT (W303-6B), *hsf1-ΔCTA* (LM020), *hsf1-ΔCTA cdc25Δ* (SL6001), and *cdc25Δ* (SL5001)

In WT cells, HSE-dependent expression increased at the beginning of the post-diauxic phase (Fig. 3). This observation agrees with the decline of PKA activity at this stage [84]. A similar pattern was observed in *cdc25Δ* cells, although their initial activity was already very high. Interestingly, the β-galactosidase activity in the double mutant *hsf1-ΔCTA cdc25Δ* was smaller than the activity in *cdc25Δ* cells, remaining constant during the exponential and postdiauxic phases. This indicates that the CTA domain of Hsf1 is required for maximal activity in low PKA cells. Unexpectedly, β-galactosidase levels in the *hsf1-ΔCTA* strain declined steadily as the culture advanced from exponential to the post-diauxic phase (Fig. 3). These observations reinforce the idea that Hsf1 activity is essential to enter the post-diauxic phase at optimal temperatures. Thus, the C-terminal domain of Hsf1 plays four novel functional roles at 25 °C when PKA activity is low: i) increases basal thermotolerance (Additional file 1: Table S3), ii) increases HSE-dependent gene expression (Figs. 2a and 3), iii) causes growth arrest in acetate, iv) causes growth arrest in galactose. These functions of the C-terminal domain of Hsf1 were not previously described [60, 76].

To analyze the contribution of Skn7, the double mutant *skn7Δ cdc25Δ* was also transformed with reporter plasmid pRY016. The β-galactosidase activity in *skn7Δ cdc25Δ* cells at 25 °C or after heat shock was lower than that of *cdc25Δ* cells (Fig. 2b). In contrast to *cdc25Δ hsf1-ΔCTA* cells, β-galactosidase activity increased upon heat shock at 39 °C. However, this increase was not statistically significant (Fig. 2b). This indicates that, in the *cdc25Δ* strain, HSE-dependent expression is reliant on Skn7 for optimal temperature growth to a greater extent than after a heat shock. Furthermore, the basal thermotolerance and the duplication time of *cdc25Δ skn7Δ* cells decreased relative to *cdc25Δ* cells (Additional file 1: Table S2), while the inhibition of growth at 36 °C and in acetate or galactose as sole carbon sources at 25 °C were suppressed by *SKN7* deletion. In agreement with the involvement of Skn7 in the oxidative stress response [49], we observed that resistance of *cdc25Δ* cells to H_2_O_2_ decreased by deletion of *SKN7* (data not shown). The activity of the reporter gene in the single *skn7Δ* mutant was similar to the WT at 25 °C and after a heat shock at 39 °C (Fig. 2b). Together, these results indicate that, in cells growing at optimal temperature or when their PKA activity is low, Skn7 is required to achieve maximal basal thermotolerance and HSE-dependent gene expression. The contribution of Skn7 to the elevated HSE-dependent gene expression in response to heat shock was only marginal (Fig. 2b). Thus, heat induction of HSE-dependent gene expression in cells with low or high PKA activity depends mostly on Hsf1. However, we found that Skn7 plays new roles in other cellular processes at low PKA activity: i) inhibits growth at 25 °C, ii) It is required for H_2_O_2_ resistance, iii) causes growth arrest in glucose at 36 °C, iv) causes growth arrest in acetate at 25 °C, v) causes growth arrest in galactose at 25 °C.

### Ras2 also regulates HSE-dependent gene expression

**Fig. 4:**
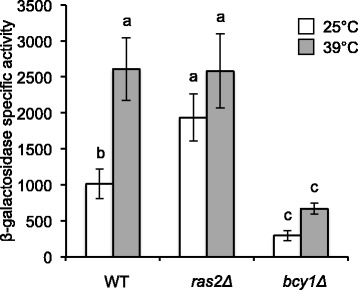
Effect of *RAS2*, and *BCY1* deletions on HSE-dependent gene expression. Strains were transformed with plasmid pRY016 (2 μ) containing an *HSE-CYC1-lacZ* reporter gene. Growth and temperature treatments were performed as described in Methods section. Data shown represent the average and standard deviation of at least three independent experiments. β-galactosidase specific activities are reported as in Fig. 2. Bars that do not share at least a common letter differ significantly (*P* < 0.05). Strains assayed were: WT (W303-1a), *ras2Δ* (Wras2*Δ*) and *bcy1Δ* (CM0095)

Ras2 is a positive regulator of the PKA-RN acting downstream of Cdc25. In a *RAS2* deletion mutant, basal thermotolerance was 120-fold higher than in the WT strain [P = 0.002] (Additional file 1: Table S1). This difference was consistent with a constitutively elevated HSE-dependent gene expression at 25 °C (Fig. 4). Growth rate of the *RAS2* mutant was similar to the WT strain (Additional file 1: Table S1). The increased thermotolerance of *CDC25* and *RAS2* single mutants (Additional file 1: Table S1) confirmed that their PKA activity decreased. However, the growth rate diminished only in the *CDC25*, but not in the *RAS2* mutant. This finding indicates that the control of basal thermotolerance is more sensitive to a low PKA cellular activity than duplication time is.

### Deletion of *BCY1* represses the HSE-dependent gene expression

To evaluate whether cells with high PKA activity altered HSE-dependent gene expression in the opposite way to mutants with low PKA activity, such as *cdc25Δ* and *ras2Δ,* a deletion mutant of *BCY1* was studied. Indeed, HSE-dependent expression was repressed in *bcy1Δ* cells relative to the WT strain at 25 °C and after heat shock at 39 °C (Fig. 4). Consistent with these results, duplication time decreased in the *bcy1Δ* mutant, while basal thermotolerance remained the same as in the WT strain (Additional file 1: Table S1). Induced thermotolerance decreased dramatically in *bcy1Δ* cells (0.22 ± 0.4 % in the mutant vs. 72 ± 12 % in the WT with a P = < 0.001). Moreover, cell viability in *bcy1Δ* cells was very poor, in agreement with previous results [85].

### Tpk1 and Tpk3 inhibit HSE-dependent gene expression in the absence of Tpk2

**Fig. 5:**
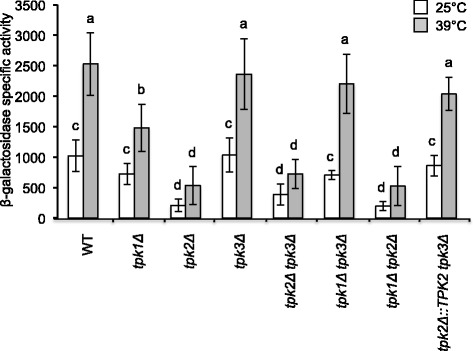
Effect of *TPK* gene deletions on HSE-dependent gene expression. Strains were transformed with plasmid pRY016 (2 μ) containing an *HSE-CYC1-lacZ* reporter gene. Growth and temperature treatments were performed as described in Methods section. Values are reported as β-galactosidase specific activity (nmol of hydrolyzed ONPG min^−1^ mg^−1^ protein) and are the average and standard deviation of at least three independent experiments. Bars that do not share at least a common letter differ significantly (*P* < 0.05). Strains assayed were: WT (W303-1a), *tpk1Δ* (KG712), *tpk2Δ* (KG604), *tpk3Δ* (KS580), *tpk2Δ tpk3Δ* (KS590), *tpk1Δ tpk3Δ* (KS700), *tpk1Δ tpk2Δ* (KS710), *tpk2Δ:: TPK2 tpk3Δ* (KS590-URA3-TPK2)

To explore the possible differences between the CS of PKA, we first analyzed HSE-dependent expression in single *TPK* gene deletion mutants. In *tpk1Δ* cells HSE-dependent expression was slightly reduced at 39 °C but not at 25 °C when compared to the WT (Fig. 5). In *tpk3Δ* cells HSE-dependent expression was not affected. Interestingly, HSE-dependent expression in the *tpk2Δ* mutant was highly repressed both at 25 °C and 39 °C. The basal thermotolerance of the three single mutants was similar to the WT strain (Additional file 1: Table S3). Duplication times of *tpk2Δ* or *tpk3Δ* mutants were similar to the WT strain. However, the *tpk1Δ* mutant showed a slower growth rate (Additional file 1: Table S3). Induced thermotolerance was reduced relative to WT in *tpk1Δ* and *tpk2Δ* mutants, but not in *tpk3Δ*. These results suggested that each CS plays a different role in the control of HSE-dependent gene expression, growth, and in basal- and induced-thermotolerance. In order to analyze the role of individual Tpk’s, double *TPK* deletion mutants were studied. The β-galactosidase activities of *tpk1Δ tpk3Δ* cells growing at 25 °C or after heat shock at 39 °C were similar to their isogenic WT strain (Fig. 5). However, its basal thermotolerance and duplication time increased relative to the WT strain (Additional file 1: Table S3). In contrast, the β-galactosidase activities at 25 and 39 °C in cells containing only Tpk1 (*tpk2Δ tpk3Δ*) or Tpk3 (*tpk1Δ tpk2Δ*) were very low (Fig. 5), whereas their basal thermotolerance and duplication time were similar to the WT. However, the level of induced thermotolerance of *tpk1Δ tpk2Δ* was lower [P = 0.05] than in WT cells (Additional file 1: Table S3). In *tpk2Δ tpk3Δ* and *tpk1Δ tpk3Δ* cells, the induced thermotolerance levels were similar to the WT cells, supporting the idea that Tpk3 and Tpk1 hyper-repress the HSE-dependent gene expression when acting as the sole PKA CS, and that Tpk3 represses the induced thermotolerance if acting as sole PKA CS. These results confirm the hypothesis that the activities of the CS are not redundant for the control of HSE-dependent gene expression, growth, basal or induced thermotolerance. Also, these findings imply that Tpk2 activity antagonizes Tpk1 and Tpk3 action, as has been suggested by other studies on the control of iron uptake and pseudohyphal growth [61, 68, 69].

### Heat shock gene transcript levels are reduced when Tpk3 is the only CS

To learn more about the strong repressing activity of Tpk3 upon Hsf1, when Tpk1 and Tpk2 are absent, we studied the levels of several stress genes within the context of their natural promoters. As shown in Additional file 2: Figure S1, expression of the heat shock genes *HSP104*, *HSP82*, *SSA3*, *HSP26*, and *HSP12* at 25 °C was reduced in the *tpk1Δ tpk2Δ* mutant relative to the WT strain. This result is consistent with the low level of induced thermotolerance displayed by the *tpk1Δ tpk2*Δ mutant (Additional file 1: Table S3). Transformation of *tpk1Δ tpk2Δ* cells with *TPK2* in a CEN plasmid did not complement fully the HSE-dependent gene expression at WT levels (data not shown), most likely because *TPK2* gene copy number per cell was not 1, but 2.8 copies/cell. Transformation of the *tpk1Δ tpk2Δ* cells with *TPK2* in a 2 μ plasmid was toxic to the cell, explaining the surprisingly low copy number in the surviving cells (1.7 copies/cell).

### Tpk2 antagonizes the activity of Tpk1

To further test the hypothesis that the loss of *TPK2* in the *tpk2Δ tpk3Δ* double mutant causes repression of HSE-dependent gene expression, the *TPK2* gene was returned to the *tpk2Δ tpk3Δ* double mutant using the *delitto perfetto* technique (see Methods) [30, 79], restoring the native copy number of the gene. This modification (*tpk2Δ::TPK2 tpk3Δ*) returned HSE-dependent expression to WT levels (Fig. 5), supporting the idea that Tpk2 antagonizes the activity of Tpk1 on HSE-dependent expression.

### Catalytic activity of PKA in extracts from *TPK* mutants

We hypothesized that antagonism between Tpk2 and the other CS (Tpk1 and Tpk3) was due to drastic changes in the total PKA activity of the cell. Accordingly, we could expect that the total PKA activity in *TPK2* mutants (*tpk2Δ*, *tpk1Δ tpk2Δ*, and *tpk2Δ tpk3Δ*) would be high, whereas in the WT, *tpk1Δ*, *tpk3Δ*, and *tpk1Δ tpk3Δ* mutants the PKA activity would be low. After addition of cAMP, PKA activity in extracts from mutants *tpk1Δ*, *tpk3Δ*, and *tpk1Δ tpk3Δ* was similar to the WT (Additional file 2: Figure S2). On the contrary, cAMP-dependent PKA activity decreased in *tpk2Δ*, *tpk1Δ tpk2Δ*, and *tpk3Δ* mutants. These results indicate that HSE-dependent expression is not a simple reflection of the overall PKA activity within the cell. Alternatively, one could also propose that deletion of a given *TPK* gene reduced the PKA activity in the cell in a proportional manner to its abundance in the WT. It is established that during exponential growth in liquid cultures yeasts contain a large proportion of Tpk1, followed by Tpk2, and Tpk3 being the one with the lowest abundance [88]. Thus, elimination of *TPK1* and/or *TPK2* should diminish dramatically the PKA activity in the cell. This was the case for *TPK2* deletions but not for *TPK1* deletions (Additional file 2: Figure S2), indicating again that deletion of a given *TPK* gene does not influence arithmetically the overall PKA activity in the cell. Therefore, dynamic mechanisms seem to define the final PKA activity in the WT and in a given *TPK* mutant (interactions between CS, compartmentalization, stability, etc.).

### Ssa1 and Ssa2 mediate the inhibition of HSE-dependent gene expression

**Fig. 6:**
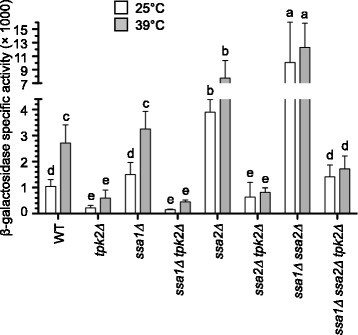
A role for *SSA1* and *SSA2* in the repression of HSE-dependent gene expression. Strains transformed with reporter plasmid pRY016 were grown in SD medium at 25 °C until mid-exponential phase and treated at different temperatures as described in Methods section. Data shown represent the average and standard deviation of at least three independent experiments. β-galactosidase specific activities are reported as in Fig. 2. Bars that do not share at least a common letter differ significantly (*P* < 0.05). Strains assayed were: WT (W303-1a), *tpk2Δ* (KG604), *ssa1Δ* (S001), *ssa1Δ tpk2Δ* (S002), *ssa2Δ* (SL622), *ssa2Δ tpk2Δ* (SL623), *ssa1Δ ssa2Δ* (SL625), *ssa1Δ ssa2Δ tpk2Δ* (SL708)

Our initial computational model assumed that the regulation of Hsf1/Skn7 by the CS was direct. However, under this design, predicted and experimental HSE-activities for several PKA-RN mutants gave contrasting results. Complete agreement between experimental and computational data was not achieved until a negative regulator was placed as an intermediary between the CS and Hsf1/Skn7 (see Fig. 1 and the following subsection). This idea was in accordance with previous findings demonstrating that the CS's do not interact directly with Hsf1 [20]. Therefore, we considered Hsp70 chaperones as putative intermediate inhibitors, because they are well-known negative regulators of Hsf1. Yeast mutants with decreased Hsp70 levels increase the expression of Hsps, enhance thermotolerance, and grow slowly. Additionally, these phenotypes are suppressed by a mutation in *HSF1* that decreases its DNA binding affinity [13, 34, 92]. These observations and others from both mammals and yeast reinforce a model that includes an auto-regulatory loop in which Hsp70 represses Hsf1 activity [4, 12, 94]. Moreover, Ssa1 positively controls the PKA-RN by stabilizing Cdc25 at optimal temperatures [26] and, under stress, the Cdc25-Hsp70 complex dissociates leading to a loss of Cdc25 levels and a decrease in the activity of the PKA pathway [26]. Our experiments revealed that deletion of *SSA2* increased HSE-dependent gene expression (Fig. 6). Deletion of *SSA1* did not affect HSE-dependent gene expression significantly, indicating that *SSA2* suffices for maintaining WT activity. Deletion of both *SSA1* and *SSA2* largely increased the reporter activity, uncovering the contribution of both Hsp70 genes as repressors of HSE-dependent gene expression in WT cells. Interestingly, deletion of *SSA1* or *SSA2* in a *tpk2Δ* background did not suppress the strong repression of HSE-dependent gene expression characteristic of the *tpk2Δ* single mutant (Fig. 6). However, the phenotype of the *tpk2Δ* mutant was suppressed in the triple mutant *ssa1Δ ssa2Δ tpk2Δ*, as its HSE-dependent expression was higher than in *tpk2Δ* cells (at 25 °C and 39 °C), similar to that of the *ssa1Δ* and the WT at 25 °C, and lower compared to *ssa1Δ* and the WT at 39 °C. These results implicated Ssa1 and Ssa2 not only as mediators of the strong repression of HSE-dependent gene expression, but also suggest the existence of an additional repressor of Hsf1/Skn7, active in the absence of Tpk2.

### The dynamical model of the PKA-RN revealed an additional negative regulator of Hsf1

To thoroughly understand the implications of our observations we constructed a discrete dynamical model of the PKA-RN based both on our results and in the literature [4, 10, 19, 20, 23, 26, 60, 61, 66, 68, 69, 76, 78, 84, 89, 94]. As described in the Methods section, we have used an extension of a synchronous discrete modeling framework, as this type of modeling is known to accurately predict the behavior of several biological networks. One of the advantages of the discrete framework is that it only requires knowledge about the regulatory nature of the interactions involved, contrary to reaction-kinetic differential equations that require the precise values for all the kinetic parameters and cooperativity exponents of the network elements. For a detailed review of the advantages and disadvantages of discrete and Boolean models compared to other frameworks consult [1, 38, 71, 90].

Briefly, our model consists on *N* elements {*σ*_*1*_*, σ*_*2*_*,…, σ*_*N*_} whose dynamical states take integer values ranging from 0 to *m*_*i*_, where *m*_*i*_ is the maximum level of activity (or level of expression) for element *σ*_*i*_. Usually only two levels of activity are implemented: either the node is active (*σ*_*i*_ = 1) or it is inactive (*σ*_*i*_ = 0). However, often the functionality of a given node depends on whether it has a low, mild or high level of activity [9] and the binary description is not enough. This is the case here, as our experiments indicate that some nodes of the TPK-RN require distinction of up to six levels of activity (see Additional file 3: Sections 3 and 4 in the Supplementary Information). Additionally, as currently there is no information about the time scales implicated in the dynamics of the PKA-RN elements, for graphing we used a synchronous updating scheme (see Methods).

For each network (we will consider WT, *tpk1∆*, etc., as different networks) we sampled about 10 % of the complete set of initial conditions (which consists of more than 4 billion points) looking for steady states of expression (attractors) (see Additional file 4: Text S1). As several initial conditions may fall into the same attractor, we define the size of the basin of attraction *B*_*k*_ as the number of initial conditions that fall into attractor *K*. Our extension of this traditional modeling framework consists in two simple modifications. First we averaged the level of expression for each element over a time window whose length equaled the attractor period. This gave us a single continuous value *A*_*ik*_ for each element *σ*_*i*_ in the *k*^*th*^ attractor. Then, to better represent the experimental measurements from liquid batch cultures where a single average expression level is obtained, we averaged the quantities *A*_*ik*_ over all the attractors of the network, weighted by the size of the corresponding basin of attraction (see Methods). Thus, contrary to other studies [9, 46, 52], we avoided discarding any attractor reached by the network deeming it as “non-biologically relevant”.

From now on, we will refer to this extension as the Windowed Discrete Model (WDM). This statistical treatment of data is supported by experimental studies showing that individual yeast cells in batch cultures exhibit different cell cycle phases, physiological states, and gene expression patterns that result in a heterogeneous population [23, 40, 53]. With this procedure, we were able to make a direct and semi-quantitative comparison between the model predictions and the experimental measurements. The WT interaction network considered is shown in Fig. 1 and the logic rules governing the dynamics of the system are presented on the Supplementary Information (see Additional file 3: Section 3).

The modeled PKA-RN starts with the Cdc25-Ras branch. Cdc25 abundance and function are dependant on the activity of the Hsp70 chaperones (Ssa1 and Ssa2) [26]. Under optimal temperature and nutrients conditions, Cdc25 acts as the positive regulator of Ras2 activity [6, 22], which in turn activates Cyr1 (adenylate cyclase) [43]. The product of Cyr1, cAMP, negatively regulates the inhibition imposed by Bcy1 upon the CS Tpk1, Tpk2, and Tpk3 [84]. The CS were modeled as a module showing antagonism, as our results (Figs. 1 and 5) and those from others have suggested [61, 68, 69]. We propose that Tpk2 activity inhibits the activation of Ssa1 and Ssa2 by the Tpk1 and Tpk3 subunits. The implication for this interaction is that, in a WT background where the three CS are active, only the activity of Tpk2 is effective in activating Ssa1 and Ssa2 chaperones. The mechanistic basis for this antagonism remains to be studied. A systematic study of yeast kinases, made *in vitro*, showed that some CS have as substrates other CS. In particular, Tpk1 phosphorylates Tpk2 and Tpk3; Tpk3 phosphorylates Tpk2; and Tpk2 phosphorylates Tpk3 [65]. It remains to be seen whether the antagonism between the CS is caused by their mutual phosphorylation or whether it occurs via other indirect mechanisms.

As mentioned above (Fig. 6), the inhibition of the HSE-dependent expression by the PKA-RN requires the activation, by the TPKs, of an inhibitor of Hsf1 and Skn7. Ssa1 and Ssa2 (Hsp70 proteins) were introduced into the model as repressors of the HSE-dependent expression [4, 78] (Fig. 6). Moreover, based on the expression levels of the triple mutant *ssa1Δ ssa2Δ tpk2Δ* (Fig. 6), we included a third repressor of Hsf1/Skn7 that gets activated exclusively when Tpk1 and Tpk3 become the only CS (i.e., when Tpk2 is absent or at minimum levels). We believe that a very plausible candidate for such a repressor could be Hsp90, given that Hsp90 binds to Hsf1 [59, 96] and its deletion increases HSE-dependent expression [16]. Moreover, Tpk1 and Tpk3 phosphorylate Hsp82 (Hsp90) *in vitro* [65]; although the functional significance of this phosphorylation is unknown. It is plausible that the binding of Hsp90 to Hsf1 could be enhanced upon phosphorylation by Tpk1 or Tpk3, but this needs to be addressed experimentally. Similarly, Tpk1 and Tpk3 could enhance the repression of Hsf1 by other members of the Hsp70 family, such as Ssb1 or Ssb2, as it is known that Ssb1 and Ssb2 form complexes with Hsf1 and deletion of their genes also increases HSE-dependent expression [4]. However, more work is needed to identify the third repressor that is unleashed in the absence of Tpk2. In any case, it is important to stress that only by including the three repressors (Ssa1, Ssa2, and the putative third repressor), the experimental measurements could be reproduced by the model.

### Quantitative comparison between theoretical and experimental results corroborates the proposed regulatory interactions

**Fig. 7:**
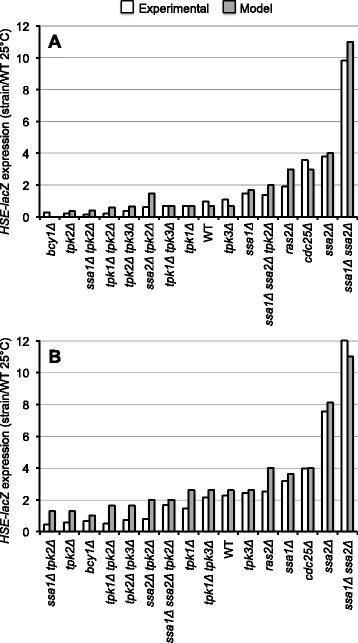
Comparison between experimental data and predictions by the WDM. Comparison between experimental and theoretical measurements for *HSE-lacZ* activity. Values are given as average ratios between strain expression and WT expression at 25 °C, making the average expression ratio of the WT strain at 25 °C equal to one. Theoretical and experimental values show similar quantitative behavior across strains. Moreover, since the theoretical values are no longer discrete, subtle differences occurring experimentally are reproduced also by the model. **a** Ratios at 25 °C, **b** ratios after a heat shock at 39 °C

To validate the simulations of our model, we compared the HSE-dependent expression results obtained computationally and those obtained experimentally in a number of mutant strains. Population measurements were reported as the ratio (strain expression level)/(WT expression level) and are presented in Additional file 1: Table S4. Figure 7 shows that the results obtained with the WDM closely resembled the experimental results obtained for all strains. The great concordance between theory and experiment suggests that the novel interactions proposed here for the PKA-RN are very likely true. Additionally, we also implemented several asynchronous updating schemes and the results that we obtained for the population expression level were almost identical regardless of the synchronicity or asynchronicity of the updating scheme (Additional file 2: Figure S3). This feature is quite relevant because, for a particular network (single-cell level) the use of asynchronous updating can significantly change the dynamical attractors of the network [15, 36] to the point that random asynchronous updating has been called inadequate in some scenarios [15]. We present the structure of the attractor landscape for the 25 °C WT network using synchronous updating (Additional file 2: Figures S5 and S6). As this example shows, different basins of attraction varying in size can be visualized. The WDM takes this fact into account to simulate subgroups of cells that might correspond to the different basins of attraction.

**Fig. 8:**
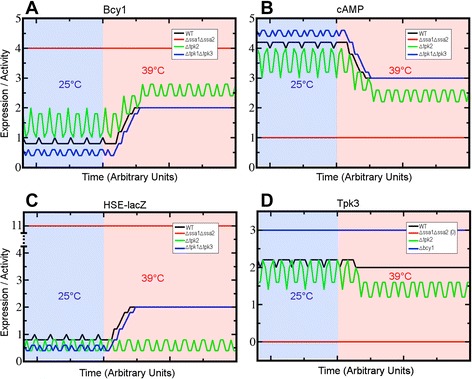
Single-cell predictions of the temporal dynamics for Bcy1, cAMP, *HSE-lacZ,* and Tpk3 in WT and three mutants. Temporal dynamics for four selected nodes: Bcy1 (**a**), cAMP (**b**), *HSE-lacZ* (**c**), and Tpk3 (**d**). Line colors correspond to different strains. Simulations were made starting from a random initial condition for each strain. Expression and time are given in arbitrary units. Background color represents the temperature of the culture: 25 °C (blue) and 39 °C (pink)

In addition to the population measurements, we present simulations for the temporal dynamics of Bcy1, cAMP, *HSE-lacZ,* and Tpk3 that, presumably, could be valid for single-cell measurements (Fig. 8). Each curve represents a simulation corresponding to a different strain (WT, *ssa1Δ ssa2Δ*, *tpk2Δ*, and *tpk1Δ tpk3Δ*) starting from a random initial condition. At time *t*_0,_ an increase in the temperature was simulated by turning on the heat shock node. In the absence of Ssa1 and Ssa2 (Fig. 8, red lines), the levels of *HSE-lacZ* activity and Bcy1 increased dramatically, while the levels of cAMP and Tpk3 were very low. In the absence of Ssa1 and Ssa2, the dynamics of Tpk1 and Tpk2 were identical to Tpk3 (data not shown). The particular temporal dynamics observed in these simulations (oscillatory behavior, spikes, etc.) remain to be experimentally confirmed through the use of single-cell measurements. Nonetheless the predictions reported in Fig. 8 fit well the experimental data showing that *ssa1Δ ssa2Δ* mutants are constitutively resistant to high temperature and display elevated production of Hsp’s and slow growth rates [34]. Deletion of *TPK2* also decreased the expression of *HSE-lacZ* with respect to the WT, but more conspicuously at 39 °C than at 25 °C, consistent with the lower induced thermotolerance level in this mutant (Additional file 1: Table S3).

## Conclusions

Our results clarified the control of Hsf1 and Skn7 by the PKA-RN, demonstrating that in the W303 strain, PKA represses Hsf1 and also Skn7 via Ssa1, Ssa2 and a third unidentified repressor. No single component of the PKA-RN could be used to predict accurately the experimental levels of downstream targets (i.e., HSE-dependent gene expression) in all the situations studied. Instead, modeling of the PKA-RN showed that the observed experimental dynamics arose from the complex interactions of the network making it necessary to analyze the system as a whole. It remains to be unveiled the exact molecular mechanisms by which the PKA CS inhibit Hsf1 and Skn7 activities. Our results indicate that such mechanism must exist and it depends on Ssa1, Ssa2, and at least a third unidentified repressor. During PKA-RN controlled processes, such as pseudohyphal growth and iron uptake, the PKA CS display similar antagonistic relationships to those observed during the control of HSE-dependent gene expression. Tpk2, but not Tpk1 or Tpk3, is required for the induction of pseudohyphal growth and for the inhibition of genes involved in iron uptake [61, 68, 69]. Additionally, the fact that various updating and averaging schemes produced essentially the same results is quite interesting (Additional file 2: Figure S3), as this means that the WDM really captured the population average in batch cultures regardless of the specific updating scheme. To our knowledge, this is the first model with this property. Thus, with simple modifications, it can pave the way for the analysis of many other cellular responses at the population level apart from the PKA-RN.

## Methods

### Media and growth conditions

Yeast cells were grown at 25 °C in media prepared as previously described in [23], unless otherwise indicated. Duplication times of the strains were also calculated as described in [23].

### Strains and plasmids

All strains employed are described in Additional file 1: Table S5. Strains with identical auxotrophies and other genetic markers were used in all experiments to avoid phenotypic differences due to marker effects. Integrative gene-disruption cassette *kanMX6* [31, 54] was used to generate strains with full disruptions in *CDC25*, *RAS2*, *BCY1*, *SKN7*, *TPK1*, *TPK2*, *TPK3*, *SSA1*, *SSA2*, or in the C-terminal transcriptional activation domain of Hsf1 (*hsf1-ΔCTA*) in strains W303-1a, W303-6B, JF3100 or JF3000. Transformants were selected on YPDA medium plus 300 μg ml^−1^ of geneticin. The correct insertion of the cassette on each mutant was verified by PCR.

Reporter plasmid pRY016 (*HSE-CYC1-lacZ*) was generated by annealing oligonucleotides, HSEA and HSEB (Additional file 1: Table S6). Self-ligation products were separated by electrophoresis in agarose gels and the band corresponding to the dimer was eluted from the gel. The protruding ends were filled-in using the Klenow enzyme and ligated to *Bgl*II adapters for cloning into the *Bgl*II site of plasmid pLGΔBS. Plasmid pLGΔBS is a high copy number 2 μ vector with a *CYC1-lacZ* fusion, lacking *UAS* and derived from the pLG669Z [35]. The resulting plasmid, pRY016, contained nine 5 bp units of the HSE consensus sequence nGAAn [21] arranged in both, sense and anti-sense orientations. To assess the dependency of β-galactosidase activity on the HSEs present in pRY016, the W303 WT strain and several PKA-RN mutants, we transformed them with a pRY016-derivative plasmid expressing the same *CYC1-lacZ* gene fusion but lacking HSEs. The β-galactosidase activity at 25 °C or after a heat shock at 39 °C in all strains was negligible (10 to 30 units) indicating that enzyme expression using pRY016 is indeed HSE-dependent. To investigate whether the levels of β-galactosidase activity in the strains used in this work were influenced by differences in the copy number of the reporter plasmid, pRY016 copy number was measured in all strains. The correlation coefficient of pRY016 plasmid copy number and β-galactosidase activity (Pearson´s = 0.35341449) was not significant (P = 0.0765) (Additional file 2: Figure S4). Therefore, the activity of the pRY016 reporter seems to be a reflection of actual changes in HSE-dependent gene expression influenced by the mutations and not by plasmid copy number.

### Stress tolerance assays

Basal thermotolerance was measured as described [23]. To determine induced-thermotolerance, cultures were exposed at 39 °C for 60 min prior to a 50 °C heat shock for 20 min. For both basal and induced thermotolerance, aliquots of each culture were taken before and immediately after the 50 °C treatment, and dilutions were plated on solid YPD to measure cell viability by colony counting. Thermotolerance levels are expressed as the percentage of the number of colonies after a heat shock divided by the number of colonies in the control sample.

### Biochemical analysis

β-galactosidase assays were performed from exponentially grown cultures (OD_600_ between 0.4-0.6) in SD media as described [70]. β-galactosidase specific activity is expressed as nmol of hydrolyzed ONPG min^−1^ mg^−1^ protein. To measure the response to a heat shock, cells were treated at 39 °C for 1 or 2 h as described [60, 77].

### Genetic techniques and nucleic acid manipulations

DNA manipulations and genetic techniques were performed according to Sambrook, Fritsch & Maniatis [72] and Guthrie & Fink [32], respectively. DNA sequencing was performed at the Unit for DNA Synthesis and Sequencing of the *Instituto de Biotecnología*. Yeast transformation was performed following the method presented in [27].

### RNA isolation and northern blot analyses

Total RNA was isolated from exponentially-growing cells in SD medium at 25 °C by the method of Collart and Oliviero [11]. Aliquots (10 μg) of total RNA were separated by electrophoresis on 1 · 2 % (w/v) agarose gels containing formaldehyde, transferred to IMMOBILON-NY+ membranes (Millipore) and hybridized as described by the manufacturer. The 3 · 0 kbp *Bam*HI fragment of clone pYSGal104 (courtesy of Dr. Susan Lindquist) was used as DNA probe to detect *HSP104* transcripts. Gene-specific DNA probes for *HSP82*, *SSA3*, *HSP26*, *HSP12*, and *ACT1* were amplified by PCR. Primer pairs used during PCR were: FSHSP82 and RSHSP82 for HSP82; fc-ssa3 and rc-ssa3 for *SSA3*; HSP26-F and HSP26-R for *HSP26*; HSP12-F and HSP12-R for *HSP12*; ACT1-1 and ACT1-2 for *ACT1* (Additional file 1: Table S5). Estimation of band intensities of autoradiograms was performed by image analysis with NIH Image 1.62 software. Data was normalized to account for differences between samples in actual total-RNA loading.

### Estimation of plasmid copy number in yeast strains

Strains were grown under similar conditions to those of β-galactosidase assays. Southern blots of total genomic DNA were digested with *Pst*I and hybridized to the 340 bp *Pst*I–*Sca*I fragment of plasmid pRS3 encoding the N-terminus of Ura3. Copy number of pRY016 was estimated as the ratio of plasmid/genome *URA3* signal. Band intensities of autoradiograms were measured with NIH Image 1.62 software.

### Complementation of strain *tpk2Δ tpk3Δ* by reintroduction of *TPK2* gene

Complementation of strain KS590 (*tpk2Δ::loxP tpk3Δ::loxP*), was carried by a protocol based on the *delitto perfetto* technique [30, 79]. First, the *URA3* gene was amplified by PCR using plasmid pRS306 [74] as template. Oligonucleotides FTPK2-URA3 and RTPK2URA3 contained 40 bp of sequence flanking each side of the *tpk2Δ::loxP* chromosomal deletion followed by *URA3* flanking sequences. The PCR product obtained was transformed by homologous recombination into strain KS590 to get strain KS590-URA3 (*tpk2Δ::URA3 tpk3Δ::loxP*). Finally, *URA3* gene in strain KS590-URA3 was evicted by interchanging *TPK2* using the product of a PCR reaction that amplified *TPK2* with TPK2-Ucl and TPK2-Lcl oligonucleotides. The resulting strain, KS590-URA3-TPK2 (*tpk2Δ::TPK2 tpk3Δ::loxP*), was selected by resistance to FOA at 1 mg/ml [7]. *TPK2* gene was re-amplified by PCR from KS590-URA3-TPK2 to select candidates with the correct DNA sequence.

### Measurement of cAMP-dependent PKA activity

Cells were cultured in 50 ml of SD medium at an OD_600_ of 0.4. After centrifugation, the pellet was washed in cold miliQ water and centrifuged once more. The washed pellet was frozen in liquid N_2_. Cells were broken with a mortar and pestle under liquid N_2_ and resuspended in extraction buffer (50 mM Tris pH 7.4, 20 mM β-mercaptoetanol, 0.5 mM PMSF, and 4 mg/ml COMPLETE™, a mixture of protease inhibitors [Roche, cat. no. 11697498001]). The total protein extract was centrifuged twice and the final supernatant was saved. Total protein was estimated by the Bradford method [5]. Finally, aliquots containing 4 mg of protein were assayed for PKA activity according to the Pep Tag® protocol (PROMEGA, cat. no. V5340). Activity was assayed in the presence or absence of 1 μM cAMP. Only extracts from a *bcy1Δ* mutant, used as a control, showed PKA activity in the absence of exogenous cAMP. WT and *TPK* mutants showed total dependency on cAMP for PKA activity.

### Statistical analysis

All experiments were conducted at least three times. Comparisons between given pairs were analyzed using the two-tailed T Student test. Pairs of data were considered significantly different only when P < 0.05. For multiple comparisons, data were subjected to analysis of variance (ANOVA), and differences between the means were compared by Tukey (one-way) or Bonferroni (two-way) post-tests. Treatments were considered as statistically different to the control when P ≤ 0.05. Prism 5.0 software package was used.

### The Windowed Discrete Model

The dynamic model consists of a network of 15 nodes representing the regulatory interactions of the PKA-RN shown in Fig. 1. Each node acquires a set of discrete values that represent the level of expression of the corresponding network element. Like many other discrete models available [2, 9, 52], ours focuses on the functional state of expression (or activation) of the network components, rather than on their exact concentrations. These functional states of expression are modeled trough discrete variables that take a finite number of values. To capture the various levels of expression observed experimentally for the *HSE-CYC1-lacZ* reporter, the number of functional states for each node was determined by the maximum number of statistically-significant different groups of ß-galactosidase activity displayed experimentally by the whole panel of WT and PKA-RN mutant strains during exponential phase (Figs. 2, 4, 5, and 6). Our final model consisted on two binary nodes, one ternary, four four-valued, one five-valued and seven six-valued elements. This gives a total of Ω = 4,299,816,960 possible dynamical states for the network.

As in the standard Kauffman model [44], the network dynamics is given by the simultaneous updating of all the network elements according to the equation 1:

$$ {\sigma}_n\left(t+1\right)={F}_n\left({\sigma}_n^1(t),{\sigma}_n^2(t),\cdots, {\sigma}_n^{k_n}(t)\ \right) $$

where *σ*_*n*_(*t*) represents the state of the *n*^th^ element of the network at time *t*, $\left\{{\sigma}_n^1,{\sigma}_n^2,\cdots, {\sigma}_n^{k_n}\ \right\}$ are the *k*_*n*_ regulators of *σ*_*n*_ and *F*_*n*_(⋅) is a discrete function (also known as a logical rule) that determines the state of *σ*_*n*_ in terms of the states of its regulators. This function *F*_*n*_(⋅) is constructed according to experimental evidence regarding the regulatory interactions (activator or inhibitor) for each node. All the functions *F*_*n*_(⋅) for the PKA-RN are listed in the Supplementary Information (see Additional file 3: Section 3).

Since each variable acquires a finite number of states, there are also a finite number of possible dynamical configurations for the entire network, ranging from the configuration in which all the nodes are inactive, to the configuration in which all the nodes have reached their maximum values of activation. Once the dynamics from any of these possible configurations starts, successive iterations of Eq. (1) will make the network traverse through a series of states until a periodic pattern of activity is reached. This periodic activity is known as an attractor, and for each network several attractors might exist. Which attractor the network falls into depends on the initial condition the network starts from. The set of all the initial conditions that eventually fall into the same attractor is known as the *basin of attraction*. It has been previously shown that attractors represent the stable patterns of activity of the real biological system, and the basins of attraction correspond to the different ways to reach these stable states [41]. Nonetheless, a direct comparison of an attractor to HSE-dependent expression levels might not be so straightforward, as attractors may be often composed by several states (cyclic attractors) and experimental gene expression is often presented as a single value (e.g. β-galactosidase activity). Moreover, experimental measures of gene expression are commonly taken from a population of cells, which makes the final measurement an average. For this reason we have developed the WDM, where the state of each element of the network is represented by its average expression over a time window. In our model, the length of the window (L) for each realization corresponds to the length of the attractor reached. Although other sizes can be used with similar results, sizes bigger than the length of the attractor are not convenient as they tend to flatten the dynamics.

Additionally, since a network can have more than one attractor, we have calculated a weighted average using the entire set of attractors (N) for each network. Thus, we define the average expression level of *σ*_*n*_ as:

$$ {\sigma}_n = {\displaystyle \sum_{a=1}^N}{\omega}_a{\left(\frac{{\displaystyle {\sum}_{\tau =1}^{L_a}}{\sigma}_n\left({t}_0+\tau \right)}{L_a}\right)}_a $$

where *N* is the number of different attractors and the external sum is carried out over all the attractors. The parameter *ω*_*a*_ is the fractional size of the basin of attraction of the *a*^*th*^ attractor (∑_*a* = 1_^*Ν*^*ω*_*a*_ = 1). The internal sum is carried out over the *L*_*a*_ states of the *a*^*th*^ attractor, and *t*_0_ is a transient time long enough as to guarantee that the system has reached the attractor.

This simple modification, apart from allowing an easier comparison between the model and experimental data, resembles the way in which experimental data is gathered for gene expression in batch cultures, where traditionally measurements of the level of expression represent the population average, as cells in the population are at different stages of a stable pattern of gene expression (unless synchronization is enforced).

To simulate deletions in our numerical experiments, we just kept the value of the deleted node equal to zero throughout the dynamics, which represents the complete absence of that node.

Elevated temperatures increase the number of targets of the Hsp70s, reducing their positive interaction over Cdc25 [26] and the inhibition of Hsf1 [4, 89]. Therefore, heat shock (HS) was introduced into the model as a node of the PKA-RN that affects the functional state of Ssa1 and Ssa2. Its logical function corresponds to a positive auto regulation (see Additional file 3: Supplementary Information, Section 3). This means that whenever this node is active (which corresponds to the 39 °C condition), it remained active all the time. By contrast, the 25 °C condition is represented by inactivating the HS node and keeping it inactivated throughout the simulation time.

## Availability of supporting data

All supporting data are included as additional files.

## Additional files

Additional file 1:
**Table S1. **Growth and thermotolerance of WT yeast strains and mutants deleted in *CDC25*, *RAS2* or *BCY1*. **Table S2.** Contribution of Hsf1 and Skn7 to the elevated thermotolerance and slow growth rate of the *cdc25Δ* mutant. **Table S3.** Growth and thermotolerance of WT or mutant yeast strains deleted in *TPK1*, *TPK2* or *TPK3*. **Table S4.** Relative β-galactosidase activity values for all strains used in this work. **Table S5.** Strains used in this study. Additional file 2:
**Figure S1. **Correlation analysis between pRY016 plasmid copy number and HSE-dependent gene expression. **Figure S2.** Levels of *HSP* transcripts in WT and *tpk2Δ tpk3Δ* mutant. **Figure S3.** Comparison of different updating schemes. **Figure S4.** cAMP-dependent PKA activity in extracts of WT and *TPK* deletion mutants. **Figure S5.** Reduced network used to compute the structure of the attractor landscape. **Figure S6.** Structure of the attractor landscape displayed by the WT reduced network shown in Figure S5C. Additional file 3:
**Section 1.** Synchronous and Asynchronous Updating Schemes. **Section 2.** Basins of attraction. **Section 3.** Assumptions and logical functions for the Windowed Discrete Model. **Section 4.** Logic Tables and discrete regulatory functions in the WT at 25 °C. Additional file 4:
**Text S1. **The attractors for several simulated strains are presented here.

## Abbreviations

PKA: cAMP-dependent protein kinase
PKA-RN: cAMP-dependent protein kinase regulatory network
CS: Catalytic subunits of PKA

## References

[1] Albert R (2004). Boolean modeling of genetic regulatory networks. Complex Networks Lecture Notes in Physiscs.

[2] Albert R, Othmer HG (2003). The topology of the regulatory interactions predicts the expression pattern of the *Drosophila* segment polarity genes. J Theor Biol.

[3] Amoros M, Estruch F (2001). Hsf1p and Msn2/4p cooperate in the expression of *Saccharomyces cerevisiae* genes *HSP26* and *HSP104* in a gene- and stress type-dependent manner. Mol Microbiol.

[4] Bonner JJ, Carlson T, Fackenthal DL, Paddock D, Storey K, Lea K (2000). Complex regulation of the yeast heat shock transcription factor. Mol Biol Cell.

[5] Bradford M (1976). A rapid and sensitive method for the quantitation of microgram quantities of protein utilizing the principle of protein-dye binding. Anal Biochem.

[6] Broek D, Toda T, Michaeli T, Levin L, Birchmeier C, Zoller M (1987). The *S. cerevisiae CDC25* gene product regulates the RAS/adenylate cyclase pathway. Cell.

[7] Burke D, Dawson D, Stearns T (2000). Methods in Yeast Genetics: a Cold Spring Harbor Laboratory Manual.

[8] Cazzaniga P, Pescini D, Besozi D, Mauri G, Colombo S, Martegani E (2008). Modeling and stochastic simulation of the Ras/cAMP/PKA pathway in the yeast *Saccharomyces cerevisiae* evidences a key regulatory function for intracellular guanine nucleotides pools. J Biotechnol.

[9] Chaos A, Aldana M, Espinosa-Soto C, García Ponce de León B, Garay Arroyo A, Alvarez-Buylla ER (2006). From Genes to Flower Pattern and Evolution: Dynamic Models of Gene Regulatory Networks. J Plant Growth Regul.

[10] Charizanis C, Juhnke H, Krems B, Entian KD (1999). The oxidative stress response mediated via Pos9/Skn7 is negatively regulated by the Ras/PKA pathway in *Saccharomyces cerevisiae*. Mol Gen Genet.

[11] Collart MA, Oliviero S: Preparation of yeast RNA. In *Current Protocols in Molecular Biology*. Edited by Ausubel FM, Brent R, Kingston RE, Moore DD, Seidman JG, Smith JA, Struhl K. New York, NY; Wiley; 1993:pp. 13.12. Vol. 2.

[12] Craig EA, Gross CA (1991). Is hsp70 the cellular thermometer?. Trends Biochem Sci.

[13] Craig EA, Jacobsen K (1984). Mutations of the heat inducible 70 kilodalton genes of yeast confer temperature sensitive growth. Cell.

[14] Dhar R, Nieto A, Koller R, Defeo-Jones D, Scolnick EM (1984). Nucleotide sequence of two rasH related-genes isolated from the yeast *Saccharomyces cerevisiae*. Nucleic Acids Res.

[15] Di Paolo EA (2001). Rhythmic and non-rhythmic attractors in asynchronous random Boolean networks. BioSystems.

[16] Duina AA, Kalton HM, Gaber RF (1998). Requirement for Hsp90 and a CyP-40-type cyclophilin in negative regulation of the heat shock response. J Biol Chem.

[17] Engerlberg D, Zandi E, Parker CS, Karin M (1994). The yeast and mammalian Ras pathways control transcription of heat shock genes independently of heat shock transcription factor. Mol Cell Biol.

[18] Erkina TY, Tschetter PA, Erkine AM (2008). Different requirements of the SWI/SNF complex for robust nucleosome displacement at promoters of heat shock factor and Msn2- and Msn4-regulated heat shock genes. Mol Cell Biol.

[19] Estruch F (2000). Stress-controlled transcription factors, stress-induced genes and stress tolerance in budding yeast. FEMS Microbiol Rev.

[20] Ferguson SB, Anderson ES, Harshaw RB, Thate T, Craig NL, Nelson HCM (2005). Protein kinase A regulates constitutive expression of small heat-shock genes in an Msn2/4p-independent and Hsf1p-dependent manner in *Saccharomyces cerevisiae*. Genetics.

[21] Fernandes M, Xiao H, Lis JT (1994). Fine structure analyses of the *Drosophila* and *Saccharomyces* heat shock factor-heat shock element interactions. Nucleic Acids Res.

[22] Field J, Broek D, Kataoka T, Wigler M (1987). Guanine nucleotide activation of, and competition between, RAS proteins from *Saccharomyces cerevisiae*. Mol Cell Biol.

[23] Folch-Mallol JL, Martínez LM, Casas SJ, Yang R, Martínez-Anaya C, López L (2004). New roles for *CDC25* in growth control, galactose regulation and cellular differentiation in *Saccharomyces cerevisiae*. Microbiology.

[24] Garmendia-Torres C, Goldbeter A, Jacquet M (2007). Nucleocytoplasmic oscillations of the transcription factor Msn2: evidence for periodic PKA activation. Curr Biol.

[25] Garrett S, Broach J (1989). Loss of Ras activity in *Saccharomyces cerevisiae* is suppressed by disruptions of a new kinase gene, *YAKI*, whose product may act downstream of the cAMP-dependent protein kinase. Genes Dev.

[26] Geymonat M, Wang L, Garreau H, Jacquet M (1998). Ssa1p chaperone interacts with the guanine nucleotide exchange factor of ras Cdc25p and controls the cAMP pathway in *Saccharomyces cerevisiae*. Mol Microbiol.

[27] Gietz RD, Woods RA (2002). Transformation of yeast by lithium acetate/single-stranded carrier DNA/polyethylene glycol method. Methods Enzymol.

[28] Gonzalez K, Kayikçi Ö, Schaeffer DG, Magwene PM (2013). Modeling mutant phenotypes and oscillatory dynamics in the *Saccharomyces cerevisiae* cAMP-PKA pathway. BMC Systems Biology.

[29] Gonze D, Jacquet M, Goldbeter A (2008). Stochastic modelling of nucleocytoplasmic oscillations of the transcription factor Msn2 in yeast. J R Soc Interface.

[30] Gray M, Piccirillo S, Honigberg SM (2005). Two-step method for constructing unmarked insertions, deletions and allele substitutions in the yeast genome. FEMS Microbiol Lett.

[31] Guldener U, Heck S, Fielder T, Beinhauer J, Hegemann JH (1996). A new efficient gene disruption cassette for repeated use in budding yeast. Nucleic Acids Res.

[32] Guthrie C, Fink GR (1991). Guide to Yeast Genetics and Molecular Biology.

[33] Hahn JS, Hu Z, Thiele DJ, Iyer VR (2004). Genome-wide analysis of the biology of stress responses through heat shock transcription factor. Mol Cell Biol.

[34] Halladay JT, Craig EA (1995). A heat shock transcription factor with reduced activity supresses a yeast *HSP70* mutant. Mol Cell Biol.

[35] Harshman KD, Moye-Rowley WS, Parker CS (1988). Transcriptional activation by the SV40 AP-1 recognition element in yeast is mediated by a factor similar to AP-1 that is distinct from *GCN4*. Cell.

[36] Harvey I, Bossomaier T: Time out of joint: attractors in asynchronous random Boolean networks. In *Proceedings of the Fourth European Conference on Artificial Life*. Edited by Husbands P, Harvey I. Cambridge, MA; MIT Press; 1997: pp. 67–75.

[37] Hasan R, Leroy C, Isnard AD, Labarre J, Boy-Marcotte E, Toledano MB (2002). The control of the yeast H_2_O_2_ response by the Msn2/4 transcription factors. Mol Microbiol.

[38] Helikar T, Kochi N, Konvalina J, Rogers JA (2011). Boolean modeling of biochemical networks. The Open Bioinformatics Journal.

[39] Høj A, Jakobsen BK (1994). A short element required for turning off heat shock transcription factor: evidence that phosphorylation enhances deactivation. EMBO J.

[40] Holland SL, Reader T, Dyer PS, Avery SV (2014). Phenotypic heterogeneity is a selected trait in natural yeast populations subject to environmental stress. Environ Microbiol.

[41] Huang S, Eichler G, Bar-Yam Y, Ingber DE (2005). Cell fates as high-dimensional attractor states of a complex gene regulatory network. Phys Rev Lett.

[42] Iida H (1988). Multistress resistance of *Saccharomyces cerevisiae* is generated by insertion of retrotransposon Ty into the 5' coding region of the adenylate cyclase gene. Mol Cell Biol.

[43] Kataoka T, Broek D, Wigler M (1985). DNA sequence and characterization of the *S. cerevisiae* gene encoding adenylate cyclase. Cell.

[44] Kauffman S (1969). Metabolic stability and epigenesis in randomly constructed genetic nets. J Theoret Biol.

[45] Kim B-H, Schöffl F (2002). Interaction between *Arabidopsis* heat shock transcription factor 1 and 70 kDa heat shock proteins. J Experiment Bot.

[46] Kim J, Vandamme D, Kim J-R, Garcia Munoz A, Cho K-H (2014). Robustness and Evolvability of the Human Signaling Network. PLoS Comput Biol.

[47] Kirk N, Piper PW (1991). The determinants of heat-shock element-directed *lacZ* expression in *Saccharomyces cerevisiae*. Yeast.

[48] Klipp E, Liebermeister W, Wierling C, Kowald A, Lehrach H, Herwig R. Systems Biology: A Textbook. Wiley-VCH; 2009.

[49] Krems B, Charizanis C, Entian KD (1996). The response regulator-like protein Pos9/Skn7 of *Saccharomyces cerevisiae* is involved in oxidative stress resistance. Curr Genet.

[50] Lee P, Cho B-R, Joo H-S, Hahn J-S (2008). Yeast Yak1 kinase, a bridge between PKA and stress-responsive transcription factors, Hsf1 and Msn2/Msn4. Mol Microbiol.

[51] Lee P, Kim MS, Paik S-M, Choi S-H, Cho B-R, Hahn J-S (2013). Rim15-dependent activation of Hsf1 and Msn2/4 transcription factors by direct phosphorylation in *Saccharomyces cerevisiae*. FEBS Letters.

[52] Li F, Long T, Lu Y, Ouyang Q, Tang C (2004). The yeast cell-cycle network is robustly designed. Proc Natl Acad Sci USA.

[53] Lidstrom ME, Konopka MC (2010). The role of physiological heterogeneity in microbial population behavior. Nat Chem Biol.

[54] Longtine MS, Mckenzie A, Demarini DJ, Shah NG, Wach A, Brachat A (1998). Additional modules for versatile and economical PCR-based gene deletion and modification in *Saccharomyces cerevisiae*. Yeast.

[55] Ma P, Wera S, Van Dijck P, Thevelein JM (1999). The *PDE1*-encoded low-affinity phosphodiesterase in the yeast *Saccharomyces cerevisiae* has a specific function in controlling agonist-induced cAMP signaling. Mol Biol Cell.

[56] Marchler G, Schuller C, Adam G, Ruis H (1993). A *Saccharomyces cerevisiae* UAS element controlled by protein kinase A activates transcription in response to a variety of stress conditions. EMBO J.

[57] Milenkovic L, Scott MP. Not lost in space: trafficking in the Hedgehog signalling pathway. *Sci Signal* 2010, 3 p. pe14 doi:10.1126/scisignal.3117pe14. 10.1126/scisignal.3117pe14 20388915

[58] Mollapour M, Tsutsumi S, Donnelly AC, Beebe K, Tokita MJ, Lee MJ (2010). Swe1^Wee1^-dependent tyrosine phosphorylation of Hsp90 regulates distinct facets of chaperone function. Mol Cell.

[59] Nadeau K, Das A, Walsh CT (1993). Hsp90 chaperonins possess ATPase activity and bind heat shock transcription factors and peptidyl prolyl isomerases. J Biol Chem.

[60] Nieto-Sotelo J, Wiederrecht G, Okuda A, Parker CS (1990). The yeast heat shock transcription factor contains a transcriptional activation domain whose activity is repressed under nonshock conditions. Cell.

[61] Pan X, Heitman J (2002). Protein kinase A operates a molecular switch that governs yeast pseudohyphal differentiation. Mol Cell Biol.

[62] Park JI, Grant CM, Dawes IW (2005). The high-affinity cAMP phosphodiesterase of *Saccharomyces cerevisiae* is the major determinant of cAMP levels in stationary phase: involvement of different branches of the Ras-cyclic AMP pathway in stress responses. Biochem Biophys Res Commun.

[63] Pescini D, Cazzaniga P, Besozzi D, Mauri G, Amigoni L, Colombo S (2012). Simulations of the Ras/cAMP/PKA pathway in budding yeast highlights the establishment of stable oscillatory states. Biotecnol Adv.

[64] Pringle JR, Hartwell LH: The *Saccharomyces cerevisiae* cell cycle. In *The molecular biology of the yeast Saccharomyces*. Edited by Strathern J, Jones E, Broach J. Cold Spring Harbor, NY: Cold Spring Harbor Laboratory Press; 1982:97–142. vol. Life cycle and inheritance.

[65] Ptacek J, Devgan G, Michaud G, Zhu H, Zhu X, Fasolo J (2005). Global analysis of protein phosphorylation in yeast. Nature.

[66] Raitt DC, Johnson AL, Erkine AM, Makino K, Morgan B, Gross DS (2000). The Skn7 response regulator of *Saccharomyces cerevisiae* interacts with Hsf1 in vivo and is required for the induction of heat shock genes by oxidative stress. Mol Biol Cell.

[67] Reinders A, Burckert N, Boller T, Wiemken AT, De Virgilio C (1998). *Saccharomyces cerevisiae* cAMP-dependent protein kinase controls entry into stationary phase through the Rim15p protein kinase. Genes Dev.

[68] Robertson LS, Causton HC, Young RA, Fink GR (2000). The yeast A kinases differentially regulate iron uptake and respiratory function. Proc Natl Acad Sci USA.

[69] Robertson LS, Fink GR (1998). The three yeast A kinases have specific signaling functions in pseudohyphal growth. Proc Natl Acad Sci USA.

[70] Rose M, Botstein D (1983). Construction and use of gene fusions to *lacZ* (beta-galactosidase) that are expressed in yeast. Methods Enzymol.

[71] Saadatpour A, Albert R (2013). Boolean modeling of biological regulatory networks: A methodology tutorial. Methods.

[72] Sambrook J, Fritsch EF, Maniatis T. Molecular Cloning: a Laboratory Manual. Cold Spring Harbor, NY: Cold Spring Harbor Laboratory Press; 2nd edition; 1989.

[73] Shi Y, Mosser DD, Morimoto RI (1998). Molecular chaperones as HSF1-specific transcriptional repressors. Genes Dev.

[74] Sikorski RS, Hieter P (1989). A system of shuttle vectors and yeast host strains designed for efficient manipulation of DNA in *Saccharomyces cerevisiae*. Genetics.

[75] Smith A, Ward MP, Garrett S (1998). Yeast PKA represses Msn2p/Msn4p-dependent gene expression to regulate growth, stress response and glycogen accumulation. EMBO J.

[76] Sorger PK (1991). Heat shock factor and the heat shock response. Cell.

[77] Sorger PK, Pelham HR (1987). Purification and characterization of a heat-shock element binding protein from yeast. EMBO J.

[78] Stone DE, Craig EA (1990). Self regulation of 70-kilodalton heat shock proteins in *Saccharomyces cerevisiae*. Mol Cell Biol.

[79] Storici F, Lewis LK, Resnick MA (2001). *In vivo* site-directed mutagenesis using oligonucleotides. Nat Biotechnol.

[80] Szallasi Z, Stelling J, Periwal V. System Modeling in Cellular Biology: From Concepts to Nuts and Bolts. The MIT Press; 2006.

[81] Tamai KT, Liu X, Silar P, Sosinowski T, Thiele DJ (1994). Heat shock transcription factor activates yeast metallothionein gene expression in response to heat and glucose starvation via distinct signalling pathways. Mol Cell Biol.

[82] Tao W, Deschenes RJ, Fassler JS (1999). Intracellular glycerol levels modulate the activity of Sln1p, a *Saccharomyces cerevisiae* two-component regulator. J Biol Chem.

[83] Tatchell K (1986). *RAS* genes and growth control in *Saccharomyces cerevisiae*. J Bacteriol.

[84] Thevelein JM, De Winde JH (1999). Novel sensing mechanisms and targets for the cAMP-protein kinase A pathway in the yeast *Saccharomyces cerevisiae*. Mol Microbiol.

[85] Toda T, Cameron S, Sass P, Zoller M, Scott JD, McMullen B (1987). Cloning and characterization of *BCY1*, a locus encoding a regulatory subunit of the cyclic AMP-dependent protein kinase in *Saccharomyces cerevisiae*. Mol Cell Biol.

[86] Toda T, Cameron S, Sass P, Zoller M, Wigler M (1987). Three different genes in *S. cerevisiae* encode the catalytic subunits of the cAMP-dependent protein kinase. Cell.

[87] Toda T, Uno I, Ishikawa T, Powers S, Kataoka T, Broek D (1985). In yeast, RAS proteins are controlling elements of adenylate cyclase. Cell.

[88] Tudisca V, Recouvreux V, Moreno S, Boy-Marcotte E, Jacquet M, Portela P (2010). Differential localization to cytoplasm, nucleus or P-bodies of yeast PKA subunits under different growth conditions. Eur J Cell Biol.

[89] Vabulas RM, Raychaudhuri S, Hayer-Hartl M, Hartl FU (2010). Protein folding in the cytoplasm and the heat shock response. Cold Spring Harb Perspect Biol.

[90] Wang R-S, Saadatpour A, Albert R (2012). Boolean modeling in systems biology: an overview of methodology and applications. Phys Biol.

[91] Werner-Washburne M, Braun E, Johnston GC, Singer RA (1993). Stationary phase in the yeast *Saccharomyces cerevisiae*. Microbiol Rev.

[92] Werner-Washburne M, Stone DE, Craig EA (1987). Complex interactions among members of an essential subfamily of hsp70 genes in *Saccharomyces cerevisiae*. Mol Cell Biol.

[93] Williamson T, Schwartz JM, Kell DB, Stateva L (2009). Deterministic mathematical models of the cAMP pathway in *Saccharomyces cerevisiae*. BMC Systems Biology.

[94] Wu C (1995). Heat shock transcription factors: structure and regulation. Annu Rev Cell Dev Biol.

[95] Wu WS, Li WH (2008). Identifying gene regulatory modules of heat shock response in yeast. BMC Genomics.

[96] Zou J, Guo Y, Guettouche T, Smith DF, Voellmy R (1998). Repression of heat shock transcription factor HSF1 activation by HSP90 (HSP90 complex) that forms a stress-sensitive complex with HSF1. Cell.

