# Supplementary material for: Complex regulation of Hsf1-Skn7 activities by the catalytic subunits of PKA in Saccharomyces cerevisiae: experimental and computational evidences
Source: BMC Syst Biol. 2015 Jul 27;9:42. doi: 10.1186/s12918-015-0185-8 (PMC4515323; doi:10.1186/s12918-015-0185-8)
Supplement: Additional file 1: — Table S1. Growth and thermotolerance of WT yeast strains and mutants deleted in CDC25, RAS2 or BCY1. Table S2. Contribution of Hsf1 and Skn7 to the elevated thermotolerance and slow growth rate of the cdc25Δ mutant. Table S3. Growth and thermotolerance of WT or mutant yeast strains deleted in TPK1, TPK2 or TPK3. Table S4. Relative β-galactosidase activity values for all strains used in this work. Table S5. Strains used in this study. [file 12918_2015_185_MOESM1_ESM.pdf]

**Table S1.** Growth and thermotolerance of WT yeast strains and mutants deleted in *CDC25*, *RAS2* or *BCY1*.

Cultures were grown to mid-exponential phase at 25 °C in SD medium. Duplication time and basal thermotolerance were determined as described in Materials and Methods section. Mutant strains and their isogenic WT strain are shown in the same group. Values are the average and standard deviation of at least three independent experiments. Asterisks indicate a statistically significant difference between the indicated mutant and its isogenic WT strain according to one-way ANOVA followed by Bonferroni post-test: ***P* $< 0.01$; ****P* $< 0.001$.

| Strain (genotype) | Duplication time (h) | Basal thermotolerance (%) |
|---|---|---|
| W303-6B (WT) | 2.3 ± 0.1 | 0.02 ± 0.01 |
| SL5001 (*cdc25Δ*) | 5.1 ± 0.3*** | 70 ± 12*** |
| | | |
| W303-1a (WT) | 2.5 ± 0.2 | 0.02 ± 0.02 |
| Wras2Δ (*ras2Δ*) | 2.7 ± 0.2 | 2.4 ± 1.5 *** |
| CM0095 (*bcy1Δ*) | 2.1 ± 0.1 ** | 0.03 ± 0.02 |

**Table S2.** Contribution of Hsf1 and Skn7 to the elevated thermotolerance and slow growth rate of the *cdc25Δ* mutant.

Cultures were grown to mid-exponential phase at 25 °C in SD medium. Duplication time and basal thermotolerance were determined as described in Materials and Methods section. Strains are derived from W303. Values are the average and standard deviation of at least three independent experiments. On each column, values with different letters differ significantly ($P < 0.05$) according to one-way ANOVA followed by Bonferroni post-test.

| Strain (genotype) | Duplication time (h) | Basal thermotolerance (%) |
| --- | --- | --- |
| W303-6B (WT) | 2.3 ± 0.1 [d] | 0.02 ± 0.01 [c] |
| SL5001 (*cdc25Δ*) | 5.1 ± 0.3 [a] | 70 ± 12 [a] |
| LM020 (*hsf1-ΔCTA*) | 2.8 ± 0.03 [c] | 0.02 ± 0.01 [c] |
| SL6001 (*hsf1-ΔCTA cdc25Δ*) | 5.4 ± 0.1 [a] | 15 ± 11.3 [bc] |
| SE1000 (*skn7Δ*) | 2.3 ± 0.02 [d] | 0.02 ± 0.01 [c] |
| SL4001 (*skn7Δ cdc25Δ*) | 4.7 ± 0.07 [b] | 30.5 ± 4 [b] |

**Table S3.** Growth and thermotolerance of WT or mutant yeast strains deleted in *TPK1*, *TPK2* or *TPK3*.

Cultures were grown to mid-exponential phase at 25 °C in SD medium. Duplication time and basal thermotolerance were determined as described in Materials and Methods section. Strains are derived from W303. Values are the average and standard deviation of at least three independent experiments. Asterisks indicate a statistically significant difference between the indicated mutant and its isogenic WT strain according to one-way ANOVA followed by Bonferroni post-test: *$P < 0.05$; **$P < 0.01$; ***$P < 0.001$.

| Strain (genotype) | Duplication time (h) | Basal thermotolerance (%) | Induced thermotolerance (%) |
|---|---|---|---|
| W303-1a (WT) | 2.3 ± 0.2 | 0.02 ± 0.02 | 70 ± 9 |
| KG712 (*tpk1Δ*) | 4.2 ± 0.2*** | 0.22 ± 0.05 | 54 ± 19* |
| KG604 (*tpk2Δ*) | 2.3 ± 0.1 | 0.05 ± 0.03 | 41 ± 19** |
| KS580 (*tpk3Δ*) | 2.5 ± 0.2 | 0.05 ± 0.04 | 66 ± 16 |
| KS590 (*tpk2Δ tpk3Δ*) | 2.3 ± 0.1 | 0.02 ± 0.03 | 63 ± 9 |
| KS700 (*tpk1Δ tpk3Δ)* | 3.8 ± 0.3*** | 1.35 ± 0.52*** | 71 ± 12 |
| KG710 (*tpk1Δ tpk2Δ)* | 2.4 ± 0.2 | 0.08 ± 0.02 | 54 ± 14* |

**Table S4.** Relative β-galactosidase activity values for all strains used in this work. Temperature of the measurement is indicated by the number preceding the low hyphen (25_ is 25°C). Values are expressed as the quotient strain/WT at 25°C. In each case, WT strain refers to the corresponding isogenic WT strain for each mutant (shown in bold face). As can be seen, there is no significant difference if a different WT strain is used (values in parenthesis), indicating that mating type differences and tryptophan auxotrophy do not influence HSE-dependent gene expression.

| Strain | strain/W303-1a at 25°C | strain/W303-6B at 25°C |
|---|---|---|
| 25_*ssaΔ tpk2Δ* | **0.148218908** | (0.153621695) |
| 25_*tpk1Δ tpk2Δ* | **0.190072264** | (0.197000664) |
| 25_*tpk2Δ* | **0.216427741** | (0.224316836) |
| 25_*bcy1Δ* | **0.285724405** | (0.296139460) |
| 25_*tpk2Δ tpk3Δ* | **0.372915284** | (0.386508568) |
| 39_*ssa1Δ tpk2Δ* | **0.44303819** | (0.459187552) |
| 39_*tpk1Δ tpk2Δ* | **0.516215719** | (0.535032505) |
| 25_*skn7Δ* | (0.537727301) | **0.557328214** |
| 39_*tpk2Δ* | **0.579878082** | (0.60101545) |
| 25_*ssa2Δ tpk2Δ* | **0.618805163** | (0.641361477) |
| 39_*bcy1Δ* | **0.653442030** | (0.677260907) |
| 25_*tpk1Δ tpk3Δ* | **0.691029832** | (0.716218836) |
| 25_*tpk1Δ* | **0.704670726** | (0.730356961) |
| 39_*tpk2Δ tpk3Δ* | **0.717740151** | (0.743902784) |
| 39_*ssa2Δ tpk2Δ* | **0.79872881** | (0.827843593) |

| | | |
|---|---|---|
| 25_*skn7Δ cdc25Δ* | (0.841549497) | **0.872225154** |
| 25_*tpk2Δ* | **0.866466575** | (0.898050495) |
| 25_*hsf1-ΔCTA* | (0.92167627) | **0.95527266** |
| 25_W303-6B | (0.964830575) | **1** |
| 25_W3031-a | **1** | (1.0364514) |
| 25_*tpk3Δ* | **1.08863227** | (1.12831444) |
| 25_*ssa1Δ ssa2Δ tpk2Δ* | **1.381222022** | (1.431569498) |
| 39_*tpk1Δ* | **1.447581865** | (1.500348251) |
| 25_*ssa1Δ* | **1.464506249** | (1.517889552) |
| 39_*ssa1Δ ssa2Δ tpk2Δ* | **1.674806117** | (1.735855145) |
| 39_*skn7Δ cdc25Δ* | (1.709385544) | **1.771695041** |
| 39_*tpk2Δ* | **1.830157041** | (1.896868828) |
| 25_*ras2Δ* | **1.889514230** | (1.958389669) |
| 39_*hsf1-ΔCTA* | (1.149084106) | **1.19096983** |
| 39_*skn7Δ* | (2.143106871) | **2.221226116** |
| 39_*tpk1Δ tpk3Δ* | **2.152018292** | (2.230462371) |
| 39_W303-6B | (2.262514898) | **2.344986733** |
| 39_*tpk3Δ* | **2.427752985** | (2.516247979) |
| 39_*ras2Δ* | **2.518856004** | (2.610671832) |
| 39_W303-1a | **2.595151109** | (2.68974800) |
| 25_*hsf1-ΔCTA cdc25Δ* | (1.657430875) | **1.71784665** |
| 39_*ssa1Δ* | **3.175697746** | (3.291456374) |

| | | |
|---|---|---|
| 25_*cdc25Δ* | (3.376074632) | **3.2573400028** |
| 25_*ssa2Δ* | **3.802821451** | (3.941439616) |
| 39_*cdc25Δ* | (3.494700391) | **3.371793787** |
| 39_*hsf1-ΔCTA cdc25Δ* | (1.947576697) | **2.018568594** |
| 39_*ssa2Δ* | **7.563223772** | (7.838913867) |
| 25_*ssa1Δ ssa2Δ* | **9.824683073** | (10.18280652) |
| 39_*ssa1Δ ssa2Δ* | **12.01638101** | (12.45439492) |

**Table S5.** Strains used in this study

| Strain | Description | Parent strain | Source |
|---|---|---|---|
| CM0095 | *MAT***a** *can1-100 ade2-1 his3-11,15 leu2-3,112 trp1-1 ura3-1 bcy1Δ::kanMX6* | W303 | 1 |
| JF3000 | *MAT***a**/*MATα can1-100/can1-100 ade2-1/ade2-1 his3-11,15/his3-11,15 leu2-3,112/LEU2 trp1-1/TRP1 ura3-1/ura3-1 CD25/cdc25Δ::loxP-kanMX4-loxP* | W303 | 1 |
| JF3100 | *MAT***a**/*MATα can1-100/can1-100 ade2-1/ade2-1 his3-11,15/his3-11,15 leu2-3,112/LEU2 trp1-1/TRP1 ura3-1/ura3-1 CD25/cdc25Δ::loxP* | W303 | 1 |
| KG604 | *MAT***a** *leu2-3,112 trp1-1 can1-100 ura3-1 ade2-1 his3-11,15 tpk2Δ::loxP* | W303 | 1 |
| KG712 | *MAT***a** *leu2-3,112 trp1-1 can1-100 ura3-1 ade2-1 his3-11,15 tpk1Δ::kanMX6* | W303 | 1 |
| KS580 | *MAT***a** *leu2-3,112 trp1-1 can1-100 ura3-1 ade2-1 his3-11,15 tpk3Δ::loxP* | W303 | 1 |
| KS590 | *MAT***a** *leu2-3,112 trp1-1 can1-100 ura3-1 ade2-1 his3-11,15 tpk2Δ::loxP tpk3Δ::loxP* | W303 | 1 |
| KS590-URA3 | *MAT***a** *leu2-3,112 trp1-1 can1-100 ura3-1 ade2-1 his3-11,15 tpk2Δ::URA3 tpk3Δ::loxP* | W303 | 1 |

| | | | |
|---|---|---|---|
| KS590-URA3-TPK2 | *MAT***a** *leu2-3,112 trp1-1 can1-100 ura3-1 ade2-1 his3-11,15 tpk2Δ::TPK2 tpk3Δ::loxP* | W303 | 1 |
| KS700 | *MAT***a** *leu2-3,112 trp1-1 can1-100 ura3-1 ade2-1 his3-11,15 tpk1Δ::kanmx6::NAT3 tpk3Δ::loxP* | W303 | 1 |
| KS710 | *MAT***a** *leu2-3,112 trp1-1 can1-100 ura3-1 ade2-1 his3-11,15 Δtpk1::kanMX6 Δtpk2::loxP* | W303 | 1 |
| LM020 | *MATα can1-100 ade2-1 his3-11,15 leu2-3,112 ura3-1 hsf1-ΔCTA::kanMX6* | W303 | 1 |
| S001 | *MAT***a** *leu2-3,112 trp1-1 can1-100 ura3-1 ade2-1 his3-11,15 Δssa1::kanMX6* | W303 | 1 |
| S002 | *MAT***a** *leu2-3,112 trp1-1 can1-100 ura3-1 ade2-1 his3-11,15 tpk2Δ::loxP Δssa1::kanMX6* | W303 | 1 |
| SE1000 | *MATα can1-100 ade2-1 his3-11,15 leu2-3,112 ura3-1 skn7Δ::kanMX6* | W303 | 1 |
| SL622 | *MAT***a** *leu2-3,112 trp1-1 can1-100 ura3-1 ade2-1 his3-11,15 Δssa2::kanMX6* | W303 | 1 |
| SL622-N | *MAT***a** *leu2-3,112 trp1-1 can1-100 ura3-1 ade2-1 his3-11,15 Δssa2:: kanmx6::NAT3* | W303 | 1 |
| SL623 | *MAT***a** *leu2-3,112 trp1-1 can1-100 ura3-1 ade2-1 his3-11,15 tpk2Δ::loxP Δssa2::kanMX6* | W303 | 1 |
| SL623-N | *MAT***a** *leu2-3,112 trp1-1 can1-100 ura3-1 ade2-1* | W303 | 1 |

| | | | |
|---|---|---|---|
| | *his3-11,15 tpk2Δ::loxP Δssa2:: kanmx6::NAT3* | | |
| SL625 | *MAT***a** *leu2-3,112 trp1-1 can1-100 ura3-1 ade2-1 his3-11,15 Δssa1::HIS3 Δssa2::kanMX6* | W303 | 1 |
| SL708 | *MAT***a** *leu2-3,112 trp1-1 can1-100 ura3-1 ade2-1 his3-11,15 tpk2Δ::loxP Δssa1::kanMX6 Δssa2::kanmx6::NAT3* | W303 | 1 |
| SL4001 | *MATα can1-100 ade2-1 his3-11,15 leu2-3,112 ura3-1 skn7Δ::kanMX6 cdc25Δ::loxP-kanMX4-loxP* | W303 | 1 |
| SL5001 | *MATα can1-100 ade2-1 his3-11,15 leu2-3,112 ura3-1 cdc25Δ::loxP-kanMX4-loxP* | W303 | 1 |
| SL6001 | *MATα can1-100 ade2-1 his3-11,15 leu2-3,112 ura3-1 cdc25Δ::loxP hsf1-ΔCTA::kanMX6* | W303 | 1 |
| W303-1a | *MAT***a** *can1-100 ade2-1 his3-11,15 leu2-3,112 trp1-1 ura3-1* | W303 | 2 |
| W303-6B | *MATα can1-100 ade2-1 his3-11,15 leu2-3,112 ura3-1* | W303 | 3 |
| Wras2Δ | *MAT***a** *can1-100 ade2-1 his3-11,15 leu2-3,112 trp1-1 ura3-1 ras2Δ::kanMX4* | W303 | 1 |

[1] This study.

[2] Amoros & Estruch [20].

[3] R. Gaxiola.

**Table S6.** Sequences of oligonucleotides used in this work.

| Oligonucleotide | Sequence (5' → 3') | Target |
|---|---|---|
| ACT1-1 | CACTCTCCCATAACCTCCTA | *ACT1* |
| ACT1-2 | ATGGAAACGTAGAAGGCTGG | *ACT1* |
| fc-ssa3 | ATGACGAGGATGATGAGCAC | *SSA3* |
| FSHSP82 | GGTAGATTAGGTAGAACATC | *HSP82* |
| HSEA | AGAACTTTCCAGAACTTTCC | HSE |
| HSEB | GTTCTGGAAAGTTCTGGAAA | HSE |
| HSP12-F | ATGTCTGACGCAGGTAGAAA | *HSP12* |
| HSP12-R | TTACTTCTTGGTTGGGTCTT | *HSP12* |
| HSP26-F | ATGTCATTTAACAGTCCATT | *HSP26* |
| HSP26-R | TTAGTTACCCCACGATT | *HSP26* |
| rc-ssa3 | ACGTCAAAAGTACCACCACC | *SSA3* |
| RSHSP82 | CGCGTCGGAATAGTGAAAAC | *HSP82* |
| FTPK2-URA3 | TTAGGAAACAATCACGAGACGATAACGAC GGAATACCAAGTAACTATGCGGCATCAGAGC | *TPK2* |
| RTPK2URA3 | TTCAGCATATGGATCATCGCCTTGAATACC ATAATCTAGCCCTGATGCGGTATTTTCTCC | *TPK2* |
| TPK2-Lcl | CGAAGCGTTGCTTCATTTCAAC | *TPK2* |
| TPK2-Ucl | CAGCCGCCTCAAGATAAACCAG | *TPK2* |
