# Supplementary material for: Complex regulation of Hsf1-Skn7 activities by the catalytic subunits of PKA in Saccharomyces cerevisiae: experimental and computational evidences
Source: BMC Syst Biol. 2015 Jul 27;9:42. doi: 10.1186/s12918-015-0185-8 (PMC4515323; doi:10.1186/s12918-015-0185-8)
Supplement: Additional file 2: — Figure S1. Correlation analysis between pRY016 plasmid copy number and HSE-dependent gene expression. Figure S2. Levels of HSP transcripts in WT and tpk2Δ tpk3Δ mutant. Figure S3. Comparison of different updating schemes. Figure S4. cAMP-dependent PKA activity in extracts of WT and TPK deletion mutants. Figure S5. Reduced network used to compute the structure of the attractor landscape. Figure S6. Structure of the attractor landscape displayed by the WT reduced network shown in Figure S5C. [file 12918_2015_185_MOESM2_ESM.pdf]

Figure S1. Correlation analysis between pRY016 plasmid copy number and HSE-dependent gene expression. Twenty six pairs of data of plasmid copy number vs β-galactosidase activity from deletion mutants used in this work were analysed using Pearson's correlation coefficient. Measurements were obtained from exponential cell growth at 25°C or after cultures received a single heat shock at 39°C for 1 hour. The correlation was not statistically significant ($P = 0.0765$).

Figure S2. Levels of *HSP* transcripts in WT and *tpk2Δ tpk3Δ* mutant. Relative amounts of *HSP104*, *HSP82*, *SSA3*, *HSP26*, *HSP12*, and *ACT1* transcripts were estimated by RNA blot hybridization during optimal temperature growth (25 °C) and after heat shock (39 °C). (A) autoradiograms, (B) transcript levels relative to the WT cells. Strains assayed were: WT (W303-1a) and *tpk1Δ tpk2Δ* (KG710).

Figure S3. Comparison of different updating schemes. The *HSE-lacZ* expression rations (given strain/WT strain 25°C) for the experimental data and for different synchronous and asynchronous updating schemes were calculated. Graph displays the experimental observations (black columns) or the numerical results using the synchronous WDM (red columns), Deterministic Asynchronous updating (blue and purple columns, respectively), and Random Asynchronous updating (green columns). The WDM model was used for the Deterministic Asynchronous update because with this procedure it is possible to obtain the basins of attraction. ICA stands for Initial Condition Averaging. The ICA method averages the final state of the network after $T = 1000$ time steps, over many initial conditions. This method was used to obtain a single value from both asynchronous updating schemes. It can

be observed that essentially the same results were obtained regardless of the updating and averaging schemes.

Figure S4. cAMP-dependent PKA activity in extracts of WT and *TPK* deletion mutants. Equal amounts of protein from total extracts obtained from WT and *TPK* deletion mutants were assayed with the Pep Tag® protocol as described in Methods. Activity was assayed in the absence or presence of cAMP. No activity was detected in the absence of cAMP. Data shown corresponds to assays where cAMP was added. Strains assayed were: WT (W303-1a), *tpk1Δ* (KG712), *tpk2Δ* (KG604), *tpk3Δ* (KS580), *tpk2Δ tpk3Δ* (KS590), *tpk1Δ tpk3Δ* (KS700), *tpk1Δ tpk2Δ* (KG710).

Figure S5. Reduced Network used to compute the structure of the attractor landscape. (A) Network showing all elements used in the Results section of the main manuscript. (B) Removal of mimicking/intermediary elements. Ras2 and cAMP are regulated positively in a cascade mode by Cdc25 and their regulation is linear, similar to Hsf1 with respect to *HSE-lacZ*. Therefore, when these elements were removed from the network, a very similar dynamical behavior was observed. This reduction facilitated the computing of the whole set of possible initial conditions for the system and the visualization of their corresponding basins of attraction. (C) Final form of the reduced network.

Figure S6. Structure of the attractor landscape displayed by the WT reduced network shown in Figure S5C. The reduced network has approximately 10^7states (the full network has more than 10^9states, which are computationally intractable). These states organize into

attraction basins corresponding to different attractors. Each network state is represented by a point. Two points are interconnected whenever one point is the precursor of the second point under the dynamics. The fan-like structures represent all the precursors of one state. The arrows indicate the flow of the dynamics, which eventually converge to the attractors. In this case we found six attractors, each one with its corresponding basin of attraction. Three attractors consisted of four states, one attractor of two states and two attractors of only one state. The heterogeneity of the attractors and their basins of attraction illustrate the necessity to perform a weighted average when computing expression levels used to compare with measurements performed in batch-cultures.

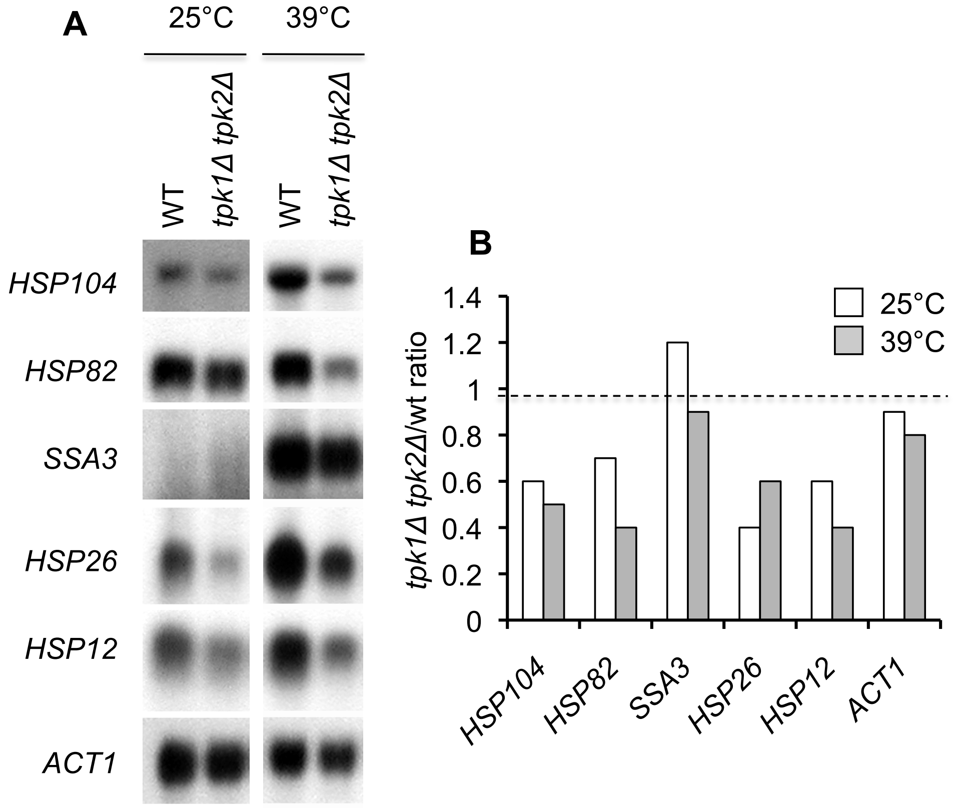

A
25°C
39°C
WT
tpk1Δ tpk2Δ
WT
tpk1Δ tpk2Δ
HSP104
HSP82
SSA3
HSP26
HSP12
ACT1
B
25°C
39°C
tpk1Δ tpk2Δ/wt ratio
1.4
1.2
1
0.8
0.6
0.4
0.2
0
HSP104
HSP82
SSA3
HSP26
HSP12
ACT1

Fig. S1

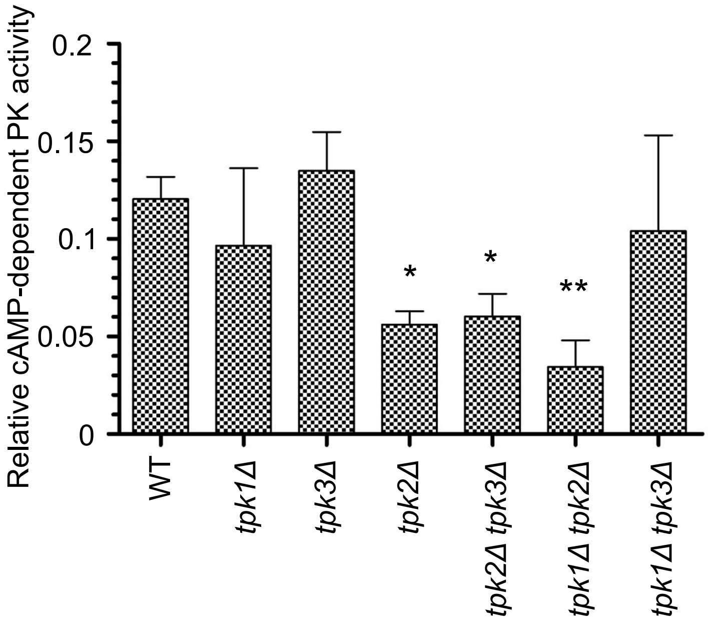
Relative cAMP-dependent PK activity
0.2
0.15
0.1
0.05
0
*
*
**
WT
tpk1Δ
tpk3Δ
tpk2Δ
tpk2Δ tpk3Δ
tpk1Δ tpk2Δ
tpk1Δ tpk3Δ

Fig. S2

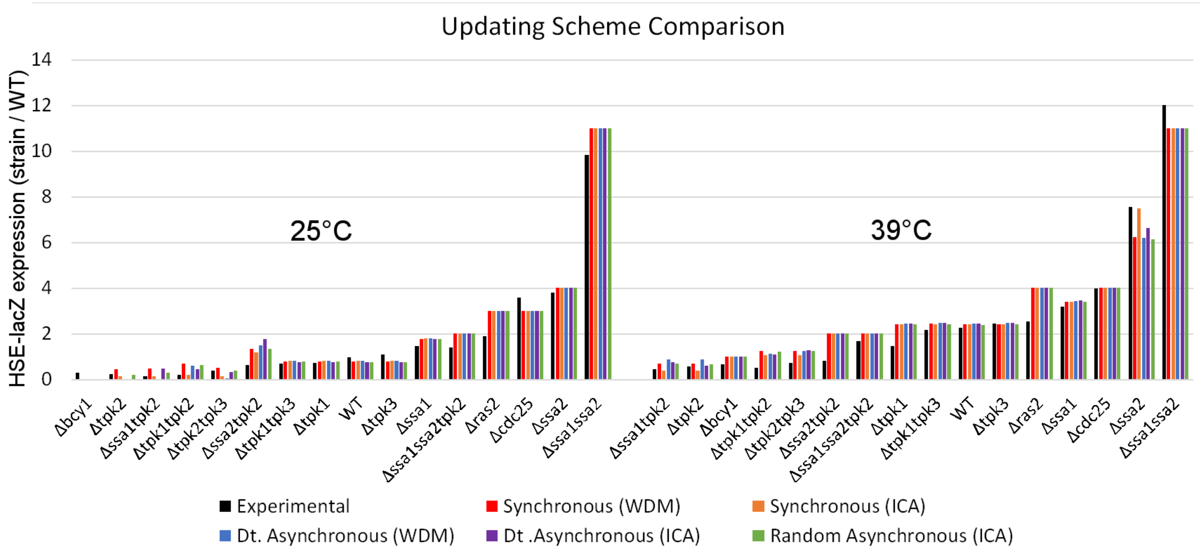

Updating Scheme Comparison
HSE-lacZ expression (strain / WT)
0
2
4
6
8
10
12
14
25°C
39°C
Δbcy1
Δtpk2
Δssa1tpk2
Δtpk1tpk2
Δtpk2tpk3
Δssa2tpk2
Δtpk1tpk3
Δtpk1
WT
Δtpk3
Δssa1
Δssa1ssa2tpk2
Δras2
Δcdc25
Δssa2
Δssa1ssa2
Δssa1tpk2
Δtpk2
Δbcy1
Δtpk1tpk2
Δtpk2tpk3
Δssa2tpk2
Δssa1ssa2tpk2
Δtpk1
Δtpk1tpk3
WT
Δtpk3
Δras2
Δssa1
Δcdc25
Δssa2
Δssa1ssa2
Experimental
Synchronous (WDM)
Synchronous (ICA)
Dt. Asynchronous (WDM)
Dt .Asynchronous (ICA)
Random Asynchronous (ICA)

Fig. S3

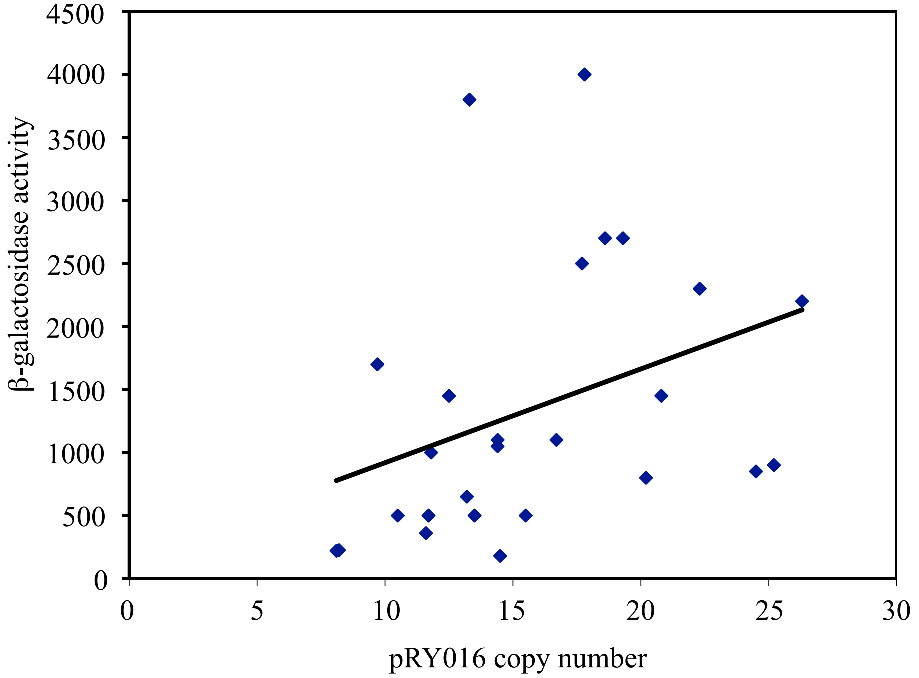

Pearson´s correlation coefficient = 0.35341449

Fig. S4

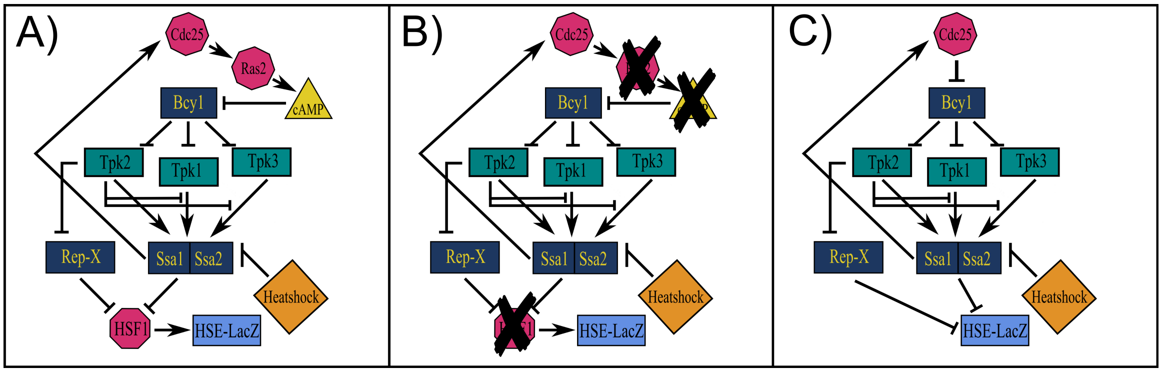
A)
Cdc25
Ras2
cAMP
Bcy1
Tpk2
Tpk1
Tpk3
Rep-X
Ssa1
Ssa2
Heatshock
HSF1
HSE-LacZ
B)
Cdc25
Bcy1
Tpk2
Tpk1
Tpk3
Rep-X
Ssa1
Ssa2
Heatshock
HSE-LacZ
C)
Cdc25
Bcy1
Tpk2
Tpk1
Tpk3
Rep-X
Ssa1
Ssa2
Heatshock
HSE-LacZ

Fig. S5

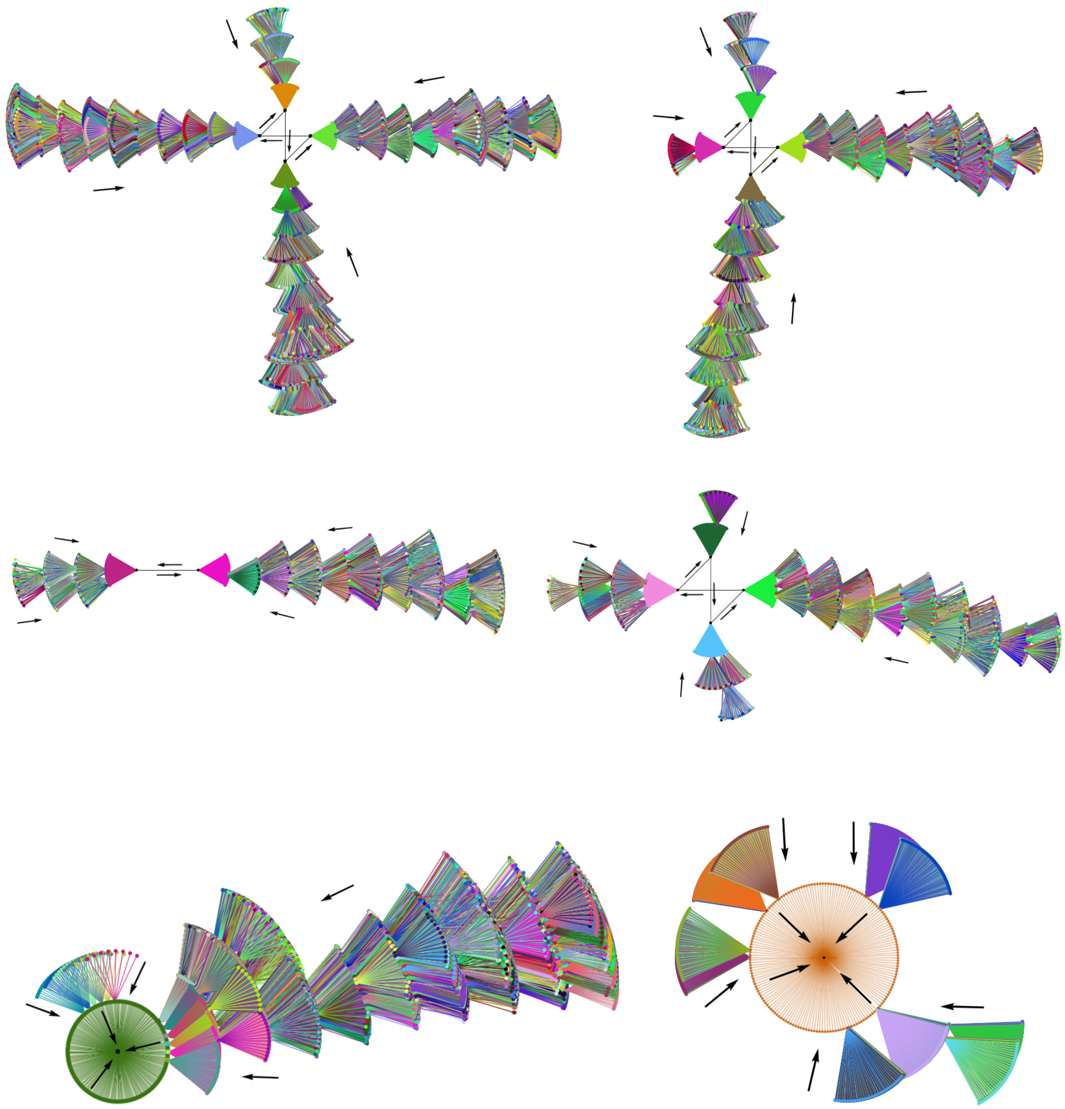

Fig. S6
