# Supplementary material for: Complex regulation of Hsf1-Skn7 activities by the catalytic subunits of PKA in Saccharomyces cerevisiae: experimental and computational evidences
Source: BMC Syst Biol. 2015 Jul 27;9:42. doi: 10.1186/s12918-015-0185-8 (PMC4515323; doi:10.1186/s12918-015-0185-8)
Supplement: Additional file 3: — Section 1. Synchronous and Asynchronous Updating Schemes. Section 2. Basins of attraction. Section 3. Assumptions and logical functions for the Windowed Discrete Model. Section 4. Logic Tables and discrete regulatory functions in the WT at 25 °C. [file 12918_2015_185_MOESM3_ESM.pdf]

Supplementary Information:

Section 1. Synchronous and Asynchronous Updating Schemes.

Apart from the synchronous updating scheme, we have implemented two other updating approaches: 1) A deterministic asynchronous (DA) update and 2) a random asynchronous (RA) update.

In the DA scheme, we randomly choose one permutation $\{l_1, l_2, l_3, \ldots, l_N\}$ of the numbers $\{1,2,3,\ldots,N\}$ (where N is the number of nodes). Then we update one node at each time step in the order given by that permutation. For instance, if the permutation is {4,2,3,1} for a network with 4 nodes, then we update the network elements in the order σ_4, σ_2, σ_3, σ_1 (one at a time). We continue repeating the updating of the network elements using always the same permutation. This is why in this case the updating scheme, although asynchronous, is deterministic (we always know which node is going to be updated next).

For the RA updating, at each time step we randomly choose one network element and update its state.

In both schemes, we take the time step Δt as $\Delta t = 1/N$, so that in one unit of time N nodes are updated. To obtain a single value from the simulation on both updating schemes, we implemented two different approaches. For the DA we use the WDM formalism and for both the DA and the RA we apply an initial condition averaging (ICA). This means that we run the simulation during a time T that is long enough for the system to reach the attractor at the synchronous updating scheme. We then average over many initial conditions the state reached by the system at that time T. We also implemented the ICA approach for the synchronous model.

Figure S3 in the Supplementary Information shows that essentially the same results are obtained with all the updating and averaging schemes: Synchronous WDM, Synchronous ICA, DA with WDM, DA with ICA, and random asynchronous with ICA.

The fact the these updating schemes produce essentially the same results is very interesting, since it indicates that the WDM really captures the population average in batch cultures regardless of the specific updating scheme. To our knowledge, this is the first model with this property.

Section 2. Basins of attraction.

Here, we present a visual representation of the basins of attraction for the WT network. To calculate these basins, one has to follow the trajectory of all the possible initial conditions until an attractor is reached. Due to the huge number of possible initial conditions for our multiple state network (around $4x10^9$ conditions), we reduced the size of our network by eliminating the linear regulations of Cdc25 -> Ras2, Ras2 -> cAMP, Hsf1 -> *HSE-lacZ* as illustrated in Figure S5. The elimination of these intermediate regulations, which only introduce temporal delays in the response of the target nodes, produced a much smaller set of possible states (1.9 x 10^7 conditions), which made a full exploration of the state space tractable. The attractors and their corresponding basins of attraction for this reduced network are presented in Figure S6. Note that the reduced networks were used only to generate the visual display presented in Fig. S6. However, we include a text file (Text S1) containing the complete set of attractors for the full WT network and the mutants *bcyΔ*, *ssa1Δ ssa2Δ*, and *tpk1Δ tpk3Δ*. We chose these mutants because they exhibited attractors with high and low *HSE-lacZ* expression.

Section 3. *Assumptions and logical functions for the Windowed Discrete Model.*

Because the exact strengths of the interactions occurring within the PKA-RN are not completely known, we made the following assumptions to construct the Logic Tables presented in Section 4.

Node	Assumption
**Cdc25**	Cdc25 is regulated positively by Ssa1 and Ssa2 chaperones (Hsp70), which stabilize the Cdc25 protein [1]. In response to a heat shock, the Cdc25-Hsp70 complex dissociates causing a strong reduction in both Cdc25 levels and PKA pathway activity [1]. In our model [2, 3, 4], we considered Ssa2 as the main regulator of Cdc25, whereas Ssa1 only contributed mildly to its activation, as this fitted better our experimental data [5].
**Ras2**	Ras2 is regulated by Cdc25 [6, 7]. In turn, Ras2 positively regulates adenylate cyclase [8].
**Cyr1**	Adenylate cyclase is positively and linearly regulated by Ras1 and Ras2 [8, 9]. In our model, its role can be substituted by a direct regulation from Ras1 and Ras2 to cAMP [2, 3, 4].
**cAMP**	cAMP is the product of adenylate cyclase activity [10, 11]. In our model cAMP is positively, linearly, and directly regulated by Ras1 and Ras2 [2, 3, 4].
**Bcy1**	Bcy1 is the negative regulatory subunit of the CS (Tpk1, Tpk2, and Tk3) [10, 12, 13]. In our model, Bcy1 is negatively and linearly regulated by cAMP [2, 3, 4].
**Tpk1, Tpk3**	Tpk1 and Tpk2 are two out of three CS of PKA [13]. Tpk1 and Tpk2 are inhibited by Bcy1 [10, 12, 13]. In our model, Tpk1 and Tpk3 have four functional states and become totally inhibited at high levels of Bcy1 [3, 4].
**Tpk1*, Tpk3***	Active states of Tpk1 and Tpk3 that, according to our experimental data, come up in the absence of Tpk2 [5, 14, 15]. According to our model [3, 4], levels of Tpk2 ≥2 (it has 6 possible states) suffice to suppress their functionality. The active states of Tpk1 and Tpk3 (Tpk1* and Tpk3*) are included to emphasize that Tpk2 is able to inhibit their activity over Ssa1 and Ssa2, but not necessarily over other possible substrates [3, 4].
**Tpk2**	Tpk2 is one out of three CS of PKA [13]. Tpk2 is also inhibited by Bcy1 [10, 12, 13]. In our model [3, 4], Tpk2 has 6 functional states (levels 0-5), as it is not completely repressed by Bcy1 as Tpk1/Tpk3. (Activity: 0 = no activity, 1 = minimum, 2 = low, 3 = medium, 4 = high, 5 = maximum activity). At high levels of Bcy1 (≥ 4) Tpk2 still has some activity (level = 1-2). The maximum level of activation of Tpk2 (level 5) corresponds to the maximum level of activation of Tpk1/Tpk3 (level 3) [3, 4].
**RepX**	RepX is the putative repressor of Hsf1 that, according to our data [15], becomes active when Tpk2 is absent or at minimum levels. RepX has 3 functional states (0-2) and levels of Tpk2 ≥1 are enough to suppress its activity completely [3, 4]. RepX maintains Hsf1/Skn7 activity close to that of a WT cell when acting as the sole repressor of Hsf1/Skn7 in the absence of Tpk2 (i.e., in cells *tpk2Δ ssa1Δ ssa2Δ*). In the absence of Tpk2 (i.e., in *tpk2Δ* cells) acts synergistically with Ssa1 and Ssa2 to fully repress Hsf1/Skn7 [15].

**Ssa1**	Ssa1 is a chaperone of the Hsp70 family that favors the stability of Cdc25 [1] and, according to our data [15], becomes activated by the CS of PKA. Ssa1 must inhibit Hsf1 activity, as it is known that: a) Hsp70 chaperones repress Hsf1 function [16, 17] and b) Ssa1 and Ssa2 are the most abundant Hsp70s during normal growth [1]. In our model, Ssa1 has only two functional states (0,1). Levels ≥1 of any of the Tpks are enough to activate it. We estimate that, in a WT cell, Tpk2 is the major activator of Ssa1. In the absence of Tpk2, or at very low levels of Tpk2, Tpk1 and Tpk3 also activate Ssa1 [3, 4].
**Ssa2**	Similar to Ssa1, Ssa2 favors the stability of Cdc25 [1] and, according to our data [15], becomes activated by the CS of PKA. Ssa2 must inhibit Hsf1 activity, as it is known that: a) Hsp70 chaperones repress Hsf1 function [16, 17] and b) Ssa2 and Ssa1 are the most abundant Hsp70s during normal growth [1]. We estimate that, in a WT cell, Tpk2 is the major activator of Ssa2. In the absence of Tpk2, or at very low levels of Tpk2, Tpk1 and Tpk3 also activate Ssa2 [3, 4].
**Hsf1, Skn7**	Hsf1 and Skn7 are transcription factors that recognize the HSE element in the promoters of a large number of stress genes [18, 19]. According to our data [15], and to information in the literature [16, 17], Hsf1/Skn7 are repressed by Ssa1 and Ssa2. Our modeling of the PKA-RN indicates that Hsf1/Skn7 are also inhibited by a third repressor designated RepX. In our model, Ssa1 only represses Hsf1/Skn7 mildly, while a maximum expression of Ssa2 reduces almost all of its activity [2, 3, 4].
HSE-lacZ	*HSE-lacZ* is our reporter gene and its expression is dependent on Hsf1/Skn7 transcription factors that promote the expression of stress and housekeeping genes [19, 20, 21, 22]. According to our data, the activity of the *HSE-lacZ* reporter is influenced by the PKA-RN and by temperature [5, 14, 15, 23, 24]. In our model, the *HSE-lacZ* reporter is positively and linearly activated by Hsf1/Skn7 [2, 3, 4].
**Heat Shock**	Heat shock causes the accumulation of denatured proteins. As a result, Hsp70 chaperones (i.e., Ssa1, Ssa2) reduce their binding to Hsf1, the transcriptional activity of Hsf1 is de-repressed [16, 17, 20], and the stability of Cdc25 decays [1]. In consequence, the activity of the CS of PKA and the repression of Hsf1 diminishes, whereas the expression of *HSE-lacZ* increases. In our model, the activity of Ssa2 is reduced whenever the Heat Shock node is turned on [2, 3, 4]. In contrast, Ssa1 activity is not influenced after turning on the Heat Shock node because, compared to Ssa2, its contribution to Hsf1 repression is negligible, as our experimental data shows [15].

Section 4. Logic Tables and discrete regulatory functions in the WT at 25°C.

A total of 15 Tables are presented (Table SI1 to SI15). The name of the regulated element (node) appears between << and >> symbols. Indicated in parenthesis is the presence (true) or absence (false) of the node. In WT cells at 25°C all the nodes are present (true) with the exception of the Heat Shock node (false). The regulators of each node are located on the columns located to the left of the node. In the heading of each Table are indicated the number of entries and the sources in the literature, or in our own data, that support the logic functions.

Table SI1. Cdc25 node.

Table entries: 36; references: 1, 5.

Ssa1	Ssa2	<<Cdc25>> (true)
0	0	1
0	1	2
0	2	3
0	3	4
0	4	5
0	5	x
1	0	2
1	1	2
1	2	3
1	3	4
1	4	5
1	5	x
2	0	x
2	1	x
2	2	x
2	3	x
2	4	x
2	5	x
3	0	x
3	1	x
3	2	x
3	3	x
3	4	x
3	5	x
4	0	x
4	1	x
4	2	x
4	3	x
4	4	x
4	5	x
5	0	x
5	1	x
5	2	x
5	3	x

5	4	x
5	5	x

**Table SI2.** Ras2 node.	
Table entries: 6; references: 6, 7.	
**Cdc25**	**<<Ras2>> (true)**
0	0
1	1
2	2
3	3
4	4
5	5

**Table SI3.** cAMP node.	
Table entries: 6; references: 8, 9, 10.	
**Ras2**	**<<cAMP>> (true)**
0	0
1	1
2	2
3	3
4	4
5	5

**Table SI4.** Bcy1 node.	
Table entries: 6; references: 10, 11, 12.	
**cAMP**	**<<Bcy1>> (true)**
0	5
1	4
2	3
3	2
4	1
5	0

**Table SI5.** Tpk1 node.	
Table entries: 6; references: 10, 12, 13.	
**Bcy1**	**<<Tpk1>> (true)**
0	3
1	2
2	2
3	1
4	0
5	0

**Table SI6.** Tpk2 node.	
Table entries: 6; references: 10, 12, 13.	
**Bcy1**	**<<Tpk2>> (true)**
0	5

1	4
2	4
3	3
4	2
5	1

Table SI7. Tpk3 node.

Table entries: 6; references: 10, 12, 13.

Bcy1	<<Tpk3>> (true)
0	3
1	2
2	2
3	1
4	0
5	0

Table SI8. Tpk1* node.

Table entries: 36; references: 5, 14, 15.

Tpk1	Tpk2	<<Tpk1*>> (true)
0	0	0
0	1	0
0	2	0
0	3	0
0	4	0
0	5	0
1	0	1
1	1	0
1	2	0
1	3	0
1	4	0
1	5	0
2	0	2
2	1	1
2	2	0
2	3	0
2	4	0
2	5	0
3	0	3
3	1	2
3	2	1
3	3	0
3	4	0
3	5	0
4	0	x
4	1	x
4	2	x
4	3	x
4	4	x

4	5	x
5	0	x
5	1	x
5	2	x
5	3	x
5	4	x
5	5	x

**Table SI9.** Tpk3* node.		
Table entries: 36; references: 5, 14, 15.		
**Tpk3**	**Tpk2**	**<<Tpk3*>> (true)**
0	0	0
0	1	0
0	2	0
0	3	0
0	4	0
0	5	0
1	0	1
1	1	0
1	2	0
1	3	0
1	4	0
1	5	0
2	0	2
2	1	1
2	2	0
2	3	0
2	4	0
2	5	0
3	0	3
3	1	2
3	2	1
3	3	0
3	4	0
3	5	0
4	0	x
4	1	x
4	2	x
4	3	x
4	4	x
4	5	x
5	0	x
5	1	x
5	2	x
5	3	x
5	4	x
5	5	x

Table SI10. RepX node.

Table entries: 6; references: 15.

Tpk2	<<RepX>> (true)
0	2
1	1
2	0
3	0
4	0
5	0

Table SI11. Ssa1 node.

Table entries: 1296; references: 15, 16, 17.

Heat	Tpk1*	Tpk2	Tpk3*	<<Ssa1>> (true)
0	0	0	0	0
0	0	0	1	0
0	0	0	2	1
0	0	0	3	1
0	0	0	4	x
0	0	0	5	x
0	0	1	0	0
0	0	1	1	1
0	0	1	2	1
0	0	1	3	1
0	0	1	4	x
0	0	1	5	x
0	0	2	0	1
0	0	2	1	1
0	0	2	2	1
0	0	2	3	1
0	0	2	4	x
0	0	2	5	x
0	0	3	0	1
0	0	3	1	1
0	0	3	2	1
0	0	3	3	1
0	0	3	4	x
0	0	3	5	x
0	0	4	0	1
0	0	4	1	1
0	0	4	2	1
0	0	4	3	1
0	0	4	4	x
0	0	4	5	x
0	0	5	0	1
0	0	5	1	1
0	0	5	2	1
0	0	5	3	1

0	0	5	4	x
0	0	5	5	x
0	1	0	0	0
0	1	0	1	1
0	1	0	2	1
0	1	0	3	1
0	1	0	4	x
0	1	0	5	x
0	1	1	0	1
0	1	1	1	1
0	1	1	2	1
0	1	1	3	1
0	1	1	4	x
0	1	1	5	x
0	1	2	0	1
0	1	2	1	1
0	1	2	2	1
0	1	2	3	1
0	1	2	4	x
0	1	2	5	x
0	1	3	0	1
0	1	3	1	1
0	1	3	2	1
0	1	3	3	1
0	1	3	4	x
0	1	3	5	x
0	1	4	0	1
0	1	4	1	1
0	1	4	2	1
0	1	4	3	1
0	1	4	4	x
0	1	4	5	x
0	1	5	0	1
0	1	5	1	1
0	1	5	2	1
0	1	5	3	1
0	1	5	4	x
0	1	5	5	x
0	2	0	0	1
0	2	0	1	1
0	2	0	2	1
0	2	0	3	1
0	2	0	4	x
0	2	0	5	x
0	2	1	0	1
0	2	1	1	1
0	2	1	2	1
0	2	1	3	1

0	2	1	4	x
0	2	1	5	x
0	2	2	0	1
0	2	2	1	1
0	2	2	2	1
0	2	2	3	1
0	2	2	4	x
0	2	2	5	x
0	2	3	0	1
0	2	3	1	1
0	2	3	2	1
0	2	3	3	1
0	2	3	4	x
0	2	3	5	x
0	2	4	0	1
0	2	4	1	1
0	2	4	2	1
0	2	4	3	1
0	2	4	4	x
0	2	4	5	x
0	2	5	0	1
0	2	5	1	1
0	2	5	2	1
0	2	5	3	1
0	2	5	4	x
0	2	5	5	x
0	3	0	0	1
0	3	0	1	1
0	3	0	2	1
0	3	0	3	1
0	3	0	4	x
0	3	0	5	x
0	3	1	0	1
0	3	1	1	1
0	3	1	2	1
0	3	1	3	1
0	3	1	4	x
0	3	1	5	x
0	3	2	0	1
0	3	2	1	1
0	3	2	2	1
0	3	2	3	1
0	3	2	4	x
0	3	2	5	x
0	3	3	0	1
0	3	3	1	1
0	3	3	2	1
0	3	3	3	1

0	3	3	4	x
0	3	3	5	x
0	3	4	0	1
0	3	4	1	1
0	3	4	2	1
0	3	4	3	1
0	3	4	4	x
0	3	4	5	x
0	3	5	0	1
0	3	5	1	1
0	3	5	2	1
0	3	5	3	1
0	3	5	4	x
0	3	5	5	x
0	4	0	0	x
0	4	0	1	x
0	4	0	2	x
0	4	0	3	x
0	4	0	4	x
0	4	0	5	x
0	4	1	0	x
0	4	1	1	x
0	4	1	2	x
0	4	1	3	x
0	4	1	4	x
0	4	1	5	x
0	4	2	0	x
0	4	2	1	x
0	4	2	2	x
0	4	2	3	x
0	4	2	4	x
0	4	2	5	x
0	4	3	0	x
0	4	3	1	x
0	4	3	2	x
0	4	3	3	x
0	4	3	4	x
0	4	3	5	x
0	4	4	0	x
0	4	4	1	x
0	4	4	2	x
0	4	4	3	x
0	4	4	4	x
0	4	4	5	x
0	4	5	0	x
0	4	5	1	x
0	4	5	2	x
0	4	5	3	x

0	4	5	4	x
0	4	5	5	x
0	5	0	0	x
0	5	0	1	x
0	5	0	2	x
0	5	0	3	x
0	5	0	4	x
0	5	0	5	x
0	5	1	0	x
0	5	1	1	x
0	5	1	2	x
0	5	1	3	x
0	5	1	4	x
0	5	1	5	x
0	5	2	0	x
0	5	2	1	x
0	5	2	2	x
0	5	2	3	x
0	5	2	4	x
0	5	2	5	x
0	5	3	0	x
0	5	3	1	x
0	5	3	2	x
0	5	3	3	x
0	5	3	4	x
0	5	3	5	x
0	5	4	0	x
0	5	4	1	x
0	5	4	2	x
0	5	4	3	x
0	5	4	4	x
0	5	4	5	x
0	5	5	0	x
0	5	5	1	x
0	5	5	2	x
0	5	5	3	x
0	5	5	4	x
0	5	5	5	x
1	0	0	0	0
1	0	0	1	0
1	0	0	2	1
1	0	0	3	1
1	0	0	4	x
1	0	0	5	x
1	0	1	0	0
1	0	1	1	0
1	0	1	2	1
1	0	1	3	1

1	0	1	4	x
1	0	1	5	x
1	0	2	0	0
1	0	2	1	0
1	0	2	2	1
1	0	2	3	1
1	0	2	4	x
1	0	2	5	x
1	0	3	0	1
1	0	3	1	1
1	0	3	2	1
1	0	3	3	1
1	0	3	4	x
1	0	3	5	x
1	0	4	0	1
1	0	4	1	1
1	0	4	2	1
1	0	4	3	1
1	0	4	4	x
1	0	4	5	x
1	0	5	0	1
1	0	5	1	1
1	0	5	2	1
1	0	5	3	1
1	0	5	4	x
1	0	5	5	x
1	1	0	0	0
1	1	0	1	0
1	1	0	2	1
1	1	0	3	1
1	1	0	4	x
1	1	0	5	x
1	1	1	0	0
1	1	1	1	0
1	1	1	2	1
1	1	1	3	1
1	1	1	4	x
1	1	1	5	x
1	1	2	0	0
1	1	2	1	1
1	1	2	2	1
1	1	2	3	1
1	1	2	4	x
1	1	2	5	x
1	1	3	0	1
1	1	3	1	1
1	1	3	2	1
1	1	3	3	1

1	1	3	4	x
1	1	3	5	x
1	1	4	0	1
1	1	4	1	1
1	1	4	2	1
1	1	4	3	1
1	1	4	4	x
1	1	4	5	x
1	1	5	0	1
1	1	5	1	1
1	1	5	2	1
1	1	5	3	1
1	1	5	4	x
1	1	5	5	x
1	2	0	0	0
1	2	0	1	0
1	2	0	2	0
1	2	0	3	1
1	2	0	4	x
1	2	0	5	x
1	2	1	0	0
1	2	1	1	0
1	2	1	2	1
1	2	1	3	1
1	2	1	4	x
1	2	1	5	x
1	2	2	0	0
1	2	2	1	1
1	2	2	2	1
1	2	2	3	1
1	2	2	4	x
1	2	2	5	x
1	2	3	0	1
1	2	3	1	1
1	2	3	2	1
1	2	3	3	1
1	2	3	4	x
1	2	3	5	x
1	2	4	0	1
1	2	4	1	1
1	2	4	2	1
1	2	4	3	1
1	2	4	4	x
1	2	4	5	x
1	2	5	0	1
1	2	5	1	1
1	2	5	2	1
1	2	5	3	1

1	2	5	4	x
1	2	5	5	x
1	3	0	0	1
1	3	0	1	1
1	3	0	2	1
1	3	0	3	1
1	3	0	4	x
1	3	0	5	x
1	3	1	0	1
1	3	1	1	1
1	3	1	2	1
1	3	1	3	1
1	3	1	4	x
1	3	1	5	x
1	3	2	0	1
1	3	2	1	1
1	3	2	2	1
1	3	2	3	1
1	3	2	4	x
1	3	2	5	x
1	3	3	0	1
1	3	3	1	1
1	3	3	2	1
1	3	3	3	1
1	3	3	4	x
1	3	3	5	x
1	3	4	0	1
1	3	4	1	1
1	3	4	2	1
1	3	4	3	1
1	3	4	4	x
1	3	4	5	x
1	3	5	0	1
1	3	5	1	1
1	3	5	2	1
1	3	5	3	1
1	3	5	4	x
1	3	5	5	x
1	4	0	0	x
1	4	0	1	x
1	4	0	2	x
1	4	0	3	x
1	4	0	4	x
1	4	0	5	x
1	4	1	0	x
1	4	1	1	x
1	4	1	2	x
1	4	1	3	x

1	4	1	4	x
1	4	1	5	x
1	4	2	0	x
1	4	2	1	x
1	4	2	2	x
1	4	2	3	x
1	4	2	4	x
1	4	2	5	x
1	4	3	0	x
1	4	3	1	x
1	4	3	2	x
1	4	3	3	x
1	4	3	4	x
1	4	3	5	x
1	4	4	0	x
1	4	4	1	x
1	4	4	2	x
1	4	4	3	x
1	4	4	4	x
1	4	4	5	x
1	4	5	0	x
1	4	5	1	x
1	4	5	2	x
1	4	5	3	x
1	4	5	4	x
1	4	5	5	x
1	5	0	0	x
1	5	0	1	x
1	5	0	2	x
1	5	0	3	x
1	5	0	4	x
1	5	0	5	x
1	5	1	0	x
1	5	1	1	x
1	5	1	2	x
1	5	1	3	x
1	5	1	4	x
1	5	1	5	x
1	5	2	0	x
1	5	2	1	x
1	5	2	2	x
1	5	2	3	x
1	5	2	4	x
1	5	2	5	x
1	5	3	0	x
1	5	3	1	x
1	5	3	2	x
1	5	3	3	x

1	5	3	4	x
1	5	3	5	x
1	5	4	0	x
1	5	4	1	x
1	5	4	2	x
1	5	4	3	x
1	5	4	4	x
1	5	4	5	x
1	5	5	0	x
1	5	5	1	x
1	5	5	2	x
1	5	5	3	x
1	5	5	4	x
1	5	5	5	x
2	0	0	0	x
2	0	0	1	x
2	0	0	2	x
2	0	0	3	x
2	0	0	4	x
2	0	0	5	x
2	0	1	0	x
2	0	1	1	x
2	0	1	2	x
2	0	1	3	x
2	0	1	4	x
2	0	1	5	x
2	0	2	0	x
2	0	2	1	x
2	0	2	2	x
2	0	2	3	x
2	0	2	4	x
2	0	2	5	x
2	0	3	0	x
2	0	3	1	x
2	0	3	2	x
2	0	3	3	x
2	0	3	4	x
2	0	3	5	x
2	0	4	0	x
2	0	4	1	x
2	0	4	2	x
2	0	4	3	x
2	0	4	4	x
2	0	4	5	x
2	0	5	0	x
2	0	5	1	x
2	0	5	2	x
2	0	5	3	x

2	0	5	4	x
2	0	5	5	x
2	1	0	0	x
2	1	0	1	x
2	1	0	2	x
2	1	0	3	x
2	1	0	4	x
2	1	0	5	x
2	1	1	0	x
2	1	1	1	x
2	1	1	2	x
2	1	1	3	x
2	1	1	4	x
2	1	1	5	x
2	1	2	0	x
2	1	2	1	x
2	1	2	2	x
2	1	2	3	x
2	1	2	4	x
2	1	2	5	x
2	1	3	0	x
2	1	3	1	x
2	1	3	2	x
2	1	3	3	x
2	1	3	4	x
2	1	3	5	x
2	1	4	0	x
2	1	4	1	x
2	1	4	2	x
2	1	4	3	x
2	1	4	4	x
2	1	4	5	x
2	1	5	0	x
2	1	5	1	x
2	1	5	2	x
2	1	5	3	x
2	1	5	4	x
2	1	5	5	x
2	2	0	0	x
2	2	0	1	x
2	2	0	2	x
2	2	0	3	x
2	2	0	4	x
2	2	0	5	x
2	2	1	0	x
2	2	1	1	x
2	2	1	2	x
2	2	1	3	x

2	2	1	4	x
2	2	1	5	x
2	2	2	0	x
2	2	2	1	x
2	2	2	2	x
2	2	2	3	x
2	2	2	4	x
2	2	2	5	x
2	2	3	0	x
2	2	3	1	x
2	2	3	2	x
2	2	3	3	x
2	2	3	4	x
2	2	3	5	x
2	2	4	0	x
2	2	4	1	x
2	2	4	2	x
2	2	4	3	x
2	2	4	4	x
2	2	4	5	x
2	2	5	0	x
2	2	5	1	x
2	2	5	2	x
2	2	5	3	x
2	2	5	4	x
2	2	5	5	x
2	3	0	0	x
2	3	0	1	x
2	3	0	2	x
2	3	0	3	x
2	3	0	4	x
2	3	0	5	x
2	3	1	0	x
2	3	1	1	x
2	3	1	2	x
2	3	1	3	x
2	3	1	4	x
2	3	1	5	x
2	3	2	0	x
2	3	2	1	x
2	3	2	2	x
2	3	2	3	x
2	3	2	4	x
2	3	2	5	x
2	3	3	0	x
2	3	3	1	x
2	3	3	2	x
2	3	3	3	x

2	3	3	4	x
2	3	3	5	x
2	3	4	0	x
2	3	4	1	x
2	3	4	2	x
2	3	4	3	x
2	3	4	4	x
2	3	4	5	x
2	3	5	0	x
2	3	5	1	x
2	3	5	2	x
2	3	5	3	x
2	3	5	4	x
2	3	5	5	x
2	4	0	0	x
2	4	0	1	x
2	4	0	2	x
2	4	0	3	x
2	4	0	4	x
2	4	0	5	x
2	4	1	0	x
2	4	1	1	x
2	4	1	2	x
2	4	1	3	x
2	4	1	4	x
2	4	1	5	x
2	4	2	0	x
2	4	2	1	x
2	4	2	2	x
2	4	2	3	x
2	4	2	4	x
2	4	2	5	x
2	4	3	0	x
2	4	3	1	x
2	4	3	2	x
2	4	3	3	x
2	4	3	4	x
2	4	3	5	x
2	4	4	0	x
2	4	4	1	x
2	4	4	2	x
2	4	4	3	x
2	4	4	4	x
2	4	4	5	x
2	4	5	0	x
2	4	5	1	x
2	4	5	2	x
2	4	5	3	x

2	4	5	4	x
2	4	5	5	x
2	5	0	0	x
2	5	0	1	x
2	5	0	2	x
2	5	0	3	x
2	5	0	4	x
2	5	0	5	x
2	5	1	0	x
2	5	1	1	x
2	5	1	2	x
2	5	1	3	x
2	5	1	4	x
2	5	1	5	x
2	5	2	0	x
2	5	2	1	x
2	5	2	2	x
2	5	2	3	x
2	5	2	4	x
2	5	2	5	x
2	5	3	0	x
2	5	3	1	x
2	5	3	2	x
2	5	3	3	x
2	5	3	4	x
2	5	3	5	x
2	5	4	0	x
2	5	4	1	x
2	5	4	2	x
2	5	4	3	x
2	5	4	4	x
2	5	4	5	x
2	5	5	0	x
2	5	5	1	x
2	5	5	2	x
2	5	5	3	x
2	5	5	4	x
2	5	5	5	x
3	0	0	0	x
3	0	0	1	x
3	0	0	2	x
3	0	0	3	x
3	0	0	4	x
3	0	0	5	x
3	0	1	0	x
3	0	1	1	x
3	0	1	2	x
3	0	1	3	x

3	0	1	4	x
3	0	1	5	x
3	0	2	0	x
3	0	2	1	x
3	0	2	2	x
3	0	2	3	x
3	0	2	4	x
3	0	2	5	x
3	0	3	0	x
3	0	3	1	x
3	0	3	2	x
3	0	3	3	x
3	0	3	4	x
3	0	3	5	x
3	0	4	0	x
3	0	4	1	x
3	0	4	2	x
3	0	4	3	x
3	0	4	4	x
3	0	4	5	x
3	0	5	0	x
3	0	5	1	x
3	0	5	2	x
3	0	5	3	x
3	0	5	4	x
3	0	5	5	x
3	1	0	0	x
3	1	0	1	x
3	1	0	2	x
3	1	0	3	x
3	1	0	4	x
3	1	0	5	x
3	1	1	0	x
3	1	1	1	x
3	1	1	2	x
3	1	1	3	x
3	1	1	4	x
3	1	1	5	x
3	1	2	0	x
3	1	2	1	x
3	1	2	2	x
3	1	2	3	x
3	1	2	4	x
3	1	2	5	x
3	1	3	0	x
3	1	3	1	x
3	1	3	2	x
3	1	3	3	x

Complex regulation of Hsf1-Skn7 activities by the catalytic subunits of PKA in Saccharomyces cerevisiae: experimental and computational approaches.

3	1	3	4	x
3	1	3	5	x
3	1	4	0	x
3	1	4	1	x
3	1	4	2	x
3	1	4	3	x
3	1	4	4	x
3	1	4	5	x
3	1	5	0	x
3	1	5	1	x
3	1	5	2	x
3	1	5	3	x
3	1	5	4	x
3	1	5	5	x
3	2	0	0	x
3	2	0	1	x
3	2	0	2	x
3	2	0	3	x
3	2	0	4	x
3	2	0	5	x
3	2	1	0	x
3	2	1	1	x
3	2	1	2	x
3	2	1	3	x
3	2	1	4	x
3	2	1	5	x
3	2	2	0	x
3	2	2	1	x
3	2	2	2	x
3	2	2	3	x
3	2	2	4	x
3	2	2	5	x
3	2	3	0	x
3	2	3	1	x
3	2	3	2	x
3	2	3	3	x
3	2	3	4	x
3	2	3	5	x
3	2	4	0	x
3	2	4	1	x
3	2	4	2	x
3	2	4	3	x
3	2	4	4	x
3	2	4	5	x
3	2	5	0	x
3	2	5	1	x
3	2	5	2	x
3	2	5	3	x

3	2	5	4	x
3	2	5	5	x
3	3	0	0	x
3	3	0	1	x
3	3	0	2	x
3	3	0	3	x
3	3	0	4	x
3	3	0	5	x
3	3	1	0	x
3	3	1	1	x
3	3	1	2	x
3	3	1	3	x
3	3	1	4	x
3	3	1	5	x
3	3	2	0	x
3	3	2	1	x
3	3	2	2	x
3	3	2	3	x
3	3	2	4	x
3	3	2	5	x
3	3	3	0	x
3	3	3	1	x
3	3	3	2	x
3	3	3	3	x
3	3	3	4	x
3	3	3	5	x
3	3	4	0	x
3	3	4	1	x
3	3	4	2	x
3	3	4	3	x
3	3	4	4	x
3	3	4	5	x
3	3	5	0	x
3	3	5	1	x
3	3	5	2	x
3	3	5	3	x
3	3	5	4	x
3	3	5	5	x
3	4	0	0	x
3	4	0	1	x
3	4	0	2	x
3	4	0	3	x
3	4	0	4	x
3	4	0	5	x
3	4	1	0	x
3	4	1	1	x
3	4	1	2	x
3	4	1	3	x

3	4	1	4	x
3	4	1	5	x
3	4	2	0	x
3	4	2	1	x
3	4	2	2	x
3	4	2	3	x
3	4	2	4	x
3	4	2	5	x
3	4	3	0	x
3	4	3	1	x
3	4	3	2	x
3	4	3	3	x
3	4	3	4	x
3	4	3	5	x
3	4	4	0	x
3	4	4	1	x
3	4	4	2	x
3	4	4	3	x
3	4	4	4	x
3	4	4	5	x
3	4	5	0	x
3	4	5	1	x
3	4	5	2	x
3	4	5	3	x
3	4	5	4	x
3	4	5	5	x
3	5	0	0	x
3	5	0	1	x
3	5	0	2	x
3	5	0	3	x
3	5	0	4	x
3	5	0	5	x
3	5	1	0	x
3	5	1	1	x
3	5	1	2	x
3	5	1	3	x
3	5	1	4	x
3	5	1	5	x
3	5	2	0	x
3	5	2	1	x
3	5	2	2	x
3	5	2	3	x
3	5	2	4	x
3	5	2	5	x
3	5	3	0	x
3	5	3	1	x
3	5	3	2	x
3	5	3	3	x

3	5	3	4	x
3	5	3	5	x
3	5	4	0	x
3	5	4	1	x
3	5	4	2	x
3	5	4	3	x
3	5	4	4	x
3	5	4	5	x
3	5	5	0	x
3	5	5	1	x
3	5	5	2	x
3	5	5	3	x
3	5	5	4	x
3	5	5	5	x
4	0	0	0	x
4	0	0	1	x
4	0	0	2	x
4	0	0	3	x
4	0	0	4	x
4	0	0	5	x
4	0	1	0	x
4	0	1	1	x
4	0	1	2	x
4	0	1	3	x
4	0	1	4	x
4	0	1	5	x
4	0	2	0	x
4	0	2	1	x
4	0	2	2	x
4	0	2	3	x
4	0	2	4	x
4	0	2	5	x
4	0	3	0	x
4	0	3	1	x
4	0	3	2	x
4	0	3	3	x
4	0	3	4	x
4	0	3	5	x
4	0	4	0	x
4	0	4	1	x
4	0	4	2	x
4	0	4	3	x
4	0	4	4	x
4	0	4	5	x
4	0	5	0	x
4	0	5	1	x
4	0	5	2	x
4	0	5	3	x

4	0	5	4	x
4	0	5	5	x
4	1	0	0	x
4	1	0	1	x
4	1	0	2	x
4	1	0	3	x
4	1	0	4	x
4	1	0	5	x
4	1	1	0	x
4	1	1	1	x
4	1	1	2	x
4	1	1	3	x
4	1	1	4	x
4	1	1	5	x
4	1	2	0	x
4	1	2	1	x
4	1	2	2	x
4	1	2	3	x
4	1	2	4	x
4	1	2	5	x
4	1	3	0	x
4	1	3	1	x
4	1	3	2	x
4	1	3	3	x
4	1	3	4	x
4	1	3	5	x
4	1	4	0	x
4	1	4	1	x
4	1	4	2	x
4	1	4	3	x
4	1	4	4	x
4	1	4	5	x
4	1	5	0	x
4	1	5	1	x
4	1	5	2	x
4	1	5	3	x
4	1	5	4	x
4	1	5	5	x
4	2	0	0	x
4	2	0	1	x
4	2	0	2	x
4	2	0	3	x
4	2	0	4	x
4	2	0	5	x
4	2	1	0	x
4	2	1	1	x
4	2	1	2	x
4	2	1	3	x

4	2	1	4	x
4	2	1	5	x
4	2	2	0	x
4	2	2	1	x
4	2	2	2	x
4	2	2	3	x
4	2	2	4	x
4	2	2	5	x
4	2	3	0	x
4	2	3	1	x
4	2	3	2	x
4	2	3	3	x
4	2	3	4	x
4	2	3	5	x
4	2	4	0	x
4	2	4	1	x
4	2	4	2	x
4	2	4	3	x
4	2	4	4	x
4	2	4	5	x
4	2	5	0	x
4	2	5	1	x
4	2	5	2	x
4	2	5	3	x
4	2	5	4	x
4	2	5	5	x
4	3	0	0	x
4	3	0	1	x
4	3	0	2	x
4	3	0	3	x
4	3	0	4	x
4	3	0	5	x
4	3	1	0	x
4	3	1	1	x
4	3	1	2	x
4	3	1	3	x
4	3	1	4	x
4	3	1	5	x
4	3	2	0	x
4	3	2	1	x
4	3	2	2	x
4	3	2	3	x
4	3	2	4	x
4	3	2	5	x
4	3	3	0	x
4	3	3	1	x
4	3	3	2	x
4	3	3	3	x

4	3	3	4	x
4	3	3	5	x
4	3	4	0	x
4	3	4	1	x
4	3	4	2	x
4	3	4	3	x
4	3	4	4	x
4	3	4	5	x
4	3	5	0	x
4	3	5	1	x
4	3	5	2	x
4	3	5	3	x
4	3	5	4	x
4	3	5	5	x
4	4	0	0	x
4	4	0	1	x
4	4	0	2	x
4	4	0	3	x
4	4	0	4	x
4	4	0	5	x
4	4	1	0	x
4	4	1	1	x
4	4	1	2	x
4	4	1	3	x
4	4	1	4	x
4	4	1	5	x
4	4	2	0	x
4	4	2	1	x
4	4	2	2	x
4	4	2	3	x
4	4	2	4	x
4	4	2	5	x
4	4	3	0	x
4	4	3	1	x
4	4	3	2	x
4	4	3	3	x
4	4	3	4	x
4	4	3	5	x
4	4	4	0	x
4	4	4	1	x
4	4	4	2	x
4	4	4	3	x
4	4	4	4	x
4	4	4	5	x
4	4	5	0	x
4	4	5	1	x
4	4	5	2	x
4	4	5	3	x

4	4	5	4	x
4	4	5	5	x
4	5	0	0	x
4	5	0	1	x
4	5	0	2	x
4	5	0	3	x
4	5	0	4	x
4	5	0	5	x
4	5	1	0	x
4	5	1	1	x
4	5	1	2	x
4	5	1	3	x
4	5	1	4	x
4	5	1	5	x
4	5	2	0	x
4	5	2	1	x
4	5	2	2	x
4	5	2	3	x
4	5	2	4	x
4	5	2	5	x
4	5	3	0	x
4	5	3	1	x
4	5	3	2	x
4	5	3	3	x
4	5	3	4	x
4	5	3	5	x
4	5	4	0	x
4	5	4	1	x
4	5	4	2	x
4	5	4	3	x
4	5	4	4	x
4	5	4	5	x
4	5	5	0	x
4	5	5	1	x
4	5	5	2	x
4	5	5	3	x
4	5	5	4	x
4	5	5	5	x
5	0	0	0	x
5	0	0	1	x
5	0	0	2	x
5	0	0	3	x
5	0	0	4	x
5	0	0	5	x
5	0	1	0	x
5	0	1	1	x
5	0	1	2	x
5	0	1	3	x

5	0	1	4	x
5	0	1	5	x
5	0	2	0	x
5	0	2	1	x
5	0	2	2	x
5	0	2	3	x
5	0	2	4	x
5	0	2	5	x
5	0	3	0	x
5	0	3	1	x
5	0	3	2	x
5	0	3	3	x
5	0	3	4	x
5	0	3	5	x
5	0	4	0	x
5	0	4	1	x
5	0	4	2	x
5	0	4	3	x
5	0	4	4	x
5	0	4	5	x
5	0	5	0	x
5	0	5	1	x
5	0	5	2	x
5	0	5	3	x
5	0	5	4	x
5	0	5	5	x
5	1	0	0	x
5	1	0	1	x
5	1	0	2	x
5	1	0	3	x
5	1	0	4	x
5	1	0	5	x
5	1	1	0	x
5	1	1	1	x
5	1	1	2	x
5	1	1	3	x
5	1	1	4	x
5	1	1	5	x
5	1	2	0	x
5	1	2	1	x
5	1	2	2	x
5	1	2	3	x
5	1	2	4	x
5	1	2	5	x
5	1	3	0	x
5	1	3	1	x
5	1	3	2	x
5	1	3	3	x

5	1	3	4	x
5	1	3	5	x
5	1	4	0	x
5	1	4	1	x
5	1	4	2	x
5	1	4	3	x
5	1	4	4	x
5	1	4	5	x
5	1	5	0	x
5	1	5	1	x
5	1	5	2	x
5	1	5	3	x
5	1	5	4	x
5	1	5	5	x
5	2	0	0	x
5	2	0	1	x
5	2	0	2	x
5	2	0	3	x
5	2	0	4	x
5	2	0	5	x
5	2	1	0	x
5	2	1	1	x
5	2	1	2	x
5	2	1	3	x
5	2	1	4	x
5	2	1	5	x
5	2	2	0	x
5	2	2	1	x
5	2	2	2	x
5	2	2	3	x
5	2	2	4	x
5	2	2	5	x
5	2	3	0	x
5	2	3	1	x
5	2	3	2	x
5	2	3	3	x
5	2	3	4	x
5	2	3	5	x
5	2	4	0	x
5	2	4	1	x
5	2	4	2	x
5	2	4	3	x
5	2	4	4	x
5	2	4	5	x
5	2	5	0	x
5	2	5	1	x
5	2	5	2	x
5	2	5	3	x

5	2	5	4	x
5	2	5	5	x
5	3	0	0	x
5	3	0	1	x
5	3	0	2	x
5	3	0	3	x
5	3	0	4	x
5	3	0	5	x
5	3	1	0	x
5	3	1	1	x
5	3	1	2	x
5	3	1	3	x
5	3	1	4	x
5	3	1	5	x
5	3	2	0	x
5	3	2	1	x
5	3	2	2	x
5	3	2	3	x
5	3	2	4	x
5	3	2	5	x
5	3	3	0	x
5	3	3	1	x
5	3	3	2	x
5	3	3	3	x
5	3	3	4	x
5	3	3	5	x
5	3	4	0	x
5	3	4	1	x
5	3	4	2	x
5	3	4	3	x
5	3	4	4	x
5	3	4	5	x
5	3	5	0	x
5	3	5	1	x
5	3	5	2	x
5	3	5	3	x
5	3	5	4	x
5	3	5	5	x
5	4	0	0	x
5	4	0	1	x
5	4	0	2	x
5	4	0	3	x
5	4	0	4	x
5	4	0	5	x
5	4	1	0	x
5	4	1	1	x
5	4	1	2	x
5	4	1	3	x

5	4	1	4	x
5	4	1	5	x
5	4	2	0	x
5	4	2	1	x
5	4	2	2	x
5	4	2	3	x
5	4	2	4	x
5	4	2	5	x
5	4	3	0	x
5	4	3	1	x
5	4	3	2	x
5	4	3	3	x
5	4	3	4	x
5	4	3	5	x
5	4	4	0	x
5	4	4	1	x
5	4	4	2	x
5	4	4	3	x
5	4	4	4	x
5	4	4	5	x
5	4	5	0	x
5	4	5	1	x
5	4	5	2	x
5	4	5	3	x
5	4	5	4	x
5	4	5	5	x
5	5	0	0	x
5	5	0	1	x
5	5	0	2	x
5	5	0	3	x
5	5	0	4	x
5	5	0	5	x
5	5	1	0	x
5	5	1	1	x
5	5	1	2	x
5	5	1	3	x
5	5	1	4	x
5	5	1	5	x
5	5	2	0	x
5	5	2	1	x
5	5	2	2	x
5	5	2	3	x
5	5	2	4	x
5	5	2	5	x
5	5	3	0	x
5	5	3	1	x
5	5	3	2	x
5	5	3	3	x

5	5	3	4	x
5	5	3	5	x
5	5	4	0	x
5	5	4	1	x
5	5	4	2	x
5	5	4	3	x
5	5	4	4	x
5	5	4	5	x
5	5	5	0	x
5	5	5	1	x
5	5	5	2	x
5	5	5	3	x
5	5	5	4	x
5	5	5	5	x

**Table SI12.** Ssa2 node.				
Table entries: 1296; references: 15, 16, 17.				
**Heat**	**Tpk1***	**Tpk2**	**Tpk3***	**<<Ssa2>> (true)**
0	0	0	0	0
0	0	0	1	1
0	0	0	2	2
0	0	0	3	3
0	0	0	4	x
0	0	0	5	x
0	0	1	0	1
0	0	1	1	2
0	0	1	2	3
0	0	1	3	4
0	0	1	4	x
0	0	1	5	x
0	0	2	0	2
0	0	2	1	2
0	0	2	2	3
0	0	2	3	3
0	0	2	4	x
0	0	2	5	x
0	0	3	0	2
0	0	3	1	2
0	0	3	2	3
0	0	3	3	4
0	0	3	4	x
0	0	3	5	x
0	0	4	0	3
0	0	4	1	3
0	0	4	2	3
0	0	4	3	4
0	0	4	4	x
0	0	4	5	x

0	0	5	0	4
0	0	5	1	4
0	0	5	2	4
0	0	5	3	4
0	0	5	4	x
0	0	5	5	x
0	1	0	0	2
0	1	0	1	2
0	1	0	2	2
0	1	0	3	3
0	1	0	4	x
0	1	0	5	x
0	1	1	0	1
0	1	1	1	2
0	1	1	2	2
0	1	1	3	3
0	1	1	4	x
0	1	1	5	x
0	1	2	0	2
0	1	2	1	2
0	1	2	2	3
0	1	2	3	4
0	1	2	4	x
0	1	2	5	x
0	1	3	0	2
0	1	3	1	3
0	1	3	2	4
0	1	3	3	4
0	1	3	4	x
0	1	3	5	x
0	1	4	0	3
0	1	4	1	3
0	1	4	2	4
0	1	4	3	4
0	1	4	4	x
0	1	4	5	x
0	1	5	0	4
0	1	5	1	4
0	1	5	2	4
0	1	5	3	4
0	1	5	4	x
0	1	5	5	x
0	2	0	0	3
0	2	0	1	3
0	2	0	2	3
0	2	0	3	4
0	2	0	4	x
0	2	0	5	x

0	2	1	0	2
0	2	1	1	2
0	2	1	2	3
0	2	1	3	3
0	2	1	4	x
0	2	1	5	x
0	2	2	0	3
0	2	2	1	3
0	2	2	2	3
0	2	2	3	4
0	2	2	4	x
0	2	2	5	x
0	2	3	0	3
0	2	3	1	3
0	2	3	2	3
0	2	3	3	4
0	2	3	4	x
0	2	3	5	x
0	2	4	0	3
0	2	4	1	3
0	2	4	2	3
0	2	4	3	4
0	2	4	4	x
0	2	4	5	x
0	2	5	0	4
0	2	5	1	4
0	2	5	2	4
0	2	5	3	4
0	2	5	4	x
0	2	5	5	x
0	3	0	0	4
0	3	0	1	4
0	3	0	2	4
0	3	0	3	4
0	3	0	4	x
0	3	0	5	x
0	3	1	0	4
0	3	1	1	4
0	3	1	2	4
0	3	1	3	4
0	3	1	4	x
0	3	1	5	x
0	3	2	0	4
0	3	2	1	4
0	3	2	2	4
0	3	2	3	4
0	3	2	4	x
0	3	2	5	x

0	3	3	0	4
0	3	3	1	4
0	3	3	2	4
0	3	3	3	4
0	3	3	4	x
0	3	3	5	x
0	3	4	0	4
0	3	4	1	4
0	3	4	2	4
0	3	4	3	4
0	3	4	4	x
0	3	4	5	x
0	3	5	0	4
0	3	5	1	4
0	3	5	2	4
0	3	5	3	4
0	3	5	4	x
0	3	5	5	x
0	4	0	0	x
0	4	0	1	x
0	4	0	2	x
0	4	0	3	x
0	4	0	4	x
0	4	0	5	x
0	4	1	0	x
0	4	1	1	x
0	4	1	2	x
0	4	1	3	x
0	4	1	4	x
0	4	1	5	x
0	4	2	0	x
0	4	2	1	x
0	4	2	2	x
0	4	2	3	x
0	4	2	4	x
0	4	2	5	x
0	4	3	0	x
0	4	3	1	x
0	4	3	2	x
0	4	3	3	x
0	4	3	4	x
0	4	3	5	x
0	4	4	0	x
0	4	4	1	x
0	4	4	2	x
0	4	4	3	x
0	4	4	4	x
0	4	4	5	x

0	4	5	0	x
0	4	5	1	x
0	4	5	2	x
0	4	5	3	x
0	4	5	4	x
0	4	5	5	x
0	5	0	0	x
0	5	0	1	x
0	5	0	2	x
0	5	0	3	x
0	5	0	4	x
0	5	0	5	x
0	5	1	0	x
0	5	1	1	x
0	5	1	2	x
0	5	1	3	x
0	5	1	4	x
0	5	1	5	x
0	5	2	0	x
0	5	2	1	x
0	5	2	2	x
0	5	2	3	x
0	5	2	4	x
0	5	2	5	x
0	5	3	0	x
0	5	3	1	x
0	5	3	2	x
0	5	3	3	x
0	5	3	4	x
0	5	3	5	x
0	5	4	0	x
0	5	4	1	x
0	5	4	2	x
0	5	4	3	x
0	5	4	4	x
0	5	4	5	x
0	5	5	0	x
0	5	5	1	x
0	5	5	2	x
0	5	5	3	x
0	5	5	4	x
0	5	5	5	x
1	0	0	0	0
1	0	0	1	1
1	0	0	2	1
1	0	0	3	2
1	0	0	4	x
1	0	0	5	x

1	0	1	0	0
1	0	1	1	1
1	0	1	2	2
1	0	1	3	2
1	0	1	4	x
1	0	1	5	x
1	0	2	0	1
1	0	2	1	1
1	0	2	2	2
1	0	2	3	2
1	0	2	4	x
1	0	2	5	x
1	0	3	0	1
1	0	3	1	1
1	0	3	2	2
1	0	3	3	3
1	0	3	4	x
1	0	3	5	x
1	0	4	0	2
1	0	4	1	2
1	0	4	2	2
1	0	4	3	3
1	0	4	4	x
1	0	4	5	x
1	0	5	0	3
1	0	5	1	3
1	0	5	2	3
1	0	5	3	3
1	0	5	4	x
1	0	5	5	x
1	1	0	0	1
1	1	0	1	1
1	1	0	2	1
1	1	0	3	2
1	1	0	4	x
1	1	0	5	x
1	1	1	0	0
1	1	1	1	1
1	1	1	2	1
1	1	1	3	2
1	1	1	4	x
1	1	1	5	x
1	1	2	0	1
1	1	2	1	1
1	1	2	2	2
1	1	2	3	3
1	1	2	4	x
1	1	2	5	x

Complex regulation of Hsf1-Skn7 activities by the catalytic subunits of PKA in Saccharomyces cerevisiae: experimental and computational approaches.

1	1	3	0	1
1	1	3	1	2
1	1	3	2	3
1	1	3	3	3
1	1	3	4	x
1	1	3	5	x
1	1	4	0	2
1	1	4	1	2
1	1	4	2	3
1	1	4	3	3
1	1	4	4	x
1	1	4	5	x
1	1	5	0	3
1	1	5	1	3
1	1	5	2	3
1	1	5	3	3
1	1	5	4	x
1	1	5	5	x
1	2	0	0	1
1	2	0	1	1
1	2	0	2	2
1	2	0	3	2
1	2	0	4	x
1	2	0	5	x
1	2	1	0	1
1	2	1	1	1
1	2	1	2	2
1	2	1	3	2
1	2	1	4	x
1	2	1	5	x
1	2	2	0	2
1	2	2	1	2
1	2	2	2	2
1	2	2	3	3
1	2	2	4	x
1	2	2	5	x
1	2	3	0	2
1	2	3	1	2
1	2	3	2	2
1	2	3	3	3
1	2	3	4	x
1	2	3	5	x
1	2	4	0	2
1	2	4	1	2
1	2	4	2	2
1	2	4	3	3
1	2	4	4	x
1	2	4	5	x

1	2	5	0	3
1	2	5	1	3
1	2	5	2	3
1	2	5	3	3
1	2	5	4	x
1	2	5	5	x
1	3	0	0	3
1	3	0	1	3
1	3	0	2	3
1	3	0	3	3
1	3	0	4	x
1	3	0	5	x
1	3	1	0	3
1	3	1	1	3
1	3	1	2	3
1	3	1	3	3
1	3	1	4	x
1	3	1	5	x
1	3	2	0	3
1	3	2	1	3
1	3	2	2	3
1	3	2	3	3
1	3	2	4	x
1	3	2	5	x
1	3	3	0	3
1	3	3	1	3
1	3	3	2	3
1	3	3	3	3
1	3	3	4	x
1	3	3	5	x
1	3	4	0	3
1	3	4	1	3
1	3	4	2	3
1	3	4	3	3
1	3	4	4	x
1	3	4	5	x
1	3	5	0	3
1	3	5	1	3
1	3	5	2	3
1	3	5	3	3
1	3	5	4	x
1	3	5	5	x
1	4	0	0	x
1	4	0	1	x
1	4	0	2	x
1	4	0	3	x
1	4	0	4	x
1	4	0	5	x

1	4	1	0	x
1	4	1	1	x
1	4	1	2	x
1	4	1	3	x
1	4	1	4	x
1	4	1	5	x
1	4	2	0	x
1	4	2	1	x
1	4	2	2	x
1	4	2	3	x
1	4	2	4	x
1	4	2	5	x
1	4	3	0	x
1	4	3	1	x
1	4	3	2	x
1	4	3	3	x
1	4	3	4	x
1	4	3	5	x
1	4	4	0	x
1	4	4	1	x
1	4	4	2	x
1	4	4	3	x
1	4	4	4	x
1	4	4	5	x
1	4	5	0	x
1	4	5	1	x
1	4	5	2	x
1	4	5	3	x
1	4	5	4	x
1	4	5	5	x
1	5	0	0	x
1	5	0	1	x
1	5	0	2	x
1	5	0	3	x
1	5	0	4	x
1	5	0	5	x
1	5	1	0	x
1	5	1	1	x
1	5	1	2	x
1	5	1	3	x
1	5	1	4	x
1	5	1	5	x
1	5	2	0	x
1	5	2	1	x
1	5	2	2	x
1	5	2	3	x
1	5	2	4	x
1	5	2	5	x

1	5	3	0	x
1	5	3	1	x
1	5	3	2	x
1	5	3	3	x
1	5	3	4	x
1	5	3	5	x
1	5	4	0	x
1	5	4	1	x
1	5	4	2	x
1	5	4	3	x
1	5	4	4	x
1	5	4	5	x
1	5	5	0	x
1	5	5	1	x
1	5	5	2	x
1	5	5	3	x
1	5	5	4	x
1	5	5	5	x
2	0	0	0	x
2	0	0	1	x
2	0	0	2	x
2	0	0	3	x
2	0	0	4	x
2	0	0	5	x
2	0	1	0	x
2	0	1	1	x
2	0	1	2	x
2	0	1	3	x
2	0	1	4	x
2	0	1	5	x
2	0	2	0	x
2	0	2	1	x
2	0	2	2	x
2	0	2	3	x
2	0	2	4	x
2	0	2	5	x
2	0	3	0	x
2	0	3	1	x
2	0	3	2	x
2	0	3	3	x
2	0	3	4	x
2	0	3	5	x
2	0	4	0	x
2	0	4	1	x
2	0	4	2	x
2	0	4	3	x
2	0	4	4	x
2	0	4	5	x

2	0	5	0	x
2	0	5	1	x
2	0	5	2	x
2	0	5	3	x
2	0	5	4	x
2	0	5	5	x
2	1	0	0	x
2	1	0	1	x
2	1	0	2	x
2	1	0	3	x
2	1	0	4	x
2	1	0	5	x
2	1	1	0	x
2	1	1	1	x
2	1	1	2	x
2	1	1	3	x
2	1	1	4	x
2	1	1	5	x
2	1	2	0	x
2	1	2	1	x
2	1	2	2	x
2	1	2	3	x
2	1	2	4	x
2	1	2	5	x
2	1	3	0	x
2	1	3	1	x
2	1	3	2	x
2	1	3	3	x
2	1	3	4	x
2	1	3	5	x
2	1	4	0	x
2	1	4	1	x
2	1	4	2	x
2	1	4	3	x
2	1	4	4	x
2	1	4	5	x
2	1	5	0	x
2	1	5	1	x
2	1	5	2	x
2	1	5	3	x
2	1	5	4	x
2	1	5	5	x
2	2	0	0	x
2	2	0	1	x
2	2	0	2	x
2	2	0	3	x
2	2	0	4	x
2	2	0	5	x

2	2	1	0	x
2	2	1	1	x
2	2	1	2	x
2	2	1	3	x
2	2	1	4	x
2	2	1	5	x
2	2	2	0	x
2	2	2	1	x
2	2	2	2	x
2	2	2	3	x
2	2	2	4	x
2	2	2	5	x
2	2	3	0	x
2	2	3	1	x
2	2	3	2	x
2	2	3	3	x
2	2	3	4	x
2	2	3	5	x
2	2	4	0	x
2	2	4	1	x
2	2	4	2	x
2	2	4	3	x
2	2	4	4	x
2	2	4	5	x
2	2	5	0	x
2	2	5	1	x
2	2	5	2	x
2	2	5	3	x
2	2	5	4	x
2	2	5	5	x
2	3	0	0	x
2	3	0	1	x
2	3	0	2	x
2	3	0	3	x
2	3	0	4	x
2	3	0	5	x
2	3	1	0	x
2	3	1	1	x
2	3	1	2	x
2	3	1	3	x
2	3	1	4	x
2	3	1	5	x
2	3	2	0	x
2	3	2	1	x
2	3	2	2	x
2	3	2	3	x
2	3	2	4	x
2	3	2	5	x

2	3	3	0	x
2	3	3	1	x
2	3	3	2	x
2	3	3	3	x
2	3	3	4	x
2	3	3	5	x
2	3	4	0	x
2	3	4	1	x
2	3	4	2	x
2	3	4	3	x
2	3	4	4	x
2	3	4	5	x
2	3	5	0	x
2	3	5	1	x
2	3	5	2	x
2	3	5	3	x
2	3	5	4	x
2	3	5	5	x
2	4	0	0	x
2	4	0	1	x
2	4	0	2	x
2	4	0	3	x
2	4	0	4	x
2	4	0	5	x
2	4	1	0	x
2	4	1	1	x
2	4	1	2	x
2	4	1	3	x
2	4	1	4	x
2	4	1	5	x
2	4	2	0	x
2	4	2	1	x
2	4	2	2	x
2	4	2	3	x
2	4	2	4	x
2	4	2	5	x
2	4	3	0	x
2	4	3	1	x
2	4	3	2	x
2	4	3	3	x
2	4	3	4	x
2	4	3	5	x
2	4	4	0	x
2	4	4	1	x
2	4	4	2	x
2	4	4	3	x
2	4	4	4	x
2	4	4	5	x

2	4	5	0	x
2	4	5	1	x
2	4	5	2	x
2	4	5	3	x
2	4	5	4	x
2	4	5	5	x
2	5	0	0	x
2	5	0	1	x
2	5	0	2	x
2	5	0	3	x
2	5	0	4	x
2	5	0	5	x
2	5	1	0	x
2	5	1	1	x
2	5	1	2	x
2	5	1	3	x
2	5	1	4	x
2	5	1	5	x
2	5	2	0	x
2	5	2	1	x
2	5	2	2	x
2	5	2	3	x
2	5	2	4	x
2	5	2	5	x
2	5	3	0	x
2	5	3	1	x
2	5	3	2	x
2	5	3	3	x
2	5	3	4	x
2	5	3	5	x
2	5	4	0	x
2	5	4	1	x
2	5	4	2	x
2	5	4	3	x
2	5	4	4	x
2	5	4	5	x
2	5	5	0	x
2	5	5	1	x
2	5	5	2	x
2	5	5	3	x
2	5	5	4	x
2	5	5	5	x
3	0	0	0	x
3	0	0	1	x
3	0	0	2	x
3	0	0	3	x
3	0	0	4	x
3	0	0	5	x

3	0	1	0	x
3	0	1	1	x
3	0	1	2	x
3	0	1	3	x
3	0	1	4	x
3	0	1	5	x
3	0	2	0	x
3	0	2	1	x
3	0	2	2	x
3	0	2	3	x
3	0	2	4	x
3	0	2	5	x
3	0	3	0	x
3	0	3	1	x
3	0	3	2	x
3	0	3	3	x
3	0	3	4	x
3	0	3	5	x
3	0	4	0	x
3	0	4	1	x
3	0	4	2	x
3	0	4	3	x
3	0	4	4	x
3	0	4	5	x
3	0	5	0	x
3	0	5	1	x
3	0	5	2	x
3	0	5	3	x
3	0	5	4	x
3	0	5	5	x
3	1	0	0	x
3	1	0	1	x
3	1	0	2	x
3	1	0	3	x
3	1	0	4	x
3	1	0	5	x
3	1	1	0	x
3	1	1	1	x
3	1	1	2	x
3	1	1	3	x
3	1	1	4	x
3	1	1	5	x
3	1	2	0	x
3	1	2	1	x
3	1	2	2	x
3	1	2	3	x
3	1	2	4	x
3	1	2	5	x

3	1	3	0	x
3	1	3	1	x
3	1	3	2	x
3	1	3	3	x
3	1	3	4	x
3	1	3	5	x
3	1	4	0	x
3	1	4	1	x
3	1	4	2	x
3	1	4	3	x
3	1	4	4	x
3	1	4	5	x
3	1	5	0	x
3	1	5	1	x
3	1	5	2	x
3	1	5	3	x
3	1	5	4	x
3	1	5	5	x
3	2	0	0	x
3	2	0	1	x
3	2	0	2	x
3	2	0	3	x
3	2	0	4	x
3	2	0	5	x
3	2	1	0	x
3	2	1	1	x
3	2	1	2	x
3	2	1	3	x
3	2	1	4	x
3	2	1	5	x
3	2	2	0	x
3	2	2	1	x
3	2	2	2	x
3	2	2	3	x
3	2	2	4	x
3	2	2	5	x
3	2	3	0	x
3	2	3	1	x
3	2	3	2	x
3	2	3	3	x
3	2	3	4	x
3	2	3	5	x
3	2	4	0	x
3	2	4	1	x
3	2	4	2	x
3	2	4	3	x
3	2	4	4	x
3	2	4	5	x

3	2	5	0	x
3	2	5	1	x
3	2	5	2	x
3	2	5	3	x
3	2	5	4	x
3	2	5	5	x
3	3	0	0	x
3	3	0	1	x
3	3	0	2	x
3	3	0	3	x
3	3	0	4	x
3	3	0	5	x
3	3	1	0	x
3	3	1	1	x
3	3	1	2	x
3	3	1	3	x
3	3	1	4	x
3	3	1	5	x
3	3	2	0	x
3	3	2	1	x
3	3	2	2	x
3	3	2	3	x
3	3	2	4	x
3	3	2	5	x
3	3	3	0	x
3	3	3	1	x
3	3	3	2	x
3	3	3	3	x
3	3	3	4	x
3	3	3	5	x
3	3	4	0	x
3	3	4	1	x
3	3	4	2	x
3	3	4	3	x
3	3	4	4	x
3	3	4	5	x
3	3	5	0	x
3	3	5	1	x
3	3	5	2	x
3	3	5	3	x
3	3	5	4	x
3	3	5	5	x
3	4	0	0	x
3	4	0	1	x
3	4	0	2	x
3	4	0	3	x
3	4	0	4	x
3	4	0	5	x

3	4	1	0	x
3	4	1	1	x
3	4	1	2	x
3	4	1	3	x
3	4	1	4	x
3	4	1	5	x
3	4	2	0	x
3	4	2	1	x
3	4	2	2	x
3	4	2	3	x
3	4	2	4	x
3	4	2	5	x
3	4	3	0	x
3	4	3	1	x
3	4	3	2	x
3	4	3	3	x
3	4	3	4	x
3	4	3	5	x
3	4	4	0	x
3	4	4	1	x
3	4	4	2	x
3	4	4	3	x
3	4	4	4	x
3	4	4	5	x
3	4	5	0	x
3	4	5	1	x
3	4	5	2	x
3	4	5	3	x
3	4	5	4	x
3	4	5	5	x
3	5	0	0	x
3	5	0	1	x
3	5	0	2	x
3	5	0	3	x
3	5	0	4	x
3	5	0	5	x
3	5	1	0	x
3	5	1	1	x
3	5	1	2	x
3	5	1	3	x
3	5	1	4	x
3	5	1	5	x
3	5	2	0	x
3	5	2	1	x
3	5	2	2	x
3	5	2	3	x
3	5	2	4	x
3	5	2	5	x

3	5	3	0	x
3	5	3	1	x
3	5	3	2	x
3	5	3	3	x
3	5	3	4	x
3	5	3	5	x
3	5	4	0	x
3	5	4	1	x
3	5	4	2	x
3	5	4	3	x
3	5	4	4	x
3	5	4	5	x
3	5	5	0	x
3	5	5	1	x
3	5	5	2	x
3	5	5	3	x
3	5	5	4	x
3	5	5	5	x
4	0	0	0	x
4	0	0	1	x
4	0	0	2	x
4	0	0	3	x
4	0	0	4	x
4	0	0	5	x
4	0	1	0	x
4	0	1	1	x
4	0	1	2	x
4	0	1	3	x
4	0	1	4	x
4	0	1	5	x
4	0	2	0	x
4	0	2	1	x
4	0	2	2	x
4	0	2	3	x
4	0	2	4	x
4	0	2	5	x
4	0	3	0	x
4	0	3	1	x
4	0	3	2	x
4	0	3	3	x
4	0	3	4	x
4	0	3	5	x
4	0	4	0	x
4	0	4	1	x
4	0	4	2	x
4	0	4	3	x
4	0	4	4	x
4	0	4	5	x

4	0	5	0	x
4	0	5	1	x
4	0	5	2	x
4	0	5	3	x
4	0	5	4	x
4	0	5	5	x
4	1	0	0	x
4	1	0	1	x
4	1	0	2	x
4	1	0	3	x
4	1	0	4	x
4	1	0	5	x
4	1	1	0	x
4	1	1	1	x
4	1	1	2	x
4	1	1	3	x
4	1	1	4	x
4	1	1	5	x
4	1	2	0	x
4	1	2	1	x
4	1	2	2	x
4	1	2	3	x
4	1	2	4	x
4	1	2	5	x
4	1	3	0	x
4	1	3	1	x
4	1	3	2	x
4	1	3	3	x
4	1	3	4	x
4	1	3	5	x
4	1	4	0	x
4	1	4	1	x
4	1	4	2	x
4	1	4	3	x
4	1	4	4	x
4	1	4	5	x
4	1	5	0	x
4	1	5	1	x
4	1	5	2	x
4	1	5	3	x
4	1	5	4	x
4	1	5	5	x
4	2	0	0	x
4	2	0	1	x
4	2	0	2	x
4	2	0	3	x
4	2	0	4	x
4	2	0	5	x

4	2	1	0	x
4	2	1	1	x
4	2	1	2	x
4	2	1	3	x
4	2	1	4	x
4	2	1	5	x
4	2	2	0	x
4	2	2	1	x
4	2	2	2	x
4	2	2	3	x
4	2	2	4	x
4	2	2	5	x
4	2	3	0	x
4	2	3	1	x
4	2	3	2	x
4	2	3	3	x
4	2	3	4	x
4	2	3	5	x
4	2	4	0	x
4	2	4	1	x
4	2	4	2	x
4	2	4	3	x
4	2	4	4	x
4	2	4	5	x
4	2	5	0	x
4	2	5	1	x
4	2	5	2	x
4	2	5	3	x
4	2	5	4	x
4	2	5	5	x
4	3	0	0	x
4	3	0	1	x
4	3	0	2	x
4	3	0	3	x
4	3	0	4	x
4	3	0	5	x
4	3	1	0	x
4	3	1	1	x
4	3	1	2	x
4	3	1	3	x
4	3	1	4	x
4	3	1	5	x
4	3	2	0	x
4	3	2	1	x
4	3	2	2	x
4	3	2	3	x
4	3	2	4	x
4	3	2	5	x

4	3	3	0	x
4	3	3	1	x
4	3	3	2	x
4	3	3	3	x
4	3	3	4	x
4	3	3	5	x
4	3	4	0	x
4	3	4	1	x
4	3	4	2	x
4	3	4	3	x
4	3	4	4	x
4	3	4	5	x
4	3	5	0	x
4	3	5	1	x
4	3	5	2	x
4	3	5	3	x
4	3	5	4	x
4	3	5	5	x
4	4	0	0	x
4	4	0	1	x
4	4	0	2	x
4	4	0	3	x
4	4	0	4	x
4	4	0	5	x
4	4	1	0	x
4	4	1	1	x
4	4	1	2	x
4	4	1	3	x
4	4	1	4	x
4	4	1	5	x
4	4	2	0	x
4	4	2	1	x
4	4	2	2	x
4	4	2	3	x
4	4	2	4	x
4	4	2	5	x
4	4	3	0	x
4	4	3	1	x
4	4	3	2	x
4	4	3	3	x
4	4	3	4	x
4	4	3	5	x
4	4	4	0	x
4	4	4	1	x
4	4	4	2	x
4	4	4	3	x
4	4	4	4	x
4	4	4	5	x

4	4	5	0	x
4	4	5	1	x
4	4	5	2	x
4	4	5	3	x
4	4	5	4	x
4	4	5	5	x
4	5	0	0	x
4	5	0	1	x
4	5	0	2	x
4	5	0	3	x
4	5	0	4	x
4	5	0	5	x
4	5	1	0	x
4	5	1	1	x
4	5	1	2	x
4	5	1	3	x
4	5	1	4	x
4	5	1	5	x
4	5	2	0	x
4	5	2	1	x
4	5	2	2	x
4	5	2	3	x
4	5	2	4	x
4	5	2	5	x
4	5	3	0	x
4	5	3	1	x
4	5	3	2	x
4	5	3	3	x
4	5	3	4	x
4	5	3	5	x
4	5	4	0	x
4	5	4	1	x
4	5	4	2	x
4	5	4	3	x
4	5	4	4	x
4	5	4	5	x
4	5	5	0	x
4	5	5	1	x
4	5	5	2	x
4	5	5	3	x
4	5	5	4	x
4	5	5	5	x
5	0	0	0	x
5	0	0	1	x
5	0	0	2	x
5	0	0	3	x
5	0	0	4	x
5	0	0	5	x

5	0	1	0	x
5	0	1	1	x
5	0	1	2	x
5	0	1	3	x
5	0	1	4	x
5	0	1	5	x
5	0	2	0	x
5	0	2	1	x
5	0	2	2	x
5	0	2	3	x
5	0	2	4	x
5	0	2	5	x
5	0	3	0	x
5	0	3	1	x
5	0	3	2	x
5	0	3	3	x
5	0	3	4	x
5	0	3	5	x
5	0	4	0	x
5	0	4	1	x
5	0	4	2	x
5	0	4	3	x
5	0	4	4	x
5	0	4	5	x
5	0	5	0	x
5	0	5	1	x
5	0	5	2	x
5	0	5	3	x
5	0	5	4	x
5	0	5	5	x
5	1	0	0	x
5	1	0	1	x
5	1	0	2	x
5	1	0	3	x
5	1	0	4	x
5	1	0	5	x
5	1	1	0	x
5	1	1	1	x
5	1	1	2	x
5	1	1	3	x
5	1	1	4	x
5	1	1	5	x
5	1	2	0	x
5	1	2	1	x
5	1	2	2	x
5	1	2	3	x
5	1	2	4	x
5	1	2	5	x

5	1	3	0	x
5	1	3	1	x
5	1	3	2	x
5	1	3	3	x
5	1	3	4	x
5	1	3	5	x
5	1	4	0	x
5	1	4	1	x
5	1	4	2	x
5	1	4	3	x
5	1	4	4	x
5	1	4	5	x
5	1	5	0	x
5	1	5	1	x
5	1	5	2	x
5	1	5	3	x
5	1	5	4	x
5	1	5	5	x
5	2	0	0	x
5	2	0	1	x
5	2	0	2	x
5	2	0	3	x
5	2	0	4	x
5	2	0	5	x
5	2	1	0	x
5	2	1	1	x
5	2	1	2	x
5	2	1	3	x
5	2	1	4	x
5	2	1	5	x
5	2	2	0	x
5	2	2	1	x
5	2	2	2	x
5	2	2	3	x
5	2	2	4	x
5	2	2	5	x
5	2	3	0	x
5	2	3	1	x
5	2	3	2	x
5	2	3	3	x
5	2	3	4	x
5	2	3	5	x
5	2	4	0	x
5	2	4	1	x
5	2	4	2	x
5	2	4	3	x
5	2	4	4	x
5	2	4	5	x

5	2	5	0	x
5	2	5	1	x
5	2	5	2	x
5	2	5	3	x
5	2	5	4	x
5	2	5	5	x
5	3	0	0	x
5	3	0	1	x
5	3	0	2	x
5	3	0	3	x
5	3	0	4	x
5	3	0	5	x
5	3	1	0	x
5	3	1	1	x
5	3	1	2	x
5	3	1	3	x
5	3	1	4	x
5	3	1	5	x
5	3	2	0	x
5	3	2	1	x
5	3	2	2	x
5	3	2	3	x
5	3	2	4	x
5	3	2	5	x
5	3	3	0	x
5	3	3	1	x
5	3	3	2	x
5	3	3	3	x
5	3	3	4	x
5	3	3	5	x
5	3	4	0	x
5	3	4	1	x
5	3	4	2	x
5	3	4	3	x
5	3	4	4	x
5	3	4	5	x
5	3	5	0	x
5	3	5	1	x
5	3	5	2	x
5	3	5	3	x
5	3	5	4	x
5	3	5	5	x
5	4	0	0	x
5	4	0	1	x
5	4	0	2	x
5	4	0	3	x
5	4	0	4	x
5	4	0	5	x

5	4	1	0	x
5	4	1	1	x
5	4	1	2	x
5	4	1	3	x
5	4	1	4	x
5	4	1	5	x
5	4	2	0	x
5	4	2	1	x
5	4	2	2	x
5	4	2	3	x
5	4	2	4	x
5	4	2	5	x
5	4	3	0	x
5	4	3	1	x
5	4	3	2	x
5	4	3	3	x
5	4	3	4	x
5	4	3	5	x
5	4	4	0	x
5	4	4	1	x
5	4	4	2	x
5	4	4	3	x
5	4	4	4	x
5	4	4	5	x
5	4	5	0	x
5	4	5	1	x
5	4	5	2	x
5	4	5	3	x
5	4	5	4	x
5	4	5	5	x
5	5	0	0	x
5	5	0	1	x
5	5	0	2	x
5	5	0	3	x
5	5	0	4	x
5	5	0	5	x
5	5	1	0	x
5	5	1	1	x
5	5	1	2	x
5	5	1	3	x
5	5	1	4	x
5	5	1	5	x
5	5	2	0	x
5	5	2	1	x
5	5	2	2	x
5	5	2	3	x
5	5	2	4	x
5	5	2	5	x

5	5	3	0	x
5	5	3	1	x
5	5	3	2	x
5	5	3	3	x
5	5	3	4	x
5	5	3	5	x
5	5	4	0	x
5	5	4	1	x
5	5	4	2	x
5	5	4	3	x
5	5	4	4	x
5	5	4	5	x
5	5	5	0	x
5	5	5	1	x
5	5	5	2	x
5	5	5	3	x
5	5	5	4	x
5	5	5	5	x

Table SI13. Hsf1 node.

Table entries: 216; references: 15, 16, 17.

RepX	Ssa1	Ssa2	<<Hsf1>> (true)
0	0	0	5
0	0	1	4
0	0	2	3
0	0	3	2
0	0	4	1
0	0	5	x
0	1	0	4
0	1	1	3
0	1	2	2
0	1	3	1
0	1	4	0
0	1	5	x
0	2	0	x
0	2	1	x
0	2	2	x
0	2	3	x
0	2	4	x
0	2	5	x
0	3	0	x
0	3	1	x
0	3	2	x
0	3	3	x
0	3	4	x
0	3	5	x
0	4	0	x

Complex regulation of Hsf1-Skn7 activities by the catalytic subunits of PKA in Saccharomyces cerevisiae: experimental and computational approaches.

0	4	1	x
0	4	2	x
0	4	3	x
0	4	4	x
0	4	5	x
0	5	0	x
0	5	1	x
0	5	2	x
0	5	3	x
0	5	4	x
0	5	5	x
1	0	0	4
1	0	1	3
1	0	2	2
1	0	3	1
1	0	4	0
1	0	5	x
1	1	0	3
1	1	1	2
1	1	2	1
1	1	3	0
1	1	4	0
1	1	5	x
1	2	0	x
1	2	1	x
1	2	2	x
1	2	3	x
1	2	4	x
1	2	5	x
1	3	0	x
1	3	1	x
1	3	2	x
1	3	3	x
1	3	4	x
1	3	5	x
1	4	0	x
1	4	1	x
1	4	2	x
1	4	3	x
1	4	4	x
1	4	5	x
1	5	0	x
1	5	1	x
1	5	2	x
1	5	3	x
1	5	4	x
1	5	5	x
2	0	0	2

2	0	1	1
2	0	2	0
2	0	3	0
2	0	4	0
2	0	5	x
2	1	0	1
2	1	1	0
2	1	2	0
2	1	3	0
2	1	4	0
2	1	5	x
2	2	0	x
2	2	1	x
2	2	2	x
2	2	3	x
2	2	4	x
2	2	5	x
2	3	0	x
2	3	1	x
2	3	2	x
2	3	3	x
2	3	4	x
2	3	5	x
2	4	0	x
2	4	1	x
2	4	2	x
2	4	3	x
2	4	4	x
2	4	5	x
2	5	0	x
2	5	1	x
2	5	2	x
2	5	3	x
2	5	4	x
2	5	5	x
3	0	0	x
3	0	1	x
3	0	2	x
3	0	3	x
3	0	4	x
3	0	5	x
3	1	0	x
3	1	1	x
3	1	2	x
3	1	3	x
3	1	4	x
3	1	5	x
3	2	0	x

3	2	1	x
3	2	2	x
3	2	3	x
3	2	4	x
3	2	5	x
3	3	0	x
3	3	1	x
3	3	2	x
3	3	3	x
3	3	4	x
3	3	5	x
3	4	0	x
3	4	1	x
3	4	2	x
3	4	3	x
3	4	4	x
3	4	5	x
3	5	0	x
3	5	1	x
3	5	2	x
3	5	3	x
3	5	4	x
3	5	5	x
4	0	0	x
4	0	1	x
4	0	2	x
4	0	3	x
4	0	4	x
4	0	5	x
4	1	0	x
4	1	1	x
4	1	2	x
4	1	3	x
4	1	4	x
4	1	5	x
4	2	0	x
4	2	1	x
4	2	2	x
4	2	3	x
4	2	4	x
4	2	5	x
4	3	0	x
4	3	1	x
4	3	2	x
4	3	3	x
4	3	4	x
4	3	5	x
4	4	0	x

4	4	1	x
4	4	2	x
4	4	3	x
4	4	4	x
4	4	5	x
4	5	0	x
4	5	1	x
4	5	2	x
4	5	3	x
4	5	4	x
4	5	5	x
5	0	0	x
5	0	1	x
5	0	2	x
5	0	3	x
5	0	4	x
5	0	5	x
5	1	0	x
5	1	1	x
5	1	2	x
5	1	3	x
5	1	4	x
5	1	5	x
5	2	0	x
5	2	1	x
5	2	2	x
5	2	3	x
5	2	4	x
5	2	5	x
5	3	0	x
5	3	1	x
5	3	2	x
5	3	3	x
5	3	4	x
5	3	5	x
5	4	0	x
5	4	1	x
5	4	2	x
5	4	3	x
5	4	4	x
5	4	5	x
5	5	0	x
5	5	1	x
5	5	2	x
5	5	3	x
5	5	4	x
5	5	5	x

.

Table SI14. *HSE-lacZ* node.

Table entries: 6; references: 5, 17, 18, 19, 23.

**Hsf1**	**<<*HSE-lacZ*>> (true)**
0	0
1	1
2	2
3	3
4	4
5	11

Table SI15. Heat Shock node.

Table entries: 6; references: 2, 3.

**Heat**	**<<Heat>> (false)**
0	0
1	0
2	0
3	0
4	0
5	0

References supporting Sections 3 and 4:

1. Geymonat M *et al.*: **Ssa1p chaperone interacts with the guanine nucleotide exchange factor of ras Cdc25p and controls the cAMP pathway in *Saccharomyces cerevisiae*.** *Mol Microbiol* 1998, **30**:855-864.
2. Fig. 1, this work.
3. Section 2, Supplementary Information, this work.
4. Section 3, Supplementary Information, this work.
5. Fig. 7, this work.
6. Field J *et al.*: **Guanine nucleotide activation of, and competition between, RAS proteins from *Saccharomyces cerevisiae*.** Mol Cell Biol 1987, **7**:2128-2133.
7. Broek D **et al.**: **The *S. cerevisiae CDC25* gene product regulates the RAS/adenylate cyclase pathway.** *Cell* 1987, **48**: 789-799.
8. Tatchell K: ***RAS* genes and growth control in *Saccharomyces cerevisiae*.** *J Bacteriol* 1986, **166**:364-367.
9. Toda T *et al.*: **In yeast, RAS proteins are controlling elements of adenylate cyclase.** *Cell* 1985, **40**:27-36.
10. Thevelein JM, De Winde JH: **Novel sensing mechanisms and targets for the cAMP-protein kinase A pathway in the yeast *Saccharomyces cerevisiae*.** *Mol Microbiol* 1999, **33**:904-918.
11. Kataoka T, *et al.*: **DNA sequence and characterization of the *S. cerevisiae* gene encoding adenylate cyclase.** *Cell* 1985, **43**:493-505.
12. Wigler M: **Cloning and characterization of *BCY1*, a locus encoding a regulatory subunit of the cyclic AMP-dependent protein kinase in *Saccharomyces cerevisiae*.** *Mol Cell Biol* 1987 **7**:1371-1377.
13. Toda T *et al.*: **Three different genes in *S. cerevisiae* encode the catalytic subunits of the cAMP-dependent protein kinase.** *Cell* 1987, **50**:277-287.
14. Fig. 5, this work.
15. Fig. 6, this work.
16. Craig EA, Gross CA: **Is hsp70 the cellular thermometer?** *Trends Biochem Sci* 1991, **16**:135-140.
17. Wu C: **Heat shock transcription factors: structure and regulation.** *Annu Rev Cell Dev Biol* 1995, **11**:441–469.
18. Fernandes M *et al.*: **Fine structure analyses of the *Drosophila* and *Saccharomyces* heat shock factor-heat shock element interactions.** Nucleic Acids Res 1994, **22**:167-173.
19. Raitt DC *et al.*: **The Skn7 response regulator of *Saccharomyces cerevisiae* interacts with Hsf1 in vivo and is required for the induction of heat shock genes by oxidative stress.** *Mol Biol Cell* 2000, **11**:2335-2347.
20. Bonner JJ *et al.*: **Complex regulation of the yeast heat shock transcription**

factor. *Mol Biol Cell* 2000, **11**:1739-1751.

21. Hahn JS *et al*: **Genome-wide analysis of the biology of stress responses through heat shock transcription factor.** *Mol Cell Biol* 2004, **24**:5249-5256.
22. Mollapour M *et al*.: **Swe1^{Wee1}-dependent tyrosine phosphorylation of Hsp90 regulates distinct facets of chaperone function.** *Molecular Cell* 2010, **37**:333-343.
23. Figs. 2, 3, this work.
24. Figs. 4, and 8, this work.
