# Supplementary material for: Complex regulation of Hsf1-Skn7 activities by the catalytic subunits of PKA in Saccharomyces cerevisiae: experimental and computational evidences
Source: BMC Syst Biol. 2015 Jul 27;9:42. doi: 10.1186/s12918-015-0185-8 (PMC4515323; doi:10.1186/s12918-015-0185-8)
Supplement: Additional file 4: — Text S1. The attractors for several simulated strains are presented here. [file 12918_2015_185_MOESM4_ESM.pdf]

Complex regulation of Hsf1-Skn7 activities by the catalytic subunits of PKA in Saccharomyces cerevisiae: experimental and computational approaches.

Text S1:

The attractors for several simulated strains are presented here. Each row corresponds to a time step in the simulation, and the states are ordered by succession. This means than when multiple states are present in an attractor, the first state will be repeated after the last one over and over again. The average expression (over the attractor) for each node is shown below the dotted line. Basin sizes (sampled) and weights used for the WDM are shown at the end of the list of all the attractors for each network.

Network: WT

Total Attractors: 14

Attractor: 0

Cdc25	Ras2	cAMP	Bcy1	Tpk1	Tpk2	Tpk3	Tpk1*	Tpk3*	RepX	Ssa1	Ssa2	Hsf1	*HSE-lacZ*	Heat
5	4	4	1	2	4	2	0	0	0	1	3	0	1	0
4	5	4	1	2	4	2	0	0	0	1	3	1	0	0
4	4	5	1	2	4	2	0	0	0	1	3	1	1	0
4	4	4	0	2	4	2	0	0	0	1	3	1	1	0
4	4	4	1	3	5	3	0	0	0	1	3	1	1	0
4	4	4	1	2	4	2	0	0	0	1	4	1	1	0

4.17	4.17	4.17	0.83	2.17	4.17	2.17	0.0	0.0	0.0	1.0	3.17	0.83	0.83	0.0

Attractor: 1

Cdc25	Ras2	cAMP	Bcy1	Tpk1	Tpk2	Tpk3	Tpk1*	Tpk3*	RepX	Ssa1	Ssa2	Hsf1	*HSE-lacZ*	Heat
5	5	4	0	2	4	2	0	0	0	1	3	0	0	0
4	5	5	1	3	5	3	0	0	0	1	3	1	0	0
4	4	5	0	2	4	2	0	0	0	1	4	1	1	0
5	4	4	0	3	5	3	0	0	0	1	3	0	1	0
4	5	4	1	3	5	3	0	0	0	1	4	1	0	0

5	4	5	1	2	4	2	0	0	0	1	4	0	1	0

4.5	4.5	4.5	0.5	2.5	4.5	2.5	0.0	0.0	0.0	1.0	3.5	0.5	0.5	0.0

Attractor: 2

Cdc25	Ras2	cAMP	Bcy1	Tpk1	Tpk2	Tpk3	Tpk1*	Tpk3*	RepX	Ssa1	Ssa2	Hsf1	*HSE-lacZ*	Heat
5	5	4	1	2	4	2	0	0	0	1	3	0	0	0
4	5	5	1	2	4	2	0	0	0	1	3	1	0	0
4	4	5	0	2	4	2	0	0	0	1	3	1	1	0
4	4	4	0	3	5	3	0	0	0	1	3	1	1	0
4	4	4	1	3	5	3	0	0	0	1	4	1	1	0
5	4	4	1	2	4	2	0	0	0	1	4	0	1	0

4.33	4.33	4.33	0.67	2.33	4.33	2.33	0.0	0.0	0.0	1.0	3.33	0.67	0.67	0.0

Attractor: 3

Cdc25	Ras2	cAMP	Bcy1	Tpk1	Tpk2	Tpk3	Tpk1*	Tpk3*	RepX	Ssa1	Ssa2	Hsf1	*HSE-lacZ*	Heat
4	4	4	1	2	4	2	0	0	0	1	3	1	1	0

4.0	4.0	4.0	1.0	2.0	4.0	2.0	0.0	0.0	0.0	1.0	3.0	1.0	1.0	0.0

Attractor: 4

Cdc25	Ras2	cAMP	Bcy1	Tpk1	Tpk2	Tpk3	Tpk1*	Tpk3*	RepX	Ssa1	Ssa2	Hsf1	*HSE-lacZ*	Heat
5	4	5	1	2	4	2	0	0	0	1	3	0	1	0
4	5	4	0	2	4	2	0	0	0	1	3	1	0	0

4	4	5	1	3	5	3	0	0	0	1	3	1	1	0
4	4	4	0	2	4	2	0	0	0	1	4	1	1	0
5	4	4	1	3	5	3	0	0	0	1	3	0	1	0
4	5	4	1	2	4	2	0	0	0	1	4	1	0	0
--														
4.33	4.33	4.33	0.67	2.33	4.33	2.33	0.0	0.0	0.0	1.0	3.33	0.67	0.67	0.0

Attractor: 5

Cdc25	Ras2	cAMP	Bcy1	Tpk1	Tpk2	Tpk3	Tpk1*	Tpk3*	RepX	Ssa1	Ssa2	Hsf1	*HSE-lacZ*	Heat
5	5	5	0	2	4	2	0	0	0	1	4	0	0	0
5	5	5	0	3	5	3	0	0	0	1	3	0	0	0
4	5	5	0	3	5	3	0	0	0	1	4	1	0	0
5	4	5	0	3	5	3	0	0	0	1	4	0	1	0
5	5	4	0	3	5	3	0	0	0	1	4	0	0	0
5	5	5	1	3	5	3	0	0	0	1	4	0	0	0
--														
4.83	4.83	4.83	0.17	2.83	4.83	2.83	0.0	0.0	0.0	1.0	3.83	0.17	0.17	0.0

Attractor: 6

Cdc25	Ras2	cAMP	Bcy1	Tpk1	Tpk2	Tpk3	Tpk1*	Tpk3*	RepX	Ssa1	Ssa2	Hsf1	*HSE-lacZ*	Heat
4	5	4	0	2	4	2	0	0	0	1	4	1	0	0
5	4	5	1	3	5	3	0	0	0	1	3	0	1	0
--														
4.5	4.5	4.5	0.5	2.5	4.5	2.5	0.0	0.0	0.0	1.0	3.5	0.5	0.5	0.0

Complex regulation of Hsf1-Skn7 activities by the catalytic subunits of PKA in Saccharomyces cerevisiae: experimental and computational approaches.

Attractor: 7

Cdc25	Ras2	cAMP	Bcy1	Tpk1	Tpk2	Tpk3	Tpk1*	Tpk3*	RepX	Ssa1	Ssa2	Hsf1	*HSE-lacZ*	Heat
5	4	4	0	2	4	2	0	0	0	1	3	0	1	0
4	5	4	1	3	5	3	0	0	0	1	3	1	0	0
4	4	5	1	2	4	2	0	0	0	1	4	1	1	0
--														
4.33	4.33	4.33	0.67	2.33	4.33	2.33	0.0	0.0	0.0	1.0	3.33	0.67	0.67	0.0

Attractor: 8

Cdc25	Ras2	cAMP	Bcy1	Tpk1	Tpk2	Tpk3	Tpk1*	Tpk3*	RepX	Ssa1	Ssa2	Hsf1	*HSE-lacZ*	Heat
5	5	5	1	2	4	2	0	0	0	1	3	0	0	0
4	5	5	0	2	4	2	0	0	0	1	3	1	0	0
4	4	5	0	3	5	3	0	0	0	1	3	1	1	0
4	4	4	0	3	5	3	0	0	0	1	4	1	1	0
5	4	4	1	3	5	3	0	0	0	1	4	0	1	0
5	5	4	1	2	4	2	0	0	0	1	4	0	0	0
--														
4.5	4.5	4.5	0.5	2.5	4.5	2.5	0.0	0.0	0.0	1.0	3.5	0.5	0.5	0.0

Attractor: 9

Cdc25	Ras2	cAMP	Bcy1	Tpk1	Tpk2	Tpk3	Tpk1*	Tpk3*	RepX	Ssa1	Ssa2	Hsf1	*HSE-lacZ*	Heat
5	5	4	0	2	4	2	0	0	0	1	4	0	0	0
5	5	5	1	3	5	3	0	0	0	1	3	0	0	0
4	5	5	0	2	4	2	0	0	0	1	4	1	0	0
5	4	5	0	3	5	3	0	0	0	1	3	0	1	0

4	5	4	0	3	5	3	0	0	0	1	4	1	0	0
5	4	5	1	3	5	3	0	0	0	1	4	0	1	0

4.67	4.67	4.67	0.33	2.67	4.67	2.67	0.0	0.0	0.0	1.0	3.67	0.33	0.33	0.0

Attractor: 10

Cdc25	Ras2	cAMP	Bcy1	Tpk1	Tpk2	Tpk3	Tpk1*	Tpk3*	RepX	Ssa1	Ssa2	Hsf1	*HSE-lacZ*	Heat
5	4	5	0	2	4	2	0	0	0	1	3	0	1	0
4	5	4	0	3	5	3	0	0	0	1	3	1	0	0
4	4	5	1	3	5	3	0	0	0	1	4	1	1	0
5	4	4	0	2	4	2	0	0	0	1	4	0	1	0
5	5	4	1	3	5	3	0	0	0	1	3	0	0	0
4	5	5	1	2	4	2	0	0	0	1	4	1	0	0

4.5	4.5	4.5	0.5	2.5	4.5	2.5	0.0	0.0	0.0	1.0	3.5	0.5	0.5	0.0

Attractor: 11

Cdc25	Ras2	cAMP	Bcy1	Tpk1	Tpk2	Tpk3	Tpk1*	Tpk3*	RepX	Ssa1	Ssa2	Hsf1	*HSE-lacZ*	Heat
5	5	5	0	2	4	2	0	0	0	1	3	0	0	0
4	5	5	0	3	5	3	0	0	0	1	3	1	0	0
4	4	5	0	3	5	3	0	0	0	1	4	1	1	0
5	4	4	0	3	5	3	0	0	0	1	4	0	1	0
5	5	4	1	3	5	3	0	0	0	1	4	0	0	0
5	5	5	1	2	4	2	0	0	0	1	4	0	0	0

4.67	4.67	4.67	0.33	2.67	4.67	2.67	0.0	0.0	0.0	1.0	3.67	0.33	0.33	0.0

Attractor: 12

Cdc25	Ras2	cAMP	Bcy1	Tpk1	Tpk2	Tpk3	Tpk1*	Tpk3*	RepX	Ssa1	Ssa2	Hsf1	*HSE-lacZ*	Heat
5	4	5	0	2	4	2	0	0	0	1	4	0	1	0
5	5	4	0	3	5	3	0	0	0	1	3	0	0	0
4	5	5	1	3	5	3	0	0	0	1	4	1	0	0

4.67	4.67	4.67	0.33	2.67	4.67	2.67	0.0	0.0	0.0	1.0	3.67	0.33	0.33	0.0

Attractor: 13

Cdc25	Ras2	cAMP	Bcy1	Tpk1	Tpk2	Tpk3	Tpk1*	Tpk3*	RepX	Ssa1	Ssa2	Hsf1	*HSE-lacZ*	Heat
5	5	5	0	3	5	3	0	0	0	1	4	0	0	0

5.0	5.0	5.0	0.0	3.0	5.0	3.0	0.0	0.0	0.0	1.0	4.0	0.0	0.0	0.0

################ Basins of Attraction ##################################

A:0 -> Basin: 650156 Percentage: 38.94959574029609% Weight: 0.3894959574029609 TotalConditions: 1669224.0

A:1 -> Basin: 52271 Percentage: 3.131455095301769% Weight: 0.03131455095301769 TotalConditions: 1669224.0

A:2 -> Basin: 203515 Percentage: 12.192192300134673% Weight: 0.12192192300134673 TotalConditions: 1669224.0

A:3 -> Basin: 324488 Percentage: 19.439452104690563% Weight: 0.19439452104690563 TotalConditions: 1669224.0

A:4 -> Basin: 185656 Percentage: 11.122293952159806% Weight: 0.11122293952159806 TotalConditions: 1669224.0

A:5 -> Basin: 3602 Percentage: 0.21578889352178018% Weight: 0.002157888935217802 TotalConditions: 1669224.0

A:6 -> Basin: 16091 Percentage: 0.9639808677565144% Weight: 0.009639808677565144 TotalConditions: 1669224.0

A:7 -> Basin: 90533 Percentage: 5.423657939258002% Weight: 0.054236579392580024 TotalConditions: 1669224.0

A:8 -> Basin: 56086 Percentage: 3.3600044092344707% Weight: 0.033600044092344704 TotalConditions: 1669224.0

A:9 -> Basin: 13583 Percentage: 0.8137314105236925% Weight: 0.008137314105236925 TotalConditions: 1669224.0

A:10 -> Basin: 51953 Percentage: 3.1124043268009567% Weight: 0.031124043268009566 TotalConditions: 1669224.0

A:11 -> Basin: 14294 Percentage: 0.8563260533038106% Weight: 0.008563260533038106 TotalConditions: 1669224.0

A:12 -> Basin: 6825 Percentage: 0.4088726258429066% Weight: 0.004088726258429066 TotalConditions: 1669224.0

A:13 -> Basin: 171 Percentage: 0.010244281174965134% Weight: 1.0244281174965134E-4 TotalConditions: 1669224.0

Network: *bcy1Δ*

Total Attractors: 1

Cdc25	Ras2	cAMP	Bcy1	Tpk1	Tpk2	Tpk3	Tpk1*	Tpk3*	RepX	Ssa1	Ssa2	Hsf1	*HSE-lacZ*	Heat
5	5	5	0	3	5	3	0	0	0	1	4	0	0	0

5.0	5.0	5.0	0.0	3.0	5.0	3.0	0.0	0.0	0.0	1.0	4.0	0.0	0.0	0.0

################ Basins of Attraction ##################################

A:0 -> Basin: 1230517 Percentage: 100.0% Weight: 1.0 TotalConditions: 1230517.0

Network: *ssa1Δ ssa2Δ*

Attractors: 1

Cdc25	Ras2	cAMP	Bcy1	Tpk1	Tpk2	Tpk3	Tpk1*	Tpk3*	RepX	Ssa1	Ssa2	Hsf1	*HSE-lacZ*	Heat
1	1	1	4	0	2	0	0	0	0	0	0	5	11	0

1.0	1.0	1.0	4.0	0.0	2.0	0.0	0.0	0.0	0.0	0.0	0.0	5.0	11.0	0.0

################ Basins of Attraction ##################################

A:0 -> Basin: 1278495 Percentage: 100.0% Weight: 1.0 TotalConditions: 1278495.0

Network: *tpk1Δ tpk3Δ*

Total Attractors: 14

Attractor: 0

Cdc25	Ras2	cAMP	Bcy1	Tpk1	Tpk2	Tpk3	Tpk1*	Tpk3*	RepX	Ssa1	Ssa2	Hsf1	*HSE-lacZ*	Heat
5	4	4	1	0	4	0	0	0	0	1	3	0	1	0
4	5	4	1	0	4	0	0	0	0	1	3	1	0	0
4	4	5	1	0	4	0	0	0	0	1	3	1	1	0
4	4	4	0	0	4	0	0	0	0	1	3	1	1	0
4	4	4	1	0	5	0	0	0	0	1	3	1	1	0
4	4	4	1	0	4	0	0	0	0	1	4	1	1	0

4.17	4.17	4.17	0.83	0.0	4.17	0.0	0.0	0.0	0.0	1.0	3.17	0.83	0.83	0.0

Attractor: 1

Cdc25	Ras2	cAMP	Bcy1	Tpk1	Tpk2	Tpk3	Tpk1*	Tpk3*	RepX	Ssa1	Ssa2	Hsf1	*HSE-lacZ*	Heat
4	5	4	0	0	4	0	0	0	0	1	4	1	0	0
5	4	5	1	0	5	0	0	0	0	1	3	0	1	0

4.5	4.5	4.5	0.5	0.0	4.5	0.0	0.0	0.0	0.0	1.0	3.5	0.5	0.5	0.0

Complex regulation of Hsf1-Skn7 activities by the catalytic subunits of PKA in Saccharomyces cerevisiae: experimental and computational approaches.

Attractor: 2

Cdc25	Ras2	cAMP	Bcy1	Tpk1	Tpk2	Tpk3	Tpk1*	Tpk3*	RepX	Ssa1	Ssa2	Hsf1	*HSE-lacZ*	Heat
4	4	4	0	0	4	0	0	0	0	1	4	1	1	0
5	4	4	1	0	5	0	0	0	0	1	3	0	1	0
4	5	4	1	0	4	0	0	0	0	1	4	1	0	0
5	4	5	1	0	4	0	0	0	0	1	3	0	1	0
4	5	4	0	0	4	0	0	0	0	1	3	1	0	0
4	4	5	1	0	5	0	0	0	0	1	3	1	1	0
--														
4.33	4.33	4.33	0.67	0.0	4.33	0.0	0.0	0.0	0.0	1.0	3.33	0.67	0.67	0.0

Attractor: 3

Cdc25	Ras2	cAMP	Bcy1	Tpk1	Tpk2	Tpk3	Tpk1*	Tpk3*	RepX	Ssa1	Ssa2	Hsf1	*HSE-lacZ*	Heat
4	4	5	0	0	4	0	0	0	0	1	3	1	1	0
4	4	4	0	0	5	0	0	0	0	1	3	1	1	0
4	4	4	1	0	5	0	0	0	0	1	4	1	1	0
5	4	4	1	0	4	0	0	0	0	1	4	0	1	0
5	5	4	1	0	4	0	0	0	0	1	3	0	0	0
4	5	5	1	0	4	0	0	0	0	1	3	1	0	0
--														
4.33	4.33	4.33	0.67	0.0	4.33	0.0	0.0	0.0	0.0	1.0	3.33	0.67	0.67	0.0

Attractor: 4

Cdc25	Ras2	cAMP	Bcy1	Tpk1	Tpk2	Tpk3	Tpk1*	Tpk3*	RepX	Ssa1	Ssa2	Hsf1	*HSE-lacZ*	Heat
5	4	5	0	0	4	0	0	0	0	1	3	0	1	0

4	5	4	0	0	5	0	0	0	0	1	3	1	0	0
4	4	5	1	0	5	0	0	0	0	1	4	1	1	0
5	4	4	0	0	4	0	0	0	0	1	4	0	1	0
5	5	4	1	0	5	0	0	0	0	1	3	0	0	0
4	5	5	1	0	4	0	0	0	0	1	4	1	0	0

4.5	4.5	4.5	0.5	0.0	4.5	0.0	0.0	0.0	0.0	1.0	3.5	0.5	0.5	0.0

Attractor: 5

Cdc25	Ras2	cAMP	Bcy1	Tpk1	Tpk2	Tpk3	Tpk1*	Tpk3*	RepX	Ssa1	Ssa2	Hsf1	*HSE*-*lacZ*	Heat
4	4	4	1	0	4	0	0	0	0	1	3	1	1	0

4.0	4.0	4.0	1.0	0.0	4.0	0.0	0.0	0.0	0.0	1.0	3.0	1.0	1.0	0.0

Attractor: 6

Cdc25	Ras2	cAMP	Bcy1	Tpk1	Tpk2	Tpk3	Tpk1*	Tpk3*	RepX	Ssa1	Ssa2	Hsf1	*HSE*-*lacZ*	Heat
4	4	5	1	0	4	0	0	0	0	1	4	1	1	0
5	4	4	0	0	4	0	0	0	0	1	3	0	1	0
4	5	4	1	0	5	0	0	0	0	1	3	1	0	0

4.33	4.33	4.33	0.67	0.0	4.33	0.0	0.0	0.0	0.0	1.0	3.33	0.67	0.67	0.0

Attractor: 7

Cdc25	Ras2	cAMP	Bcy1	Tpk1	Tpk2	Tpk3	Tpk1*	Tpk3*	RepX	Ssa1	Ssa2	Hsf1	*HSE*-*lacZ*	Heat
4	5	5	0	0	4	0	0	0	0	1	3	1	0	0

4	4	5	0	0	5	0	0	0	0	1	3	1	1	0
4	4	4	0	0	5	0	0	0	0	1	4	1	1	0
5	4	4	1	0	5	0	0	0	0	1	4	0	1	0
5	5	4	1	0	4	0	0	0	0	1	4	0	0	0
5	5	5	1	0	4	0	0	0	0	1	3	0	0	0

4.5	4.5	4.5	0.5	0.0	4.5	0.0	0.0	0.0	0.0	1.0	3.5	0.5	0.5	0.0

Attractor: 8

Cdc25	Ras2	cAMP	Bcy1	Tpk1	Tpk2	Tpk3	Tpk1*	Tpk3*	RepX	Ssa1	Ssa2	Hsf1	*HSE-lacZ*	Heat
5	4	5	1	0	4	0	0	0	0	1	4	0	1	0
5	5	4	0	0	4	0	0	0	0	1	3	0	0	0
4	5	5	1	0	5	0	0	0	0	1	3	1	0	0
4	4	5	0	0	4	0	0	0	0	1	4	1	1	0
5	4	4	0	0	5	0	0	0	0	1	3	0	1	0
4	5	4	1	0	5	0	0	0	0	1	4	1	0	0

4.5	4.5	4.5	0.5	0.0	4.5	0.0	0.0	0.0	0.0	1.0	3.5	0.5	0.5	0.0

Attractor: 9

Cdc25	Ras2	cAMP	Bcy1	Tpk1	Tpk2	Tpk3	Tpk1*	Tpk3*	RepX	Ssa1	Ssa2	Hsf1	*HSE-lacZ*	Heat
5	5	4	0	0	4	0	0	0	0	1	4	0	0	0
5	5	5	1	0	5	0	0	0	0	1	3	0	0	0
4	5	5	0	0	4	0	0	0	0	1	4	1	0	0
5	4	5	0	0	5	0	0	0	0	1	3	0	1	0

4	5	4	0	0	5	0	0	0	0	1	4	1	0	0
5	4	5	1	0	5	0	0	0	0	1	4	0	1	0

4.67	4.67	4.67	0.33	0.0	4.67	0.0	0.0	0.0	0.0	1.0	3.67	0.33	0.33	0.0

Attractor: 10

Cdc25	Ras2	cAMP	Bcy1	Tpk1	Tpk2	Tpk3	Tpk1*	Tpk3*	RepX	Ssa1	Ssa2	Hsf1	*HSE-lacZ*	Heat
5	5	5	0	0	4	0	0	0	0	1	4	0	0	0
5	5	5	0	0	5	0	0	0	0	1	3	0	0	0
4	5	5	0	0	5	0	0	0	0	1	4	1	0	0
5	4	5	0	0	5	0	0	0	0	1	4	0	1	0
5	5	4	0	0	5	0	0	0	0	1	4	0	0	0
5	5	5	1	0	5	0	0	0	0	1	4	0	0	0

4.83	4.83	4.83	0.17	0.0	4.83	0.0	0.0	0.0	0.0	1.0	3.83	0.17	0.17	0.0

Attractor: 11

Cdc25	Ras2	cAMP	Bcy1	Tpk1	Tpk2	Tpk3	Tpk1*	Tpk3*	RepX	Ssa1	Ssa2	Hsf1	*HSE-lacZ*	Heat
5	5	5	1	0	4	0	0	0	0	1	4	0	0	0
5	5	5	0	0	4	0	0	0	0	1	3	0	0	0
4	5	5	0	0	5	0	0	0	0	1	3	1	0	0
4	4	5	0	0	5	0	0	0	0	1	4	1	1	0
5	4	4	0	0	5	0	0	0	0	1	4	0	1	0
5	5	4	1	0	5	0	0	0	0	1	4	0	0	0

4.67	4.67	4.67	0.33	0.0	4.67	0.0	0.0	0.0	0.0	1.0	3.67	0.33	0.33	0.0

Attractor: 12

Cdc25	Ras2	cAMP	Bcy1	Tpk1	Tpk2	Tpk3	Tpk1*	Tpk3*	RepX	Ssa1	Ssa2	Hsf1	*HSE*-*lacZ*	Heat
5	4	5	0	0	4	0	0	0	0	1	4	0	1	0
5	5	4	0	0	5	0	0	0	0	1	3	0	0	0
4	5	5	1	0	5	0	0	0	0	1	4	1	0	0

4.67	4.67	4.67	0.33	0.0	4.67	0.0	0.0	0.0	0.0	1.0	3.67	0.33	0.33	0.0

Attractor: 13

Cdc25	Ras2	cAMP	Bcy1	Tpk1	Tpk2	Tpk3	Tpk1*	Tpk3*	RepX	Ssa1	Ssa2	Hsf1	*HSE*-*lacZ*	Heat
5	5	5	0	0	5	0	0	0	0	1	4	0	0	0

5.0	5.0	5.0	0.0	0.0	5.0	0.0	0.0	0.0	0.0	1.0	4.0	0.0	0.0	0.0

################ Basins of Attraction #################################

A:0 -> Basin: 605315 Percentage: 39.804186404968135% Weight: 0.39804186404968134 TotalConditions: 1520732.0

A:1 -> Basin: 13726 Percentage: 0.9025916466543743% Weight: 0.009025916466543743 TotalConditions: 1520732.0

A:2 -> Basin: 162063 Percentage: 10.656907331469318% Weight: 0.10656907331469319 TotalConditions: 1520732.0

A:3 -> Basin: 169603 Percentage: 11.152721189532409% Weight: 0.1115272118953241 TotalConditions: 1520732.0

A:4 -> Basin: 42307 Percentage: 2.7820155030603684% Weight: 0.027820155030603682 TotalConditions: 1520732.0

A:5 -> Basin: 333343 Percentage: 21.919904361846793% Weight: 0.21919904361846795 TotalConditions: 1520732.0

A:6 -> Basin: 80798 Percentage: 5.31309921800817% Weight: 0.053130992180081694 TotalConditions: 1520732.0

A:7 -> Basin: 43195 Percentage: 2.840408434885305% Weight: 0.028404084348853054 TotalConditions: 1520732.0

Complex regulation of Hsf1-Skn7 activities by the catalytic subunits of PKA in Saccharomyces cerevisiae: experimental and computational approaches.

A:8 -> Basin: 41668 Percentage: 2.739996264956613% Weight: 0.02739996264956613 TotalConditions: 1520732.0

A:9 -> Basin: 10276 Percentage: 0.6757272155777613% Weight: 0.006757272155777612 TotalConditions: 1520732.0

A:10 -> Basin: 2482 Percentage: 0.1632108747629431% Weight: 0.0016321087476294312 TotalConditions: 1520732.0

A:11 -> Basin: 10713 Percentage: 0.7044633768474655% Weight: 0.007044633768474655 TotalConditions: 1520732.0

A:12 -> Basin: 5163 Percentage: 0.3395075529416097% Weight: 0.003395075529416097 TotalConditions: 1520732.0

A:13 -> Basin: 80 Percentage: 0.005260624488733058% Weight: 5.260624488733057E-5 TotalConditions: 1520732.0
